# Fetching felines: a survey of cat owners on the diversity of cat (*Felis catus*) fetching behaviour

**DOI:** 10.1038/s41598-023-47409-w

**Published:** 2023-12-14

**Authors:** Jemma Forman, Elizabeth Renner, David A. Leavens

**Affiliations:** 1 School of Psychology, University of Sussex, Falmer, BN1 9QH East Sussex UK; 2 Department of Psychology, Northumbria University, Newcastle-Upon-Tyne, NE1 8ST UK

**Keywords:** Psychology, Animal behaviour

## Abstract

Domesticated animals are famous for the ease with which they can accommodate to diverse human environments and roles, but less well-studied is the ease with which domestic animals can manipulate their human caregivers to their own ends. For example, domestic animals may start and end their play behaviour with humans at times of their choice. Here we present the results of a survey of 924 cat owners who report fetching behaviour in 1154 cats. The overwhelming majority (94.4%) of these owners report that fetching emerged in the absence of explicit training. Fetching was primarily first noticed when the cats were less than one year old (*n* = 701) or 1–7 years old (*n* = 415). Cats initiated and terminated fetching bouts more often than did their owners. Thus, cats who fetch demonstrate independent and co-ordinated agency in the onset and maintenance of fetching behaviour with their human partners. Additional findings highlight the diversity of objects fetched and the diversity in household demographics. Our thematic analysis reveals owners’ perspectives on (a) the process of a fetching session, (b) the initial acquisition of fetching, and (c) the circumstantial factors that influence fetching patterns. In summary, cats who fetch largely determine when they engage in fetching sessions and actively influence the play behaviour of their owners.

## Introduction

### Play behaviour

Play behaviour is evident across multiple animal species and is most frequently seen in young animals^1–3^. It is reported mostly in mammal and bird species and is less researched in other organisms^4,5^. Play behaviour is found in domestic animals^4,6–8^ and non-domestic animal species^9–11^. Behaviours can be broadly categorised into locomotor, object and social play^12,13^, with social play often resembling sexual, agonistic or hunting behaviours^1^. Additionally, there is evidence of sex differences in play, for example females display more chasing behaviours and males display relatively more wrestling behaviours, as well as males playing more frequently than females^14–19^. Play may have different functions in different species in terms of maintaining social relationships^1,20^, learning locomotor control^6^, cultivating valuable hunting or survival skills^1,21^ or reducing stress^22^.

Social play in domestic dogs (*Canis lupus familiaris*) is a well-documented behaviour with intraspecific play bouts with other dogs and interspecific play bouts with humans^23–25^. Intraspecific play typically has a competitive element related to dominance, whereas social play with humans is more cooperative and inherently rewarding^23^. Dogs that frequently initiate play with their owners behave with increased dominance and score lower for amenability^26,27^. These play-initiating dogs may also have the freedom to access all areas of a house, demand attention and beg for food from their owners^27^. The ontogeny of object-based play is relatively understudied^28^. Solitary object play resembles predatory behaviour in morphology, with preferred toys being those that can be torn or ripped apart^23^. Play in puppies develops from solitary object play to social object play at approximately 5 weeks^28,29^, with object play in adult dogs continuing to be predominantly seen in social contexts with other dogs or human partners^30,31^. The behavioural structure of object play in puppies is similar to that in adult dogs^29,32^. There are some notable breed differences in solitary play from as young as 7 weeks of age, for example retrievers are more likely to engage in solitary play than livestock-guarding dogs, but social play with other dogs does not appear to differ according to breed^29,33^.

Similarly, play behaviour in domestic cats (*Felis catus*) resembles hunting behaviour in terms of morphology^32,34^. Comparable behavioural sequences include a rapid approach and retreat, leaping, chasing, pouncing, and stalking other littermates or objects^3,35,36^. These behavioural sequences are comparable to those of European wildcats^37^ in addition to their big cat counterparts of lynxes^38,39^ and lions^2^. Playing typically emerges in the first 2–3 weeks of life and is primarily directed towards littermates^3,40^. Although social play in cats is difficult to generalise as it is dependent on the social context, social play largely appears to be flexible and serve different purposes in cats; non-reciprocal fighting play (treating a conspecific as an object or prey) may be useful in developing skills for manipulating the environment, whereas reciprocated fighting play may be useful for social learning and facilitating social relationships^41^. Playing then develops more towards object-based play, primarily between 18 and 21 weeks of life, with a gradual decline in object-based play after 21 weeks^40^. This is the opposite development to that of dogs, who transition first from solitary object-based play to social play^28^. However, there is limited research into how adult cat play is organised and how this may differ from kittens and young cats^42^. Adult indoor-only cats have been found to be more interested in playing with toys than indoor-outdoor cats, possibly due to indoor-only cats being deprived of real prey and therefore showing more intense reactions when presented with a prey substitute^43–45^.

The motivation behind playing with objects in cats is different from that of dogs; cats are more motivated to engage in solitary play with objects that have prey-like features^34,46,47^, as seen in other non-domestic animal species such as kestrels (*Falco sparverius*)^9^, whereas object play in dogs usually involves a dog or human partner and is socially motivated^23,30,31^. Adult cats have been observed to play with objects by themselves, suggesting that object play does not have intraspecific social motivation in cats^3^. Nonetheless, toys are a common way of playing with cats in interspecific interactions between humans and cats, with variation in the features and type of toy that can be used in these play interactions^48^. Cats have been found to prefer toys similar to the size of a mouse^34,46^, toys that are moved erratically instead of being stationary (for example, toys attached to rods)^47^, and toys that can break or pull apart while the cat interacts with them to maintain engagement^42^. Playing has advantages for both pet cats and owners, for example by acting as a substitute for predation on real animals^49,50^, preventing aggression towards humans^51^ and improving owner understanding of cat communication and understanding of the needs of their cat^44^.

### Fetching and retrieval

“Fetching” can be defined as the retrieval of an object, often thrown by a pet owner^23^. Fetching is a very common social bonding and play activity between owners and domestic dogs that is frequently seen in younger dogs^23,24,27,52^. Playing fetch has also been shown to reduce stress and develop motor skills in dogs^52,53^. Dogs have been shown to differentially retrieve objects dependent on the owner’s emotional valence towards certain objects^54^. Dogs are also sensitive to attentional cues such as body posture (facing forwards or backwards) and eye cues (blindfolded or non-blindfolded) in fetching scenarios with their owners^55^. Some dog breeds that score higher on trainability have an increased affinity for fetching and retrieving objects, including the Labrador retriever, Australian shepherd and Doberman pinscher, with the latter two breeds also scoring higher on energy and playfulness than other breeds^56^. Moreover, a recent study reported spontaneous ball retrieval in hand-reared wolf pups to an unfamiliar person^57^.

Conversely, there is limited research that specifically investigates how or why cats fetch objects. In one anecdotal description of the fetching process itself^58^, the owner throws an object for the cat to chase, the cat waits for the owner to get the object themselves and the owner calls the cat’s name to follow them, after which the owner throws the object again. The cat eventually brings the object back to the initial throwing location (although this does not occur every time) and is then met with lots of praise. However, this retrieval behaviour is short-lived as boredom reportedly sets in with both the cat and owner^58^. Fetching behaviour has also been reported by cat owners via questionnaires amongst other tricks such as meowing on command or using the toilet; fetching was reported to be more common than other behaviours such as coming when called or playing games^59,60^. In a qualitative analysis on play behaviour within cat-human dyads, one owner noted that their cat was “obsessed with fetch. He drops toys on my face in the middle of the night. I don’t want to encourage that as I value my sleep!”^44^. Fetching is typically grouped in with other play activities in owner surveys regarding cat-human play behaviour^44,61^, and even general play activities are grouped amongst other cat-owner interactions such as grooming^62^. However, in the few reports of cat fetching behaviour in the literature, it is unclear whether this behaviour was intentionally trained or occurred spontaneously. It has been suggested that fetching in cats requires training and is shaped on a foundation of the cat’s instinctive behaviours^63^.

Fetching has been consistently reported across both mixed and purebred breeds^60^. In particular, the Siamese, Abyssinian and Himalayan (crossbreed of Siamese and Persian) breeds appear to be some of the most proportionally prevalent retrievers^60^. Tonkinese (crossbreed of Siamese and Burmese) and Devon Rex breeds have been scored significantly higher on playfulness in relation to other breeds^64^. Fetching, specifically, has been reported in the temperament descriptions of Bombay, Burmese, Maine Coon, Savannah, Siamese, and Turkish Van cats in cat breed registries (Cat Fanciers’ Association, CFA https://cfa.org/breeds/; The International Cat Association, TICA https://tica.org/). As such, the Siamese and its crossbreed variations are the most common breeds to display fetching and retrieving behaviours across multiple studies^60,64^.

### Present study

Fetching in cats has been the focus of limited research in the scientific literature but is a moderately well-documented behavioural phenomenon in anecdotal evidence^44,58^ and in reference to specific cat breeds on cat breed registries (for example, CFA, TICA). We surveyed an international sample of cat owners to explore questions of how fetching first occurs, including whether the cat was intentionally trained, who usually initiates and ends a fetching session, the age of the cat when they first began to fetch and the types of objects that are fetched. We provide descriptive statistics of cat, owner and household demographics and inferential statistics regarding the frequency of fetching sessions per month and number of retrievals in the most recent session. We also asked cat owners to describe the first time they noticed their cat fetching an object, the responses of which were assessed in a qualitative, thematic analysis to get a richer insight into the process of how these cats began to fetch.

Fetching is a paradigmatic example of coordinated joint engagement, a hallmark of development in Western children^65^. Substantial contemporary debate exists over the capacity of animals to display such coordinated activity in the absence of overt training; there is a prevailing view that such triadic cognitive skills are distributed sparsely in the animal kingdom, to humans and highly domesticated animals like dogs^66^. It is therefore of significant scientific interest whether cats, who have been domesticated to a lesser degree than dogs^67^, (a) can fetch without explicit shaping and (b) contribute independent agency to the initiation and termination of fetching bouts. We were also interested in gaining an understanding of the environmental correlates of fetching in domestic cats. Finally, we wanted to give voice to the owners, considering them as the observers best placed to interpret their cats’ behaviour in developmental context.

## Methods

### Materials

We created an online questionnaire using Qualtrics (Qualtrics, Inc., Provo, UT, USA). The survey was designed to focus specifically on when owners first noticed the fetching behaviour in their cat. Owners could answer for either a cat they owned in the past or a cat they currently own. The final survey (Supplementary Information S1) consisted of 23 questions relating to: intentional training of the cat (Q5); the objects their cat will fetch (Q6, Q6a); who initiates and ends the fetching sessions (Q7, Q8); and the number of times the cat will fetch in single sessions and on a monthly average (Q9, Q10, Q11). The survey also had two open-ended questions for owners to describe how they trained or first noticed the fetching behaviour (Q5a, Q5b). Cat demographic data were collected for the time the fetching behaviour was first seen (age, sex, neuter status, breed, multi-cat household). Owner and household demographic data were also collected for the time the fetching behaviour was first seen (country, number of people in the household, age, gender, dogs present in household, other pets in the household).

### Participants

An international sample of survey respondents was recruited via online social media platforms, including Twitter, Facebook and Reddit. A total of 1258 owners consented to fill out the survey prior to data cleaning. Responses were excluded if not completed in their entirety (*n* = 334), leaving a total of 924 owner responses for 1184 cats. Cats with extreme or exaggerated answers were excluded (*n* = 2). Cats with ambiguous or contradicting responses on the same question were excluded (*n* = 23). Lastly, one duplicated entry (*n* = 1) and two duplicated entries with contradicting responses were removed (*n* = 4), leaving a total of 1154 cats in the final sample.

The final sample included responses from North America (*n* = 813), Europe (*n* = 265), Australasia (*n* = 34), South America (*n* = 22), Asia (*n* = 13), and Africa (*n* = 4); two respondents noted they and their cat(s) had lived in more than one country. Participants were asked to complete the questionnaire as of the time they first noticed their cat fetching, of which 40 cats first fetched when the respondent was under 18 years old. Respondents were a mean age of 34.73 years (SD = 11.18, range 4–73 years as of their cat’s initial fetching bout) and identified as female (*n* = 748), male (*n* = 266) or non-binary (*n* = 109) with some respondents preferring not to say (*n* = 31).

### Data cleaning

Cats who had a clear breed, for example ‘Siamese’ or ‘Bengal’, were grouped into their respective purebred category. Purebreds were grouped according to the guidelines from The Cat Fanciers’ Association (https://cfa.org/breeds/). All other cats were categorised as ‘mixed breed’ as most owners did not say with certainty which breed their cat is; this was the case, for example, if their cat was described with the terms ‘domestic shorthair’, ‘domestic longhair’, ‘mixed’, ‘cross-breed’, ‘moggy’, or ‘hybrid’, or if they described their cat using only colours (e.g., ‘orange cat’) or coat patterns (e.g., ‘tabby’). Cat life stages were categorised into four age groups of kittens (< 1 year), young adult (≥ 1 years and < 7 years), mature adult (≥ 7 years and < 10 years) and senior (≥ 10 years) based on the AAFP-AAHA feline life stage guidelines^68^.

For the questions regarding cat’s favourite object to fetch (Question 6a), we had originally wanted to ask respondents to select as many favourite items as they wanted and allow for multiple answers. However, due to the answer type requirements in the survey software being mistakenly saved as only allowing one answer, this question allowed for only one object to be selected instead of the originally anticipated multiple objects. We therefore interpreted responses to this question as owners selecting the cat’s single most favourite object to fetch. Each favourite object was subsequently grouped into one of 14 categories: toys (for humans or cats); spherical objects (e.g. crumpled paper, aluminium foil balls); cosmetics (e.g. hair ties, Q tips); arts and crafts (e.g. pom poms, pipe cleaners/chenille stems); bottle parts (e.g. lids, ring pulls); stationery (e.g. pens, paper clips); consumables (e.g. chew sticks, green pea); springs; twist ties (e.g. bread ties, cable ties); string-like objects (e.g. shoelaces, string); clothing items (e.g. gloves, shoes); ring-shaped objects; scraps (e.g. leather or fabric); and miscellaneous. Any favourite object that was not specifically referred to as a ‘toy’ was grouped in its most salient category, for example ‘tin foil ball’ would be grouped with spherical objects and ‘plastic spring’ would be grouped with ‘springs’. Each favourite object was grouped into only one category.

### Statistical analysis

Descriptive statistics were created of household, owner and cat demographics (see Results). Responses with conflicting answers were removed from the specific descriptives of number of dogs in the household (*n* = 3), number of cats in the household (*n* = 65) and number of other fetching cats in the household (*n* = 65).

Data were analysed and figures created using R software (version 2022.07.2 + 576, https://cran.r-project.org/) using the packages car, coin, DescTools, gmodels, qqplotr, rstatix, sf and tidyverse. R code is available in Supplementary Information S2 and the dataset used is available in Supplementary Information S3. Normality checks were carried out using Shapiro–Wilk tests and assessing QQ plots prior to statistical analysis. Extreme outliers above Q3 + 3 × IQR and Q1 − 3 × IQR were identified. The Shapiro–Wilk tests determined the distributions of the data were significantly different from a normal distribution, thus non-parametric tests were used. Statistical analyses were carried out both including and excluding the extreme outliers; there were no differences in the interpretation of the findings and so the analyses retaining the outliers are reported in the Results section.

Chi-square tests were used to compare frequency counts in who initiated fetching sessions on average (owner, about equal, cat), who ended fetching sessions on average (owner, about equal, cat), and the numbers of female and male cats. Where the Chi-square was significant for variables with more than two levels, three additional Chi-squares were carried out as post-hoc comparisons that compared one level with one other level and were interpreted using a corrected alpha of 0.016 (0.05/3) to reduce Type I error. Chi-square tests were used to compare differences between cat sex (male and female) in who initiates the fetching sessions (owner, about equal, cat) and who ends the fetching sessions (owner, about equal, cat). Kruskal–Wallis tests were used to assess differences between the initiator(s) and ender(s) of the fetching sessions in the number of retrievals in the most recent session, as well as the number of monthly fetching sessions. Mann–Whitney U tests were used to compare differences between cat sex in the number of retrievals in the most recent session, as well as to compare differences between cat sex in the number of monthly fetch sessions. Levene’s tests were used to determine the most suitable adjustment method in post-hoc analyses which showed there were not equal variances (*p* < 0.05). Thus, pairwise Wilcoxon rank sum tests with Bonferroni corrections were used in post-hoc analyses on significant differences.

### Qualitative analysis

A bottom-up thematic analysis was carried out on open-ended questions for the qualitative descriptions of how the cat started to fetch and extra comments by the owners (Q5a, Q5b and Q12). The procedure of the thematic analysis followed the steps outlined in published guidance^69^. Initially, the first author (J.F.) familiarised themself with the qualitative responses by reading and re-reading the text descriptions. First-order coding was then carried out in NVivo (R1.6) to identify and label initial descriptive concepts from the text descriptions. Initial codes (*n* = 268) with 35 subsidiary (“child”) codes were identified at this stage. This was followed by second-order coding with the clustering of related codes to uncover super-ordinate themes. The clustering of related codes was carried out using thematic maps as an aid for the visualisation of the overall themes. Themes were reviewed by (a) reading and assessing the text descriptions in each theme to ensure coherency and internal consistency within the theme itself and (b) comparing the themes with the overall text descriptions to ensure the appropriate themes had been extracted. Relevant themes and sub-themes were selected, clearly defined and named by the authors at the end of this stage (see Supplementary Fig. S4 for the progression of the thematic map).

### Ethical statement

This study was approved by the Science and Technology Cross-Schools Research Ethics Committee at the University of Sussex (project reference: ER/JF337/1). All methods were performed in accordance with the relevant guidelines and regulations. Informed consent was obtained from all participants prior to viewing the questionnaire.

## Results

### Cat and household demographics

**Figure 1:**
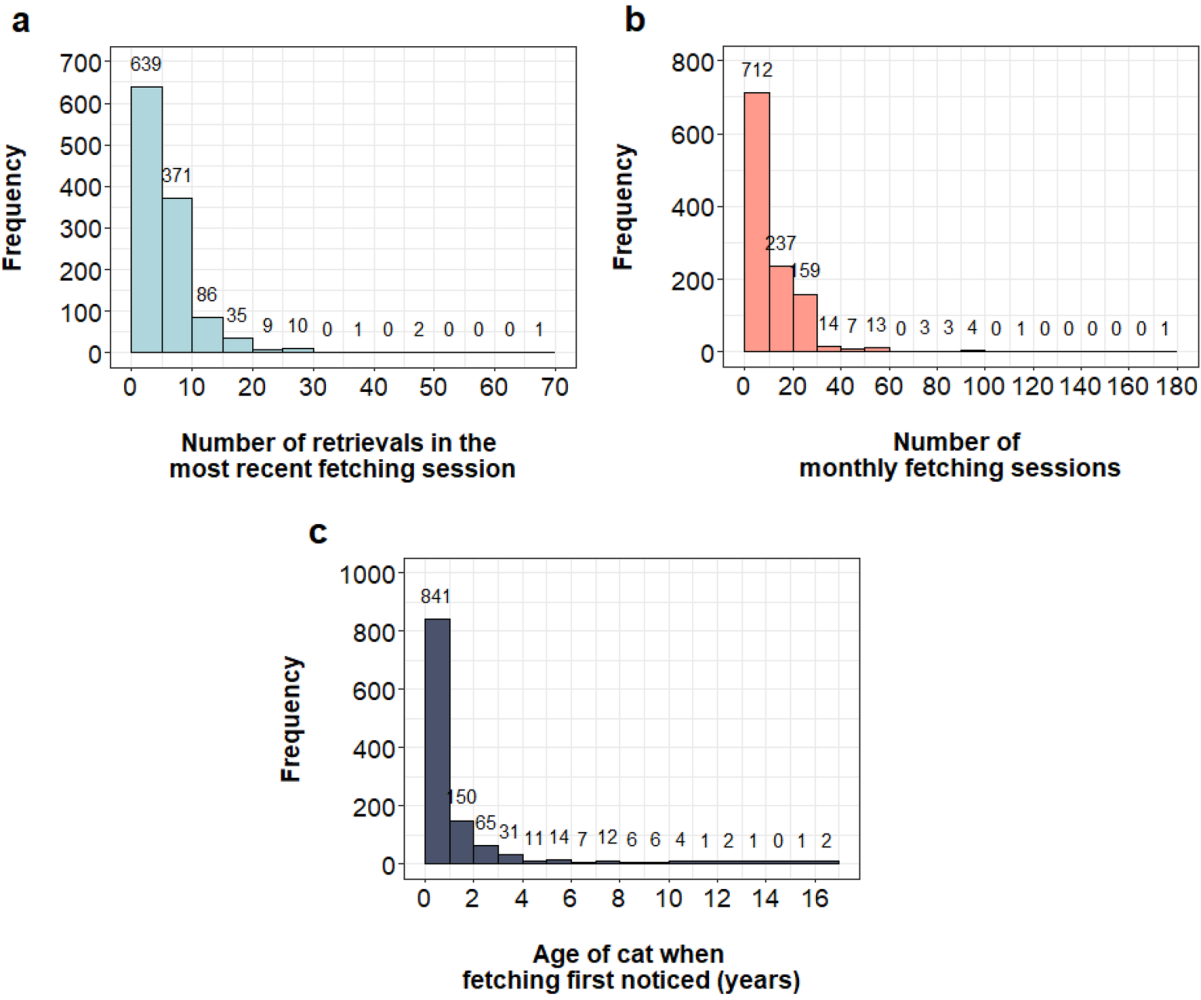
Histograms illustrating (**a**) the number of retrievals in the most recent fetching session, (**b**) the number of fetching sessions per month and (**c**) the age of the cat when fetching was first noticed. Frequency counts are presented above each bin. Four cats were reported to retrieve 0 times in the most recent session, 35 cats had less than 1 monthly fetching session and 12 cats were reported to be less than one month old when fetching was first noticed.

The majority of owners reported fetching behaviour by current cats (*n* cats = 853), while a substantial minority reported fetching behaviour by previous cats (*n* cats = 301). There were significantly more male fetching cats (*n* = 617) than female fetching cats (*n* = 537), *X*^2^ = 5.50 (1, *N* = 1154) *p* = 0.018. Most cats were younger than 1 year when their owner first noticed the fetching behaviour (*n* = 701), followed by cats between the ages of 1 and 7 years (*n* = 415). Fewer cats first displayed their fetching behaviour at 7 years and above (*n* = 38). The median age of the cat when fetching was first noticed is 7 months (25th percentile = 5 months, 75th percentile = 16 months; range = 0–204 months). Most cats fetched 1–5 times in the most recent fetching session (*n* = 635) and had 1–10 fetching sessions per month (*n* = 677). Figure 1 illustrates the dispersion of data for the number of retrievals in the most recent session, number of monthly fetching sessions and the age of the cat when fetching was first noticed. A vast majority of fetching cats (94%) were untrained (*n* = 1089) while a minority were intentionally trained (6%, *n* = 65). Most cats were identified as mixed breed (*n* = 994), with 160 cats identified as purebred. Siamese were the most frequently reported fetching purebred (*n* = 36), followed by Bengal (*n* = 16) and Ragdoll (*n* = 12). A full list of the frequency of all purebreds in our sample can be found in Supplementary Table S1.

Most fetching cats did not share a household with a dog (*n* = 989). Out of the cats that did share a household with a dog (*n* = 162), only 106 lived with at least one fetching dog. In contrast, most fetching cats did share a household with at least one other cat (*n* = 638). However, for the majority of cats who lived with other cats, the other cat(s) did not fetch (*n* = 479). A summary of owner and household demographics is presented in Table 1 and a full summary of cat and fetching demographics is presented in Table 2.

**Table 1:** Descriptive statistics of household and owner demographics (*N* = 1154 cats).

Respondent gender	Mean respondent age in years (SD)	Number of people in household	Mean age of household in years (SD)
Female 748 (64.8%)	34.69 (11.41) 6–73	1: 220 (19.1%)	34.80 (9.78) 20–73
		2: 565 (49%)	35.59 (10.91) 0–72
Male 266 (23.1%)	37.22 (11.13) 4–64	3: 212 (18.4%)	36.25 (17.95) 0–88
Non-binary 109 (9.4%)	29.72 (7.52) 7–48	4: 100 (9.5%)	31.63 (17.69) 0–86
		5: 39 (3.4%)	33.55 (20.61) 1–87
Prefer not to say 31 (2.7%)	27.09 (7.97) 15–40	6: 6 (0.5%)	24.78 (16.12) 2–64
		7: 2 (0.2%)	27 (18.68) 10–73

Number of dogs in household	Number of fetching dogs in household	Number of cats in household	Number of other fetching cats in household
0: 989 (85.9%)	0: 56 (34.6%)	1: 451 (41.4%)	0: 479 (75.1%)
1: 103 (8.9%)	1: 81 (50%)	2: 383 (35.2%)	1: 134 (21%)
2: 47 (4.1%)	2: 23 (14.2%)	3: 156 (14.3%)	2: 22 (3.4%)
3+: 12 (1%)	3: 2 (1.2%)	4: 46 (4.2%)	3: 3 (0.5%)

Would cat have seen the dog fetch prior to fetching themselves?		5: 26 (2.4%)	
Yes: 34 (32.1%)		6: 12 (1.1%)	
No: 72 (67.9%)		7 +: 15 (1.4%)	

Frequencies and percentages are presented along with the mean, SD (in brackets) and ranges of age and duration.

**Table 2:** Descriptive statistics of cat and fetching demographics (*N* = 1154 cats).

Cat sex and neuter status	Cat status	Training status	Breed
Female spayed 501 (43.4%)	Previous cat 301 (26.1%)	Untrained 1089 (94.4%)	Mixed 994 (86.1%)
Female intact 36 (3.1%)	Current cat 853 (73.9%)	Trained 65 (5.6%)	Purebred 160 (13.9%)
Male neutered 601 (52.1%)			
Male intact 15 (1.3%)			
Male unknown 1 (0.1%)			

Cat age in months at time fetching first noticed (SD)	Life stage at time fetching first noticed	Duration of time in months the cat had fetched for (SD)	
15.90 (23.53) 0–204	Kitten (< 1 year) 701 (60.7%)	51.53 (48.72) 0–244	
	Young adult (< 7 years) 415 (36%)		
	Mature adult (< 10 years) 22 (1.9%)		
	Senior (≥ 10 years) 16 (1.4%)		

Count of who initiated the fetching sessions on average		Count of who ended the fetching sessions on average	
Cat	553 (47.9%)	Cat	675 (58.5%)
Owner	249 (21.6%)	Owner	248 (21.5%)
About equal	352 (30.5%)	About equal	231 (20%)

Frequencies and percentages are presented along with the mean, SD (in brackets) and ranges of age and duration.

### Favoured objects

**Figure 2:**
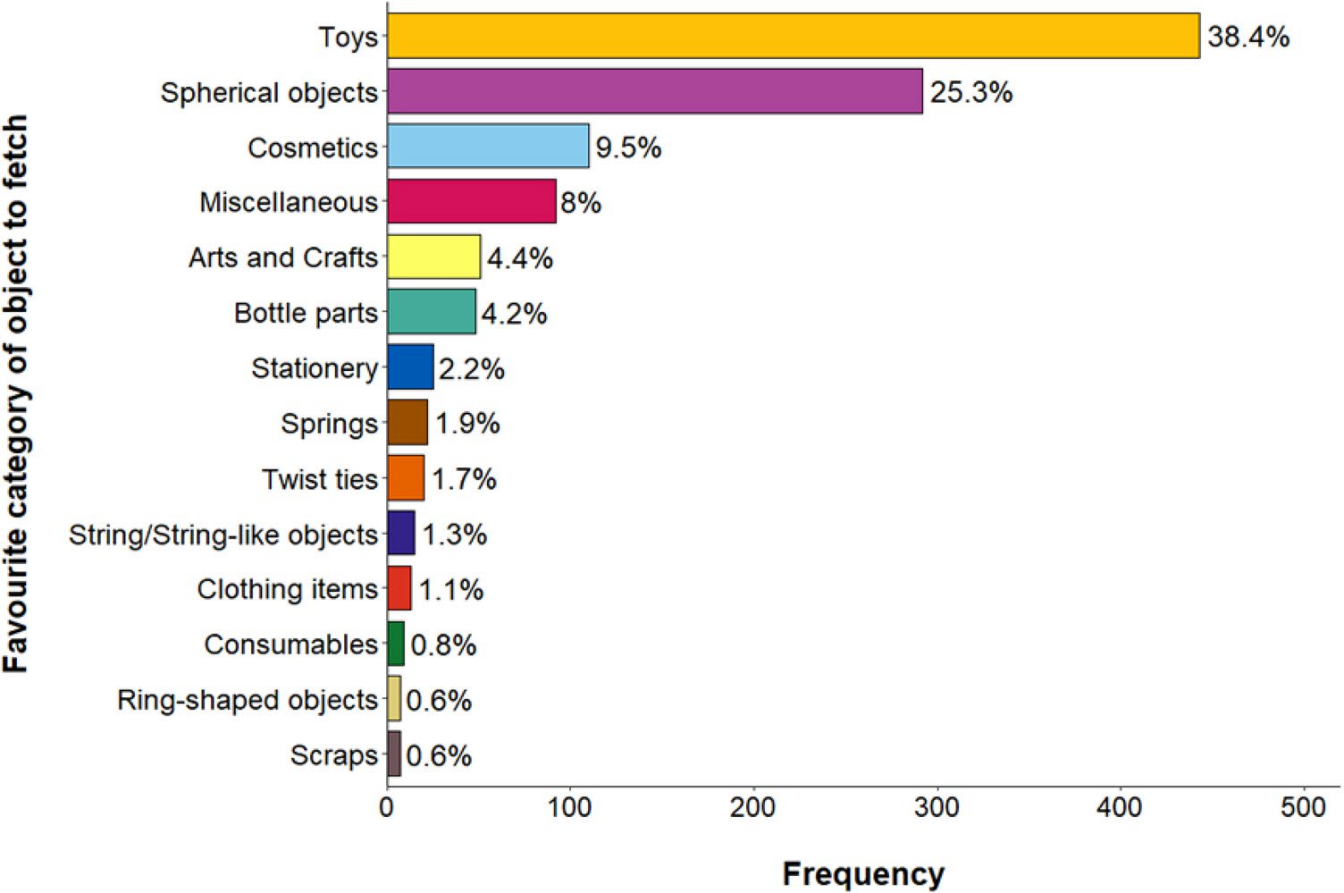
Horizontal bar chart illustrating cats’ favourite object categories to fetch (*N* = 1154). Percentages are presented alongside each category.

Cats’ favourite categorised objects to fetch are presented in Fig. 2, and a spreadsheet of all of the objects cats fetched is in Supplementary Information S6. Cat toys were the most frequently cited favourite object type, followed by spherical objects and cosmetics.

### Initiation and termination of fetching sessions

There were unequal distributions of who initiates fetching sessions on average (cat, about equal, or owner) (*X*^2^ = 124.29 (2, *N* = 1154) *p* < 0.001). Post-hoc comparisons demonstrated that cats (*n* = 553) initiate fetching sessions more than owners (*n* = 249), (*X*^2^ = 115.23 (1), *p* < 0.001) and more than when initiation was about equal (*n* = 352), (*X*^2^ = 44.64 (1), *p* < 0.001). Owners were significantly less likely to initiate than both the cat and owner equally (*X*^2^ = 17.65 (1), *p* < 0.001).

**Figure 3:**
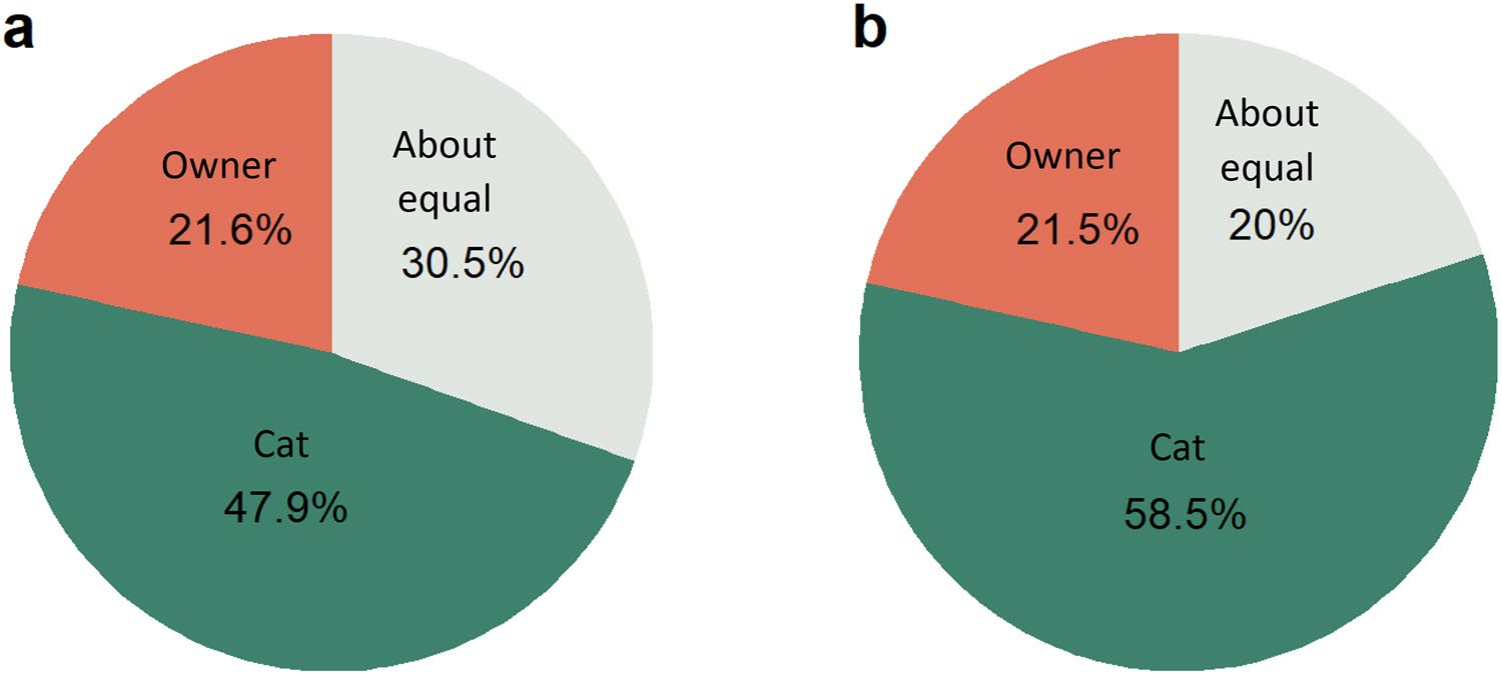
Pie charts illustrating who the owner considered to (**a**) initiate fetching sessions on average and (**b**) end fetching sessions on average (*N* = 1154). Red segments represent the owner, green segments represent the cat and grey segments represent both the cat and owner as equals. Percentages are presented within their respective segment.

There were also significant differences between who ends the fetching sessions on average (*X*^2^ = 329.08 (2), *p* < 0.001). Cats (*n* = 675) terminate fetching sessions more than owners (*n* = 248), (*X*^2^ = 197.54 (1), *p* < 0.001) and more than when the termination was considered equal (*n* = 231), (*X*^2^ = 217.59 (1), *p* < 0.001). There was not a significant difference in frequency between the owner and both the cat and owner equally terminating the session (*X*^2^ = 0.60 (1), *p* = 0.437). Thus, cats were depicted by their owners as more frequently the agent that controlled the initiations and terminations of fetching sessions. Figure 3 illustrates the relative proportions of who initiates and who ends the fetching sessions on average.

### Number of retrievals in most recent session

There were significant differences between who usually initiates the fetching sessions in the number of retrievals in the most recent fetching session (Kruskal–Wallis *X*^2^ = 13.98 (2, *N* = 1154), *p* < 0.001). There are significantly fewer retrievals if the owner usually initiates the sessions (median = 5, IQR = 4) compared to the cat (median = 5, IQR = 6, Pairwise Wilcoxon *p* = 0.009) or if initiation is about equal (median = 6, IQR = 6, Pairwise Wilcoxon *p* < 0.001); there is no difference in retrieval number between when the cat usually initiates and when initiation is about equal (Pairwise Wilcoxon *p* = 0.995).

There were significant differences between who usually ends the fetching sessions in the number of retrievals in the most recent session (Kruskal–Wallis *X*^2^ = 110.55 (2, *N* = 1154), *p* < 0.001). There are significantly more retrievals when the owner ends the sessions (median = 8, IQR = 5) in comparison to when the cat ends the sessions (median = 5, IQR = 4, Pairwise Wilcoxon *p* < 0.001) or when both the cat and owner equally end the fetch sessions (median = 6, IQR = 6, Pairwise Wilcoxon *p* < 0.001). There are significantly more retrievals when both the cat and owner equally end the fetch sessions in comparison to when the cat ends the sessions (Pairwise Wilcoxon *p* < 0.001).

### Number of monthly fetching sessions

There were significant differences between who usually initiates the fetching sessions in the number of monthly fetching sessions (Kruskal–Wallis *X*^2^ = 52.95 (2, *N* = 1154), *p* < 0.001). There are significantly fewer monthly sessions if the owner usually initiates the sessions (median = 4, IQR = 8) compared to the cat (median = 10, IQR = 16, Pairwise Wilcoxon *p* < 0.001) or if initiation is about equal (median = 10, IQR = 16, Pairwise Wilcoxon *p* < 0.001), but no significant difference between the cat and if initiation is about equal (*p* = 0.66).

There are significant differences between who usually ends the fetching sessions in the number of monthly sessions (Kruskal–Wallis *X*^2^ = 62.97 (2, *N* = 1154), *p* < 0.001). There are significantly fewer fetching sessions per month if the cat usually ends the sessions (median = 6, IQR = 12.5) in comparison to the owner (median = 10, IQR = 15, Pairwise Wilcoxon *p* < 0.001) or if ending is about equal (median = 12, IQR = 20, Pairwise Wilcoxon *p* < 0.001). There were no differences in the number of fetching sessions per month between the owner ending the sessions or whether this was about equal (Pairwise Wilcoxon *p* = 1.00).

### Cat sex

There were no significant differences between male and female cats on who usually initiates the fetching sessions (*X*^2^ (2) = 1.82, *p* = 0.403) or who usually ends the fetching sessions (*X*^2^ (2) = 1.97, *p* = 0.374). There were no significant differences between male and female cats on the number of retrievals in the most recent fetching session (Mann–Whitney *Z* =  − 1.69, *p* = 0.091) or the number of fetching sessions per month (Mann–Whitney *Z* =  − 0.49, *p* = 0.622).

### Qualitative analysis

**Figure 4:**
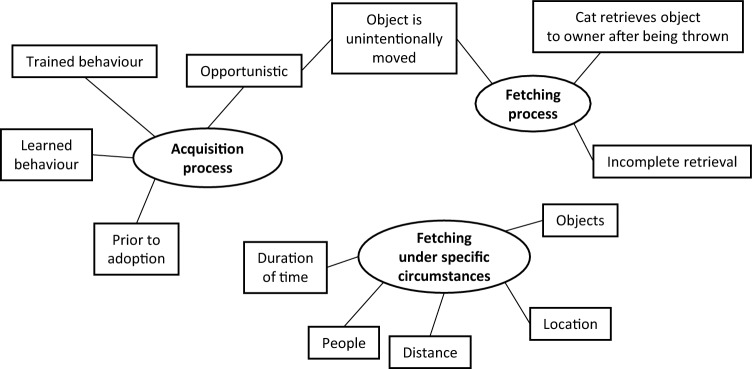
Final thematic map illustrating three main themes (ovals) with branching sub-themes (boxes).

The analysis generated three themes that are illustrated in Fig. 4. One quotation is presented per theme and additional quotations are available in Supplementary Information S7.

#### Fetching process

This theme captures some of the behavioural aspects of the fetching procedure and how and when objects would be retrieved to the owner. Respondents often stated they threw an object (intentionally or unintentionally) after which the cat brought it back to them. Other respondents also stated their cat brought the object to them first after which the owner then threw the object. Cats were noted to have incomplete retrievals, for example in dropping the object halfway between where it landed and where it was first thrown, or in gradually dropping the object further and further away from the owner. This theme also captures how fetching developed from playing and how owners reported they unintentionally threw, dropped or moved objects which also led to the occurrence of fetching. One respondent noted that they unintentionally moved an object and how their cat reacted:

“In opening a newspaper, the rubber band slipped off and flew down the hallway. Waldo chased it down and brought it back, dropping it at my feet. I 'shot' it again, and again he fetched it.”

#### Acquisition process

This theme captures how the owner first noticed their cat beginning to fetch and retrieve items. Some owners stated they knew their cat was able to fetch prior to adoption (for example, if adopted from a family friend or from a shelter that knew about this behaviour). A small proportion of owners intentionally trained their cat to fetch objects using clicker training or reward-based techniques with food or social rewards. Some owners believed their cats could have learnt to fetch from other animals (including siblings, parents, offspring, unrelated conspecifics, and dogs) or that the cat trained them to play fetch. However, many owners stated their cat opportunistically started fetching or retrieving objects to them. One respondent stated how their cat initiated the fetching process of their own volition:

“One day he brought the toy back to me, completely unprovoked, dropped it next to me, and sat waiting for me to throw it again.”

#### Fetching under specific circumstances

This theme describes how some cats would fetch only under specific circumstances related to the nature of the fetching session. Some cats would fetch an object only if the object was thrown far enough away from the initial thrown location. Some cats were reported to fetch only a few times in one session, whereas others were reported to fetch obsessively for great lengths of time. Some cats were interested in fetching only one specific object, despite, for example, the owner providing an identical toy of a different colour which the cat would not play or fetch with. Some cats would fetch only in specific locations of the house; two common locations were the bedroom (of which cats were sometimes reported to drop the object in the owner’s bed while the owner was asleep) and throwing the object using the stairs for the cat to chase up and down. Some cats were also reported to fetch only with specific people in the household. One respondent reported how the fetched object has to be of a certain size:

“The size of the pom pom is important. I bought a larger pom pom and she rejected it. I've also tried small items approximately the same size as the pom pom and she rejects those as well.”

## Discussion

Our survey of 924 owners of fetching cats reveals that fetching is a diverse cat behaviour evident across multiple countries. For the majority of cats who fetch (94.4%), it is not a trained behaviour. Most cats who fetch first display this behaviour as kittens or young adults. Cats have individual preferences for a variety of objects to fetch and they exhibit agency in the initiation and ending of fetching sessions. Owners also provided detailed descriptions of the fetching process itself and highlighted how some cats will fetch only in certain circumstances.

Cats had fewer retrievals in the most recent session if the owner (vs the cat) usually initiates the sessions. Similarly, cats had fewer sessions per month if the owner (vs the cat) usually initiates the sessions. Comparatively, other research has found that if the owner initiates play, including fetch, the cat had the lowest total daily play time^44^. On the other hand, cats had more retrievals in the most recent session and more fetching sessions per month if the owner ended the sessions on average. This suggests that cats are largely in control of the fetching sessions; when cats initiate sessions, they have more retrievals in the most recent session and more sessions per month. When cats end sessions, they have fewer retrievals and fewer monthly sessions. This may be interpreted as some owners being forced to end sessions because the cat will otherwise fetch for a lengthy period of time; owners of other cats may be continually encouraging the cat to keep playing for longer than the cat themselves would have done otherwise. A previous study had similarly shown that cat-human interactions have shorter durations if the owner is the one to successfully initiate an interaction, in comparison to longer durations if the cat is the one to successfully initiate an interaction^70^, which is reflected in our findings in the increased number of retrievals and monthly sessions when cats initiated the fetching. Additionally, in one survey on play and dominance in dogs, 58% of dogs had been found to be the initiator of fetch^27^, whereas our results identified that 47.9% of cats were the initiator. When dogs control the initiation of a play session, they behave with increased dominance over their owner and may attempt to manipulate their owners in other aspects, for example by demanding attention and begging for food^26,27^. These are familiar traits for cats who often beg for food and demand attention from their owners, leaving owners to also feel a loss of control over feeding time^71,72^. This perceived sense of control from the cat’s perspective, particularly when initiating a fetch session, may also be beneficial for the cat’s welfare^73^ and the cat-owner relationship. Owners being receptive to the needs of their cat by interpreting their behaviours and engaging in reciprocated social play may lead to a more harmonious cat-owner relationship^74^.

There were significantly more male cats than female cats in our sample. However, male and female cats did not differ in who initiated or ended the fetching sessions on average, or the number of retrievals in the most recent session or the number of fetching sessions per month. Previous research has found that males play more frequently than females across multiple domestic and non-domestic species^14–19^. In domestic cats, males display more play behaviours in all-male groups than females in all-female groups^14^. Sex differences in fetching were not found in our results likely due to fetching being a dyadic play behaviour between owner and cat that does not typically involve multiple cats. Most fetching cats were not housed with other fetching cats (75.1%).

Dedicated cat toys accounted for just under 40% of the cats’ favourite objects to fetch. Instead, other objects made or thrown opportunistically (such as crumpled paper) or other random objects around the house (such as hairties, bottle parts) make up the majority of the favourite objects to fetch. Miscellaneous items also made up 8% of the cats’ favourite objects to fetch, including cat costumes, cigarette packets, earplugs, straws and playing cards. This demonstrates that cats are capable of fetching a highly diverse range of objects that are available to them and are not limited to objects specifically made for cats or objects that have only prey-like qualities, such as being easily torn apart^42^.

While a minority of fetching cats lived in households with either dogs who fetched (*n* = 106) or other fetching cats (*n* = 159), the majority did not live with other such animals. Some owners described the possibility that their cat could have learned the fetch behaviours from another animal; however, not all cats in the sample necessarily had such an opportunity. It should be noted that social learning in cats is relatively understudied and there is limited direct evidence of cats copying from other species^75,76^. Thus, the extent to which fetching specifically is socially learned (from other animals) versus self-constructed is yet to be determined.

Our sample contained 160 purebred cats. The Siamese breed was the most frequently reported fetching cat out of all the purebreds (*n* = 36). Despite the relatively small number of purebred cats in our sample, this is consistent with what previous research has found in relation to the Siamese and crossbreed variations being amongst the most common fetching breeds^60,64^. However, fetching is by no means restricted only to certain breeds of cats, as indicated by the large majority in our sample of 994 mixed breeds. It is important to note that many owners did not know or state the exact breed of their cats and as such this is a limitation of the present research.

To target our specific research questions, our survey was designed only for owners who currently own or previously owned fetching cats. While our survey identified a large number of fetching cats, we do not have information about the overall prevalence of this behaviour in the species. Future studies should aim to assess the prevalence of fetching in the wider cat population by including non-fetching cats and recruiting a more representative sample of purebred cats to help determine whether fetching is more common amongst the Siamese purebred or crossbreed variations, as has been supported in our findings and in previous research^60,64^.

Owners were asked to write their own descriptions of how they first trained their cat to fetch or how they first noticed their cat fetching objects. As a result of this, it was not clear who first displayed interest in fetching—whether this was solely the owner, solely the cat or a combination of both the owner and the cat. Some owners described the first fetching instance as themselves throwing the object and the cat subsequently retrieving the object, whereas other owners described themselves throwing the object after the cat had first brought them an object. To understand the exact process of this initial fetching bout, future research should consider asking specific questions on who first displayed interest in the fetching process. Furthermore, it should be noted that some cats who were known to fetch prior to their current ownership have an unknown history of being either intentionally trained by their previous caregiver or untrained.

In summary, our results demonstrate there is lots of individual variation in cat fetching behaviour regarding the type of object retrieved, the number of retrievals and the number of fetching sessions per month. Fetching is not restricted by the sex, breed or location of the cat. An overwhelming majority of cats were untrained in this behaviour and most were kittens or young adults when fetching was first noticed. The agency of fetching lies predominantly with the cat, who is largely in control of a fetching session with their owner and determines how exactly they wish to participate in the fetching session. Owners who are receptive to their cat’s initiation attempts may have stronger bonds with their cats. Future studies should investigate cat fetching behaviour in an experimental setting to provide further evidence and knowledge on the purpose and motivation of fetching in cats.

### Supplementary Information

Supplementary Information 1.

Supplementary Information 2.

Supplementary Information 3.

Supplementary Information 4.

Supplementary Information 5.

Supplementary Information 6.

Supplementary Information 7.

## Data Availability

All data generated or analysed during this study are included in this published article (and its Supplementary Information files).
