# Supplementary material for: Fetching felines: a survey of cat owners on the diversity of cat (Felis catus) fetching behaviour
Source: Sci Rep. 2023 Dec 14;13:20456. doi: 10.1038/s41598-023-47409-w (PMC10721921; doi:10.1038/s41598-023-47409-w)
Supplement: Supplementary file 1 — Supplementary Information 1. [file 41598_2023_47409_MOESM1_ESM.docx]

S1: Example of survey filled out by an owner who has one current fetching cat, “Cat”, and lives in a single-person household.

[1] How many cats have you owned that play fetch? (both in the past and any current cats you own)

[2] What is cat number 1's name?

[3] Is cat number 1 a previous cat you had in the past or a cat that currently lives in your household?


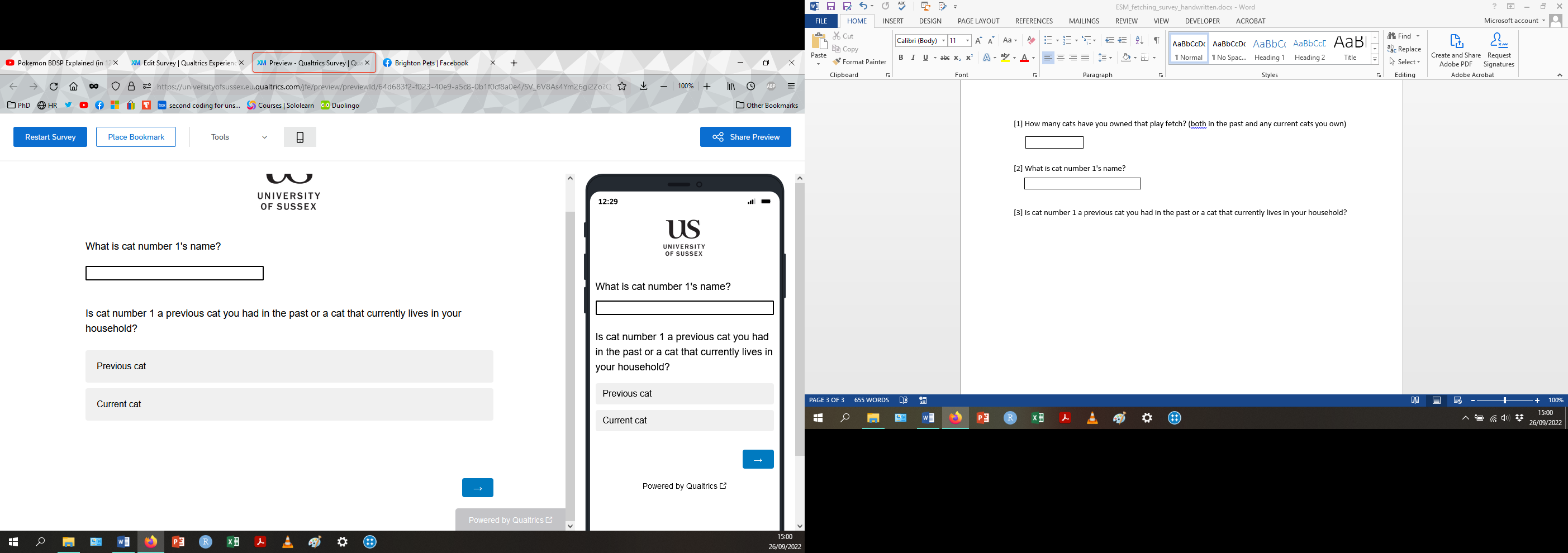


Answer each question as accurately as you can. If you are unsure on any questions, please write 'NA' in the answer box to continue. Please be assured there is no right or wrong answer to any of these questions.

The following questions relate to **Cat**.

[4] Approximately how long has **Cat** fetched? If less than a year, please write '0' in the Years answer box.

Years

Months

[5] Did you deliberately train **Cat** to fetch?


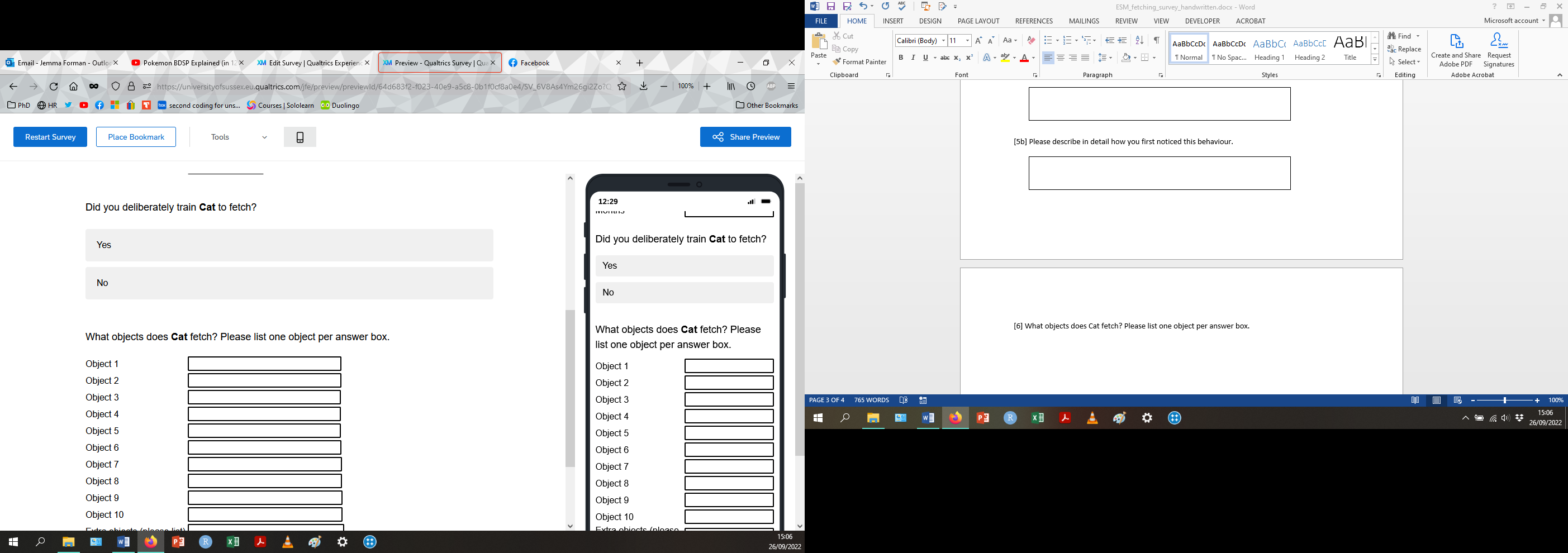


[5a] Please describe in detail how you initially trained this cat.

[5b] Please describe in detail how you first noticed this behaviour.

[6] What objects does **Cat** fetch? Please list one object per answer box.


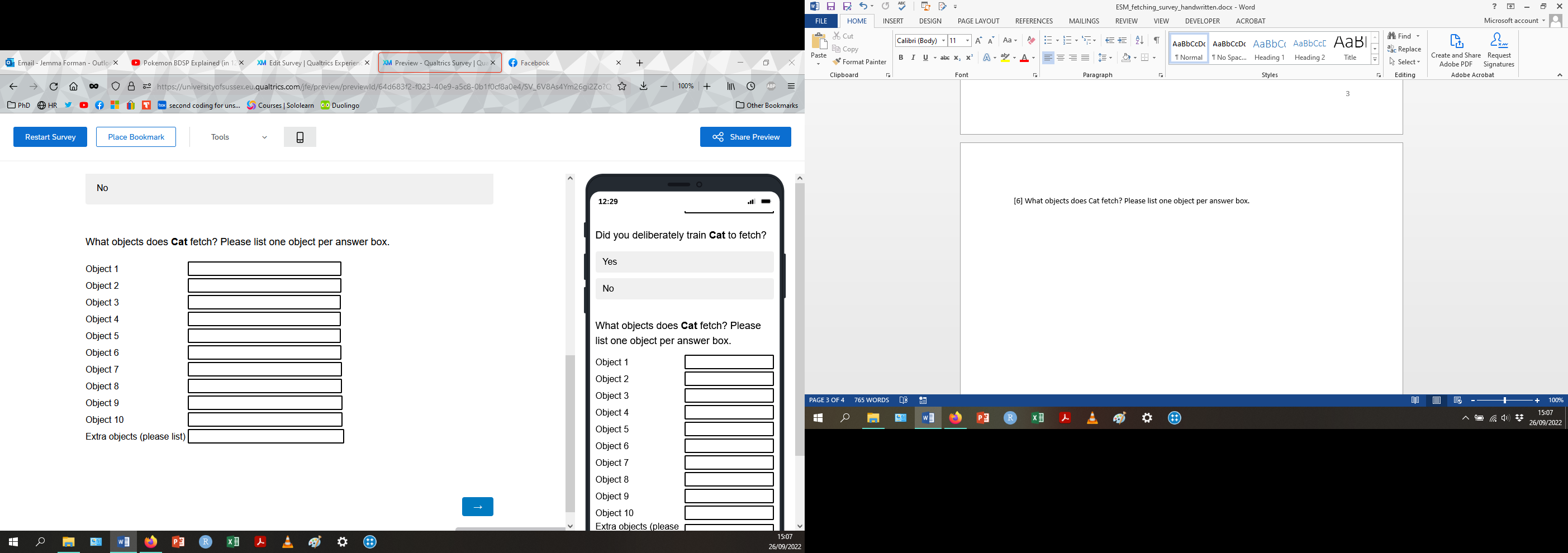


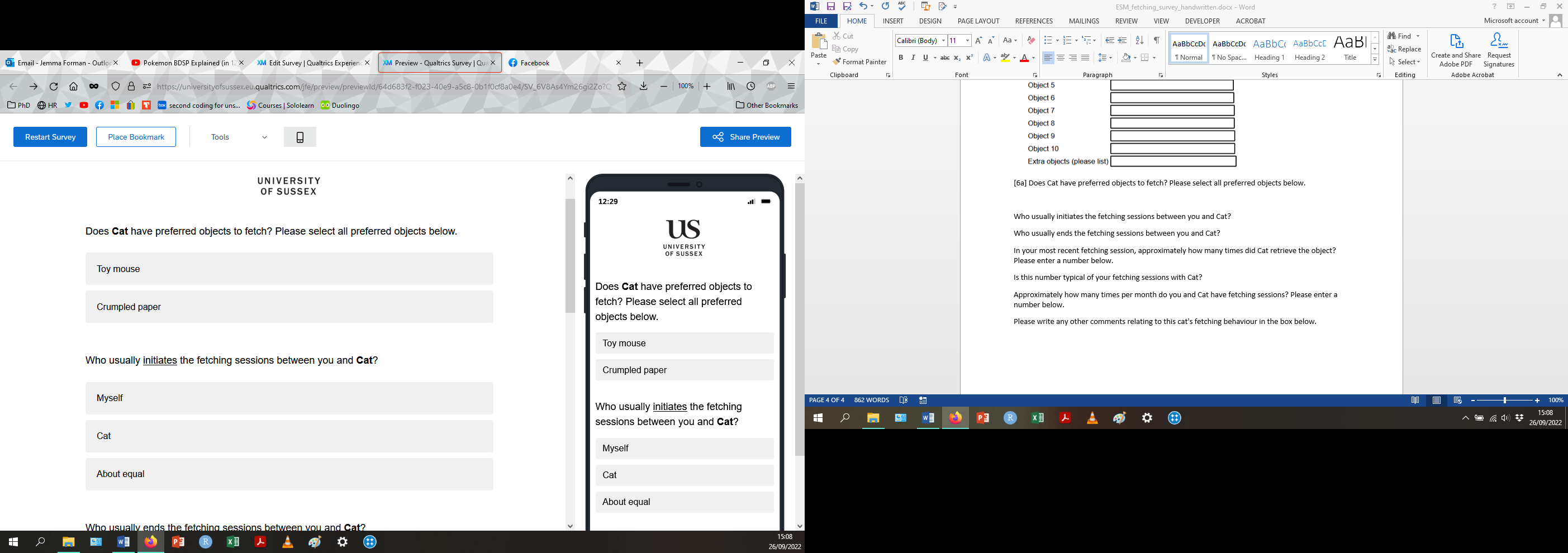
[6a] Does **Cat** have preferred objects to fetch? Please select all preferred objects below.

[7] Who usually initiates the fetching sessions between you and **Cat**?


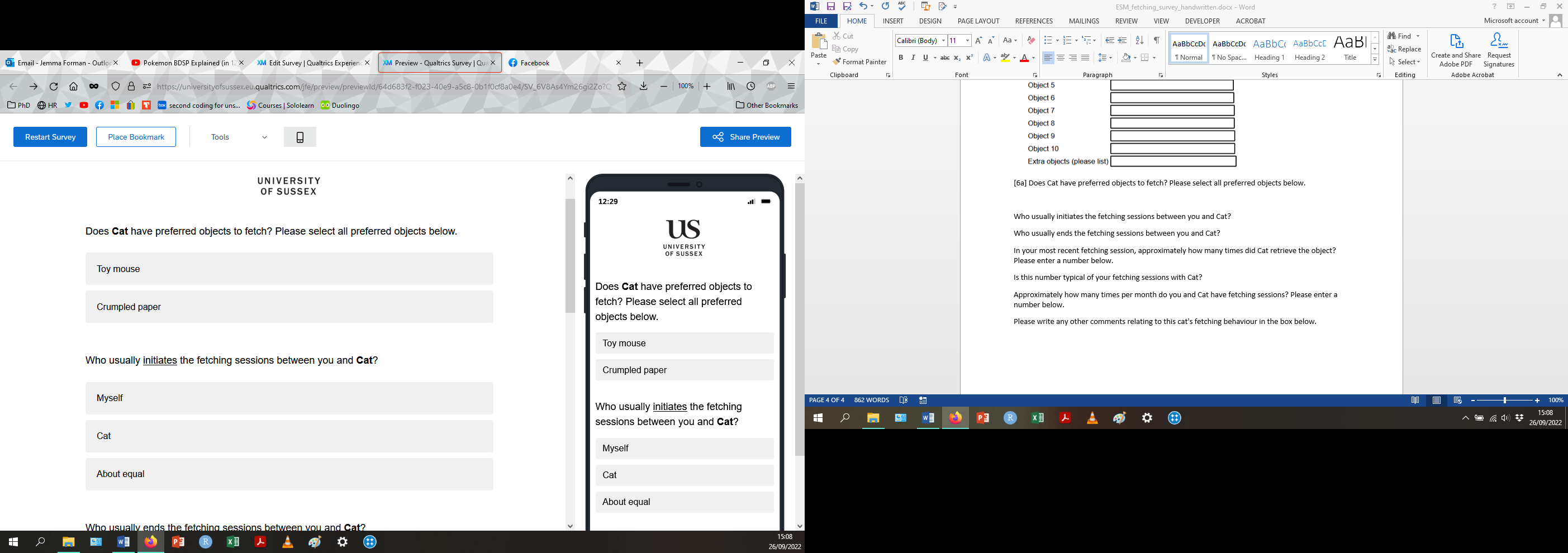


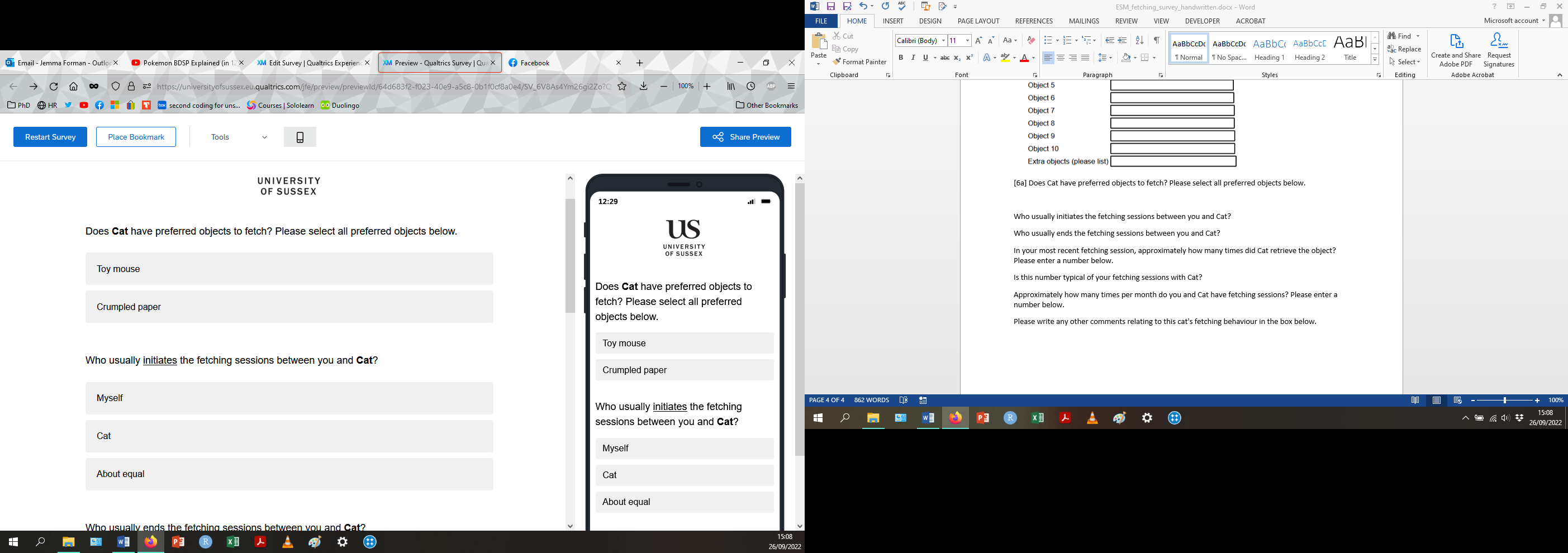
[8] Who usually ends the fetching sessions between you and **Cat**?

[9] In your most recent fetching session, approximately how many times did **Cat** retrieve the object?
 Please enter a number below.

[10] Is this number typical of your fetching sessions with **Cat**?


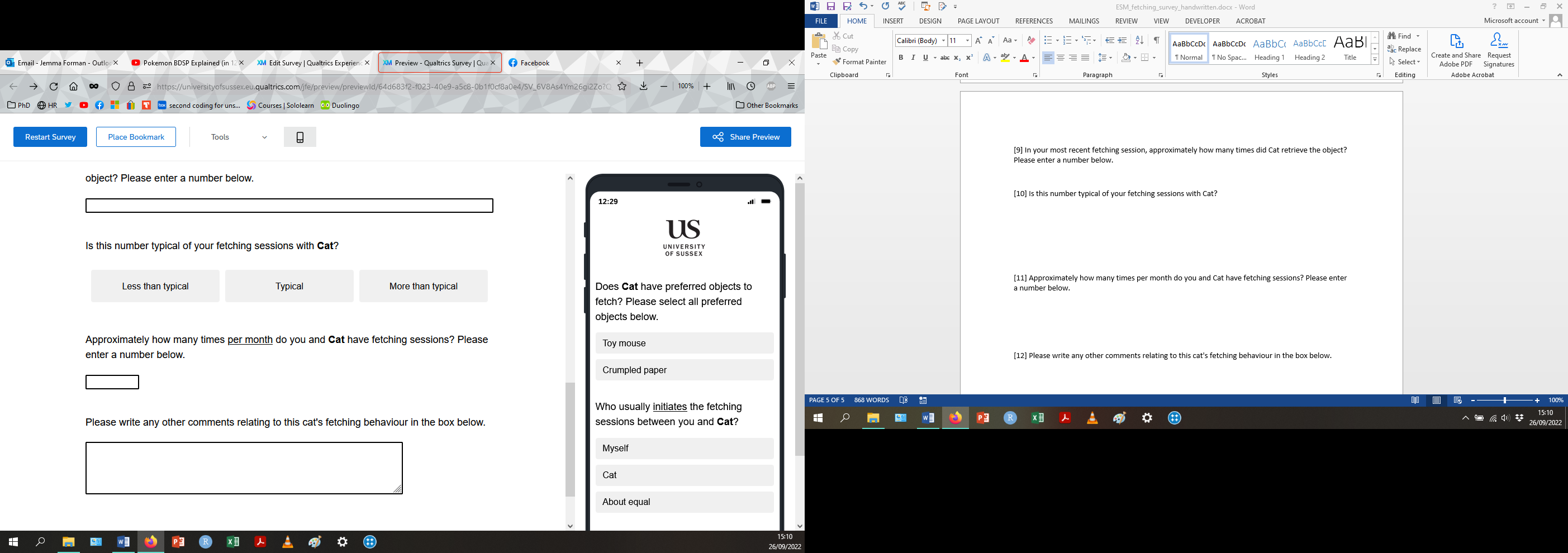


[11] Approximately how many times per month do you and **Cat** have fetching sessions?
 Please enter a number below.

[12] Please write any other comments relating to this cat's fetching behaviour in the box below.

This next set of questions will ask for demographic information about **Cat**.

[13] How old was **Cat** at the time you first noticed the fetching behaviour? (years and months)

Years

Months


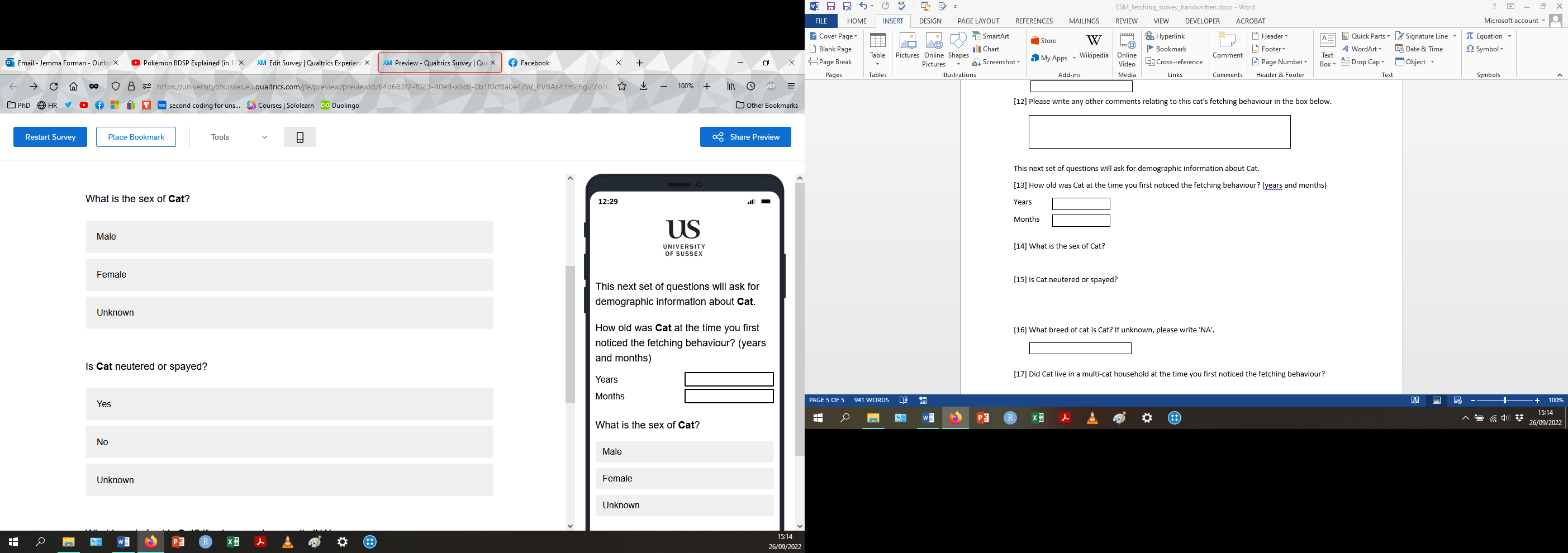
[14] What is the sex of **Cat**?


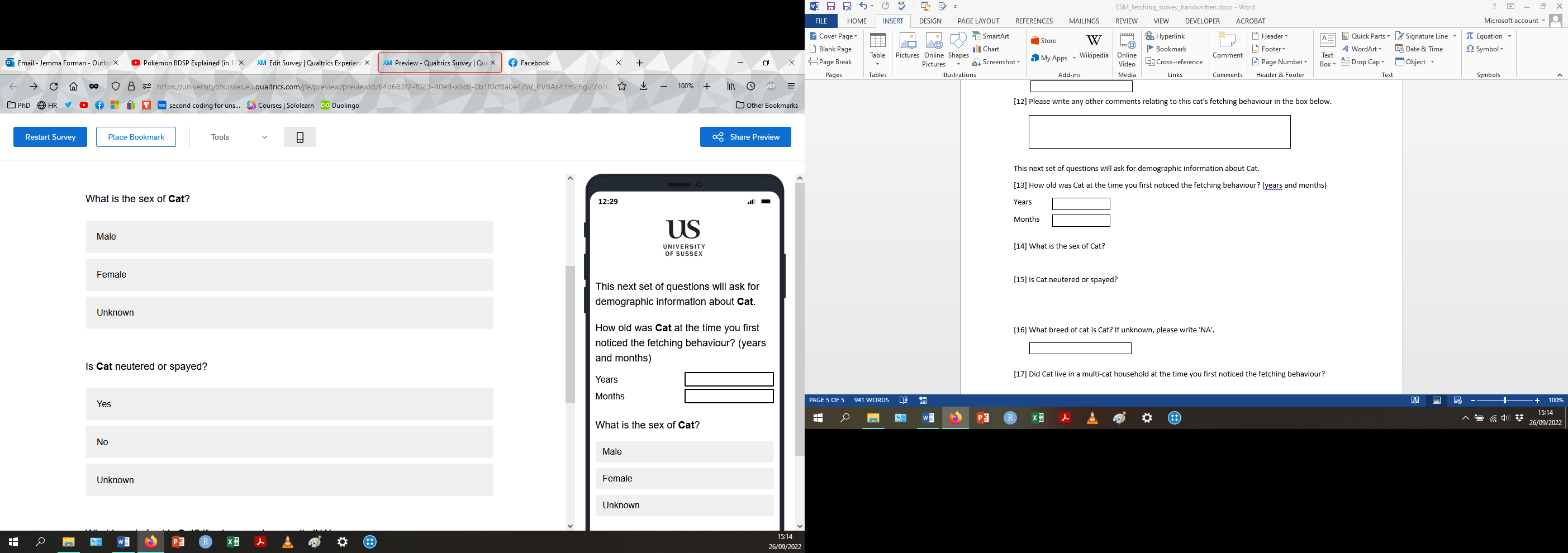
[15] Is **Cat** neutered or spayed?

[16] What breed of cat is **Cat**? If unknown, please write 'NA'.


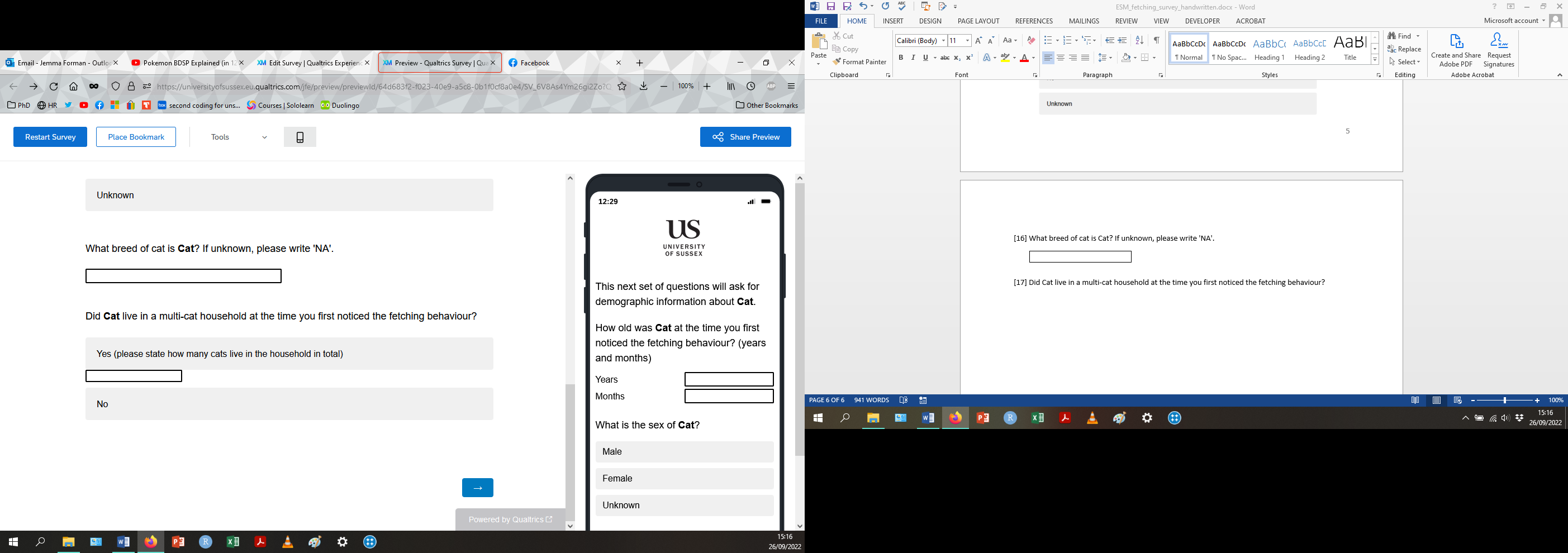
[17] Did **Cat** live in a multi-cat household at the time you first noticed the fetching behaviour?


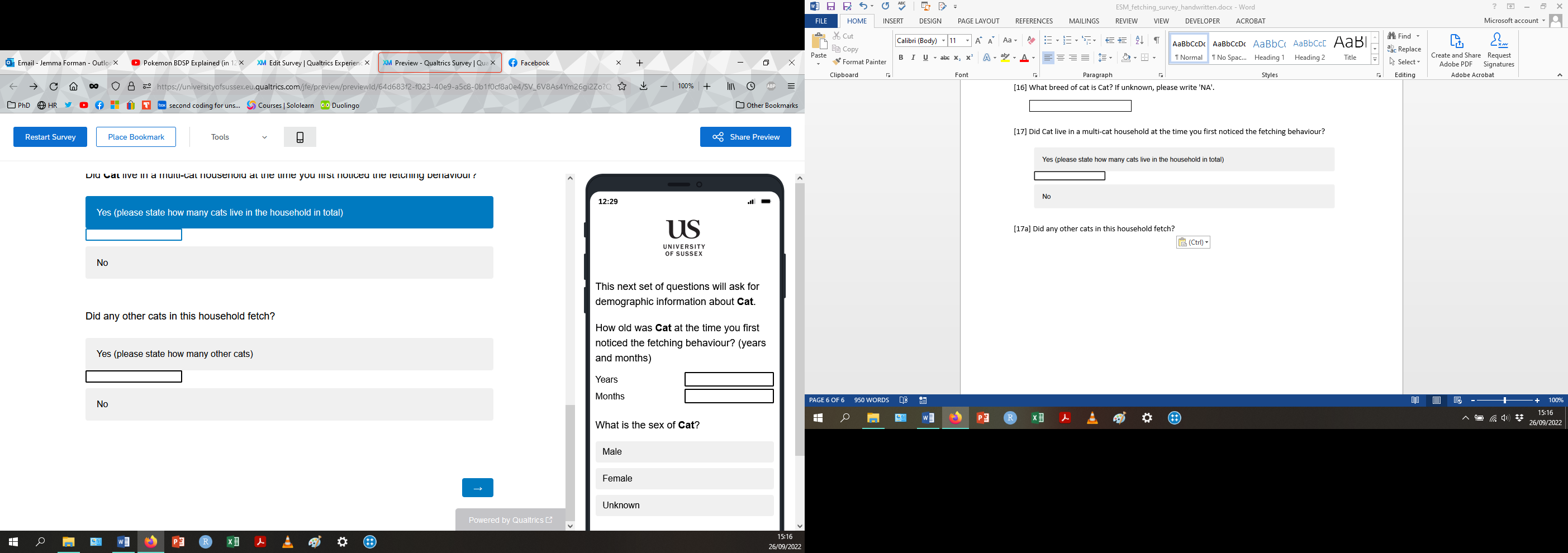
[17a] Did any other cats in this household fetch?

This next set of questions will ask for information about the people and other pet species in **Cat's** household.

[18] What country do you live in?

[19] How many people live in the household in total? Please enter a number below (including yourself)

This next set of questions will ask for information about yourself and other pet species in your current household.

**Person 1 refers to yourself.**


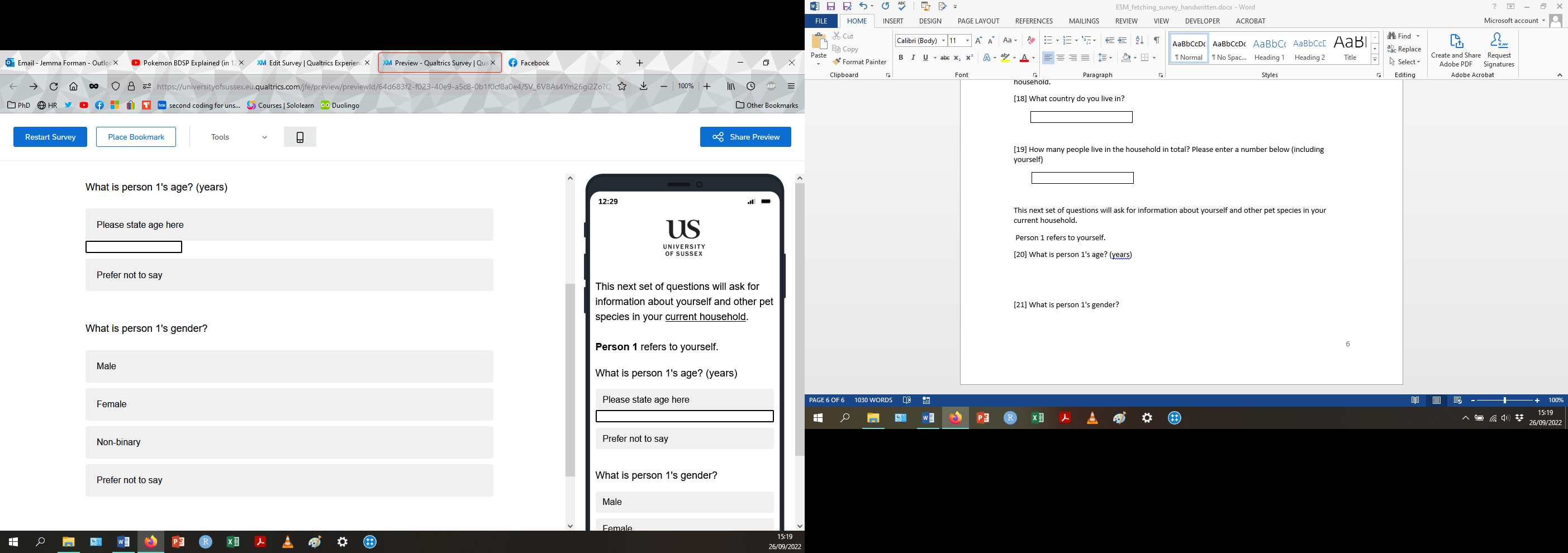
[20] What is person 1's age? (years)

[21] What is person 1's gender?


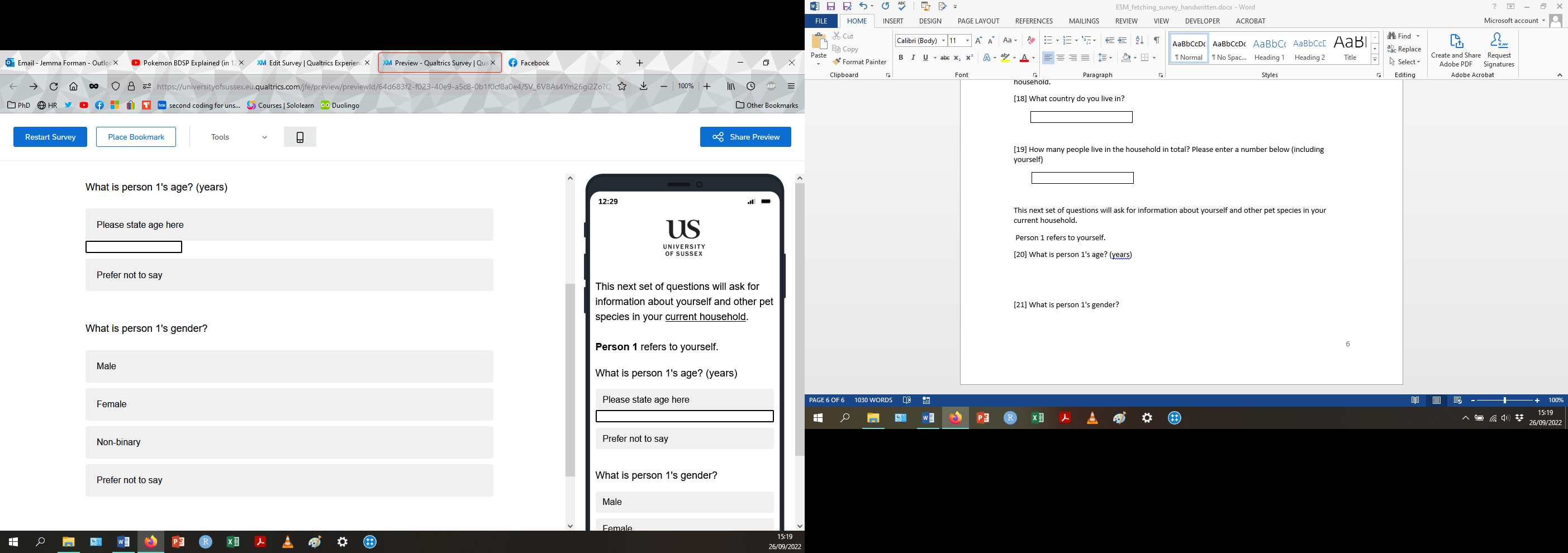


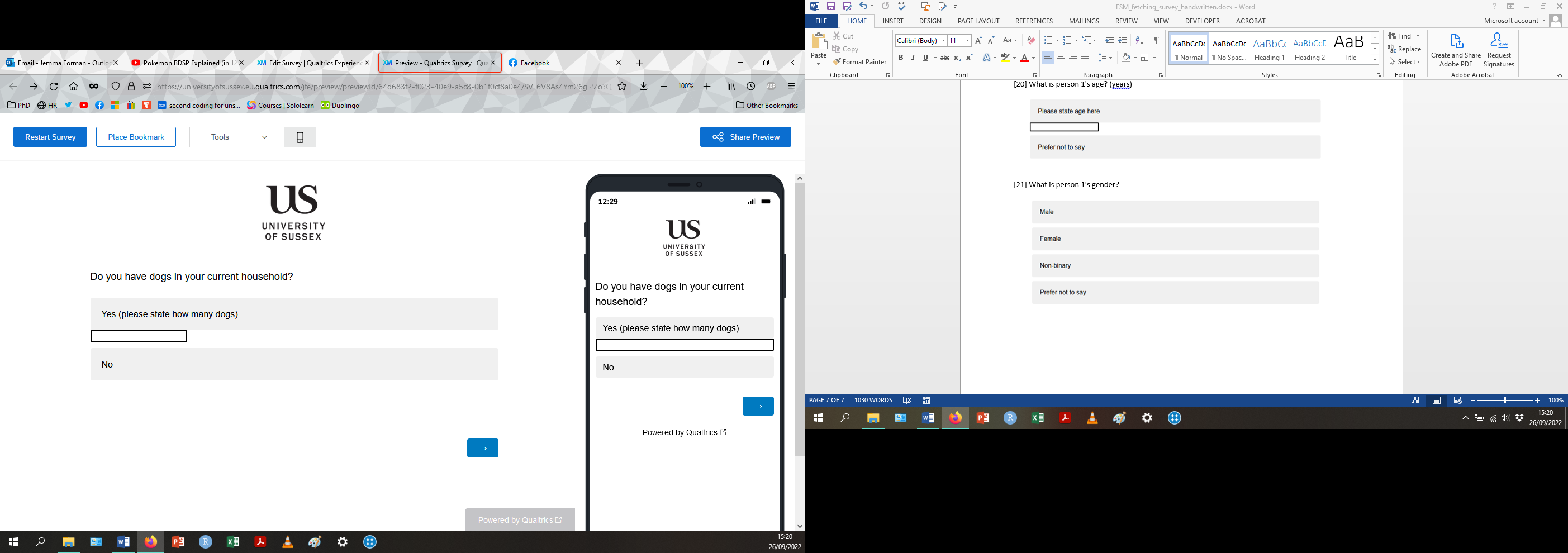
[22] Do you have dogs in your current household?

[22a] Do any of these dogs also fetch?


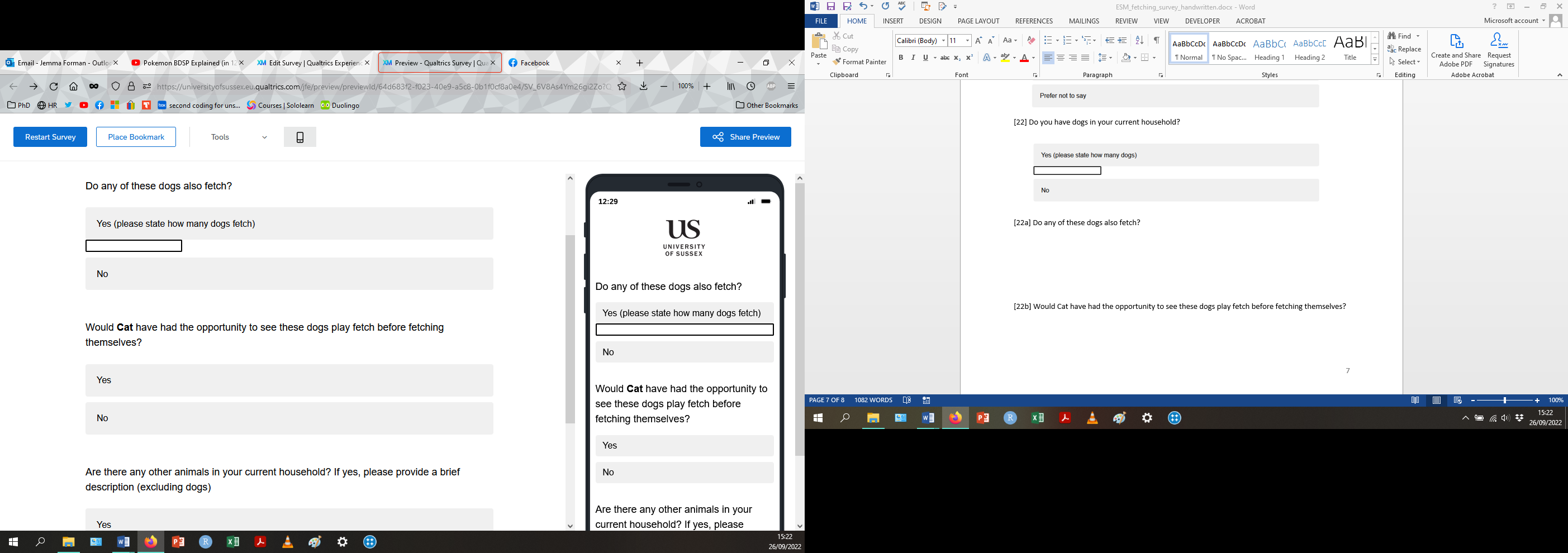


[22b] Would **Cat** have had the opportunity to see these dogs play fetch before fetching themselves?


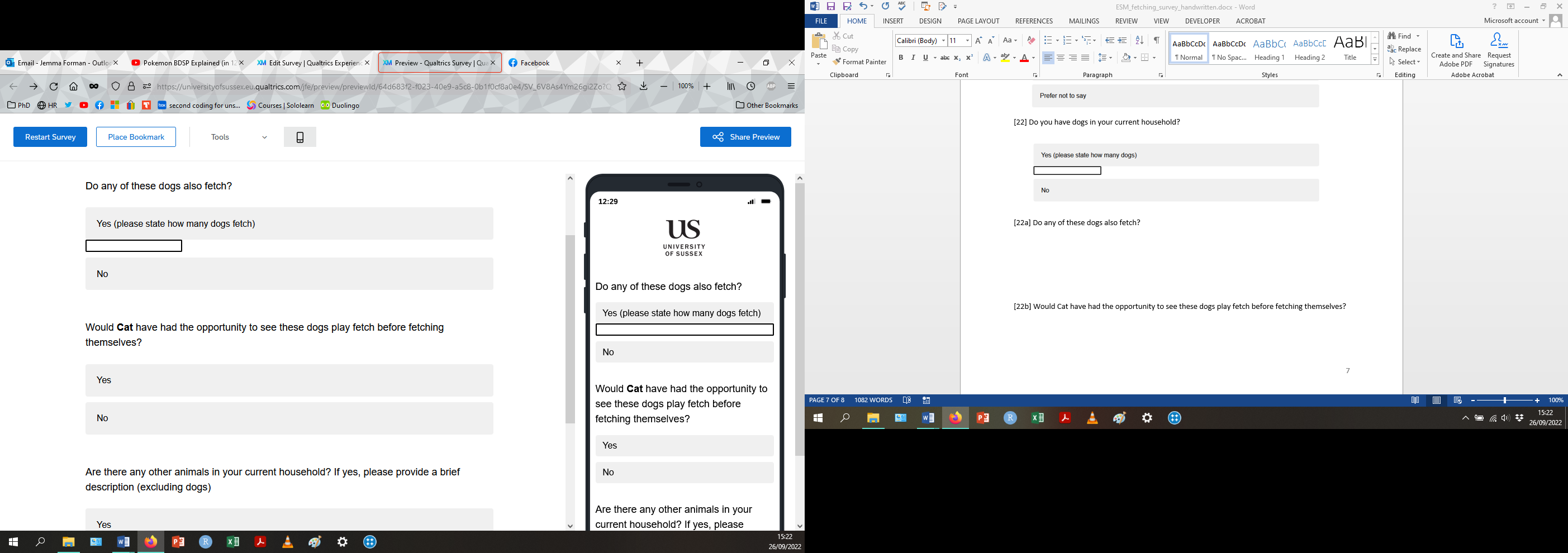


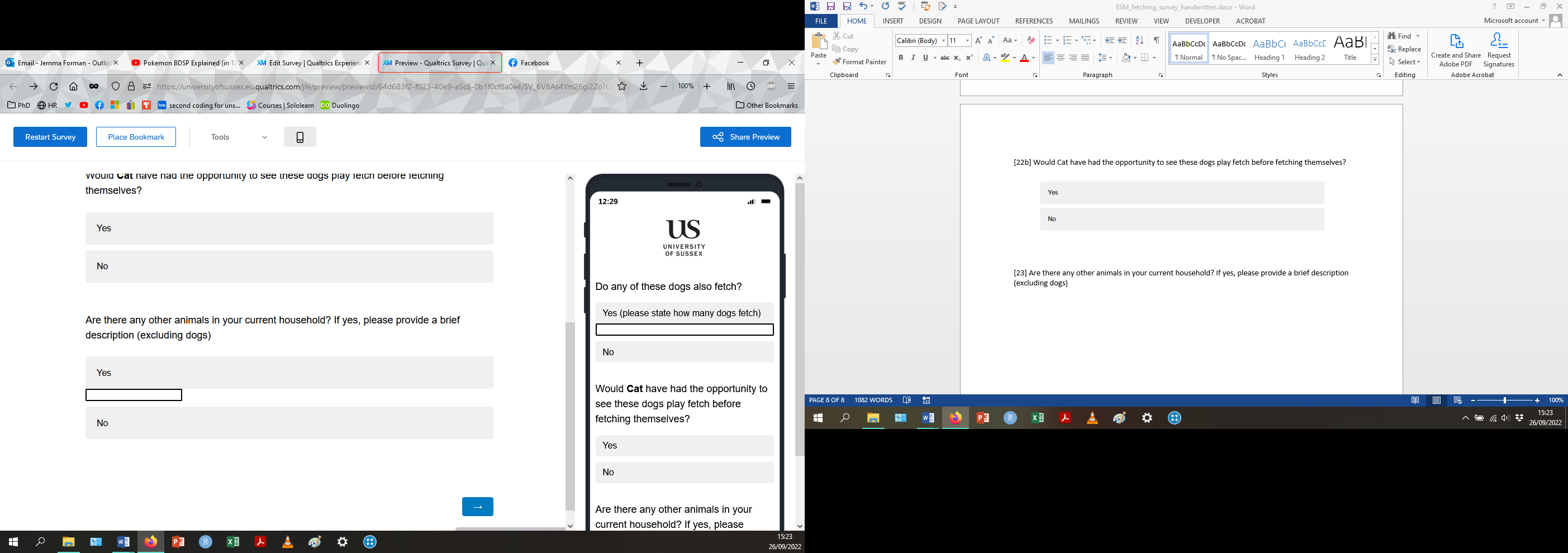
[23] Are there any other animals in your current household? If yes, please provide a brief description (excluding dogs)

Thank you for completing this questionnaire! Your responses have been successfully recorded and your time has been much appreciated.
