# Supplementary material for: Fetching felines: a survey of cat owners on the diversity of cat (Felis catus) fetching behaviour
Source: Sci Rep. 2023 Dec 14;13:20456. doi: 10.1038/s41598-023-47409-w (PMC10721921; doi:10.1038/s41598-023-47409-w)
Supplement: Supplementary file 2 — Supplementary Information 2. [file 41598_2023_47409_MOESM2_ESM.docx]

S2 Fetching Statistics

#required packages
packages = c("tidyverse", "gmodels", "car", "coin", "DescTools", "qqplotr", "sf", "rstatix", "flextable")
#will install and load the required packages above
package.check <- lapply(
 packages,
 FUN = function(x) {
 if (!require(x, character.only = TRUE)) {
 install.packages(x, dependencies = TRUE)
 library(x, character.only = TRUE)
 }
 }
)

#this is needed to bind multiple dataframes together
AppendMe <- function(dfNames) {
 do.call(rbind, lapply(dfNames, function(x) {
 cbind(get(x), source = x)
 }))
}

#blank theme for pie chart
blank_theme <- theme_minimal()+
 theme(
 axis.title.x = element_blank(),
 axis.title.y = element_blank(),
 panel.border = element_blank(),
 panel.grid=element_blank(),
 axis.ticks = element_blank(),
 plot.title=element_text(size=24, face="bold")
 )

#dataset
fetch_combined <- read_csv("S3_fetching_statistics_data.csv")

# 1. CAT DEMOGRAPHICS

## total cats in survey

#number of cats that fetched that people answered for
nrow(fetch_combined)

[1] 1154

## previous vs. current cat

#count of previous vs current cats
fetch_combined %>%
 group_by(current_previous) %>%
 summarise(n = n()) %>%
 mutate(freq = n / sum(n)) %>%
 mutate(Percentage=paste0(round(freq/sum(freq)*100,1),"%"))

# A tibble: 2 × 4
 current_previous n freq Percentage
 <chr> <int> <dbl> <chr>
1 Current cat 853 0.739 73.9%
2 Previous cat 301 0.261 26.1%

## trained vs. untrained

#count of how many cats were trained vs untrained
fetch_combined %>%
 group_by(trained) %>%
 summarise(n = n()) %>%
 mutate(freq = n / sum(n)) %>%
 mutate(Percentage=paste0(round(freq/sum(freq)*100,1),"%"))

# A tibble: 2 × 4
 trained n freq Percentage
 <chr> <int> <dbl> <chr>
1 Trained 65 0.0563 5.6%
2 Untrained 1089 0.944 94.4%

## sex x neuter status

#count of cat sex
fetch_combined %>%
 group_by(cat_sex, cat_neutered) %>%
 summarise(n = n(), .groups = "drop") %>%
 mutate(freq = n / sum(n)) %>%
 mutate(Percentage=paste0(round(freq/sum(freq)*100,1),"%"))

# A tibble: 5 × 5
 cat_sex cat_neutered n freq Percentage
 <chr> <chr> <int> <dbl> <chr>
1 Female No 36 0.0312 3.1%
2 Female Yes 501 0.434 43.4%
3 Male No 15 0.0130 1.3%
4 Male Unknown 1 0.000867 0.1%
5 Male Yes 601 0.521 52.1%

#person who didn't know neuter status (1 entry)
cat_neuter_excl <- fetch_combined %>%
 filter(cat_neutered != "Unknown")

cat_neuter_excl %>%
 group_by(cat_sex, cat_neutered) %>%
 summarise(n = n(), .groups = "drop") %>%
 mutate(freq = n / sum(n)) %>%
 mutate(Percentage=paste0(round(freq/sum(freq)*100,1),"%"))

# A tibble: 4 × 5
 cat_sex cat_neutered n freq Percentage
 <chr> <chr> <int> <dbl> <chr>
1 Female No 36 0.0312 3.1%
2 Female Yes 501 0.435 43.5%
3 Male No 15 0.0130 1.3%
4 Male Yes 601 0.521 52.1%

### cat sex

#count of cat sex
fetch_combined %>%
 group_by(cat_sex) %>%
 summarise(n = n()) %>%
 mutate(freq = n / sum(n)) %>%
 mutate(Percentage=paste0(round(freq/sum(freq)*100,1),"%"))

# A tibble: 2 × 4
 cat_sex n freq Percentage
 <chr> <int> <dbl> <chr>
1 Female 537 0.465 46.5%
2 Male 617 0.535 53.5%

### neuter status

#count of how many cats were neutered
fetch_combined %>%
 group_by(cat_neutered) %>%
 summarise(n = n()) %>%
 mutate(freq = n / sum(n)) %>%
 mutate(Percentage=paste0(round(freq/sum(freq)*100,1),"%"))

# A tibble: 3 × 4
 cat_neutered n freq Percentage
 <chr> <int> <dbl> <chr>
1 No 51 0.0442 4.4%
2 Unknown 1 0.000867 0.1%
3 Yes 1102 0.955 95.5%

#person who didn't know neuter status (1 entry)
cat_neuter_excl <- fetch_combined %>%
 filter(cat_neutered != "Unknown")

cat_neuter_excl %>%
 group_by(cat_neutered) %>%
 summarise(n = n()) %>%
 mutate(freq = n / sum(n)) %>%
 mutate(Percentage=paste0(round(freq/sum(freq)*100,1),"%"))

# A tibble: 2 × 4
 cat_neutered n freq Percentage
 <chr> <int> <dbl> <chr>
1 No 51 0.0442 4.4%
2 Yes 1102 0.956 95.6%

## cat breed

#count of cat breed
fetch_combined %>%
 group_by(cat_breed_grouped) %>%
 summarise(n = n()) %>%
 mutate(freq = n / sum(n)) %>%
 mutate(Percentage=paste0(round(freq/sum(freq)*100,1),"%")) %>%
 arrange(desc(n))

# A tibble: 30 × 4
 cat_breed_grouped n freq Percentage
 <chr> <int> <dbl> <chr>
 1 Unknown 501 0.434 43.4%
 2 Mixed 493 0.427 42.7%
 3 Siamese 36 0.0312 3.1%
 4 Bengal 16 0.0139 1.4%
 5 Ragdoll 12 0.0104 1%
 6 Maine Coon 11 0.00953 1%
 7 Nebelung 11 0.00953 1%
 8 Bombay 8 0.00693 0.7%
 9 Oriental 8 0.00693 0.7%
10 Russian Blue 8 0.00693 0.7%
# ℹ 20 more rows

#grouped cat breeds
fetch_combined %>%
 group_by(general_breed) %>%
 summarise(n = n()) %>%
 mutate(freq = n / sum(n)) %>%
 mutate(Percentage=paste0(round(freq/sum(freq)*100,1),"%")) %>%
 arrange(desc(n))

# A tibble: 3 × 4
 general_breed n freq Percentage
 <chr> <int> <dbl> <chr>
1 Unknown 501 0.434 43.4%
2 Mixed 493 0.427 42.7%
3 Purebred 160 0.139 13.9%

## continents and countries

#splitting into continents
countries_europe <- fetch_combined %>%
 filter(continent == "Europe")

countries_north_america <- fetch_combined %>%
 filter(continent == "North America")

countries_south_america <- fetch_combined %>%
 filter(continent == "South America")

countries_asia <- fetch_combined %>%
 filter(continent == "Asia")

countries_australasia <- fetch_combined %>%
 filter(continent == "Australasia")

countries_africa<- fetch_combined %>%
 filter(continent == "Africa")

### table of all continents

#overall continents
fetch_combined %>%
 group_by(continent) %>%
 summarise(n = n()) %>%
 mutate(freq = n / sum(n)) %>%
 mutate(Percentage=paste0(round(freq/sum(freq)*100,1),"%")) %>%
 arrange(desc(n))

# A tibble: 7 × 4
 continent n freq Percentage
 <chr> <int> <dbl> <chr>
1 North America 813 0.705 70.5%
2 Europe 265 0.230 23%
3 Australasia 34 0.0295 2.9%
4 South America 22 0.0191 1.9%
5 Asia 13 0.0113 1.1%
6 Africa 4 0.00347 0.3%
7 Multiple Countries 3 0.00260 0.3%

### tables of continent demographics

#count of European countries
countries_europe %>%
 group_by(country) %>%
 summarise(n = n()) %>%
 mutate(freq = n / sum(n)) %>%
 mutate(Percentage=paste0(round(freq/sum(freq)*100,1),"%")) %>%
 arrange(desc(n))

# A tibble: 20 × 4
 country n freq Percentage
 <chr> <int> <dbl> <chr>
 1 UK 160 0.604 60.4%
 2 Germany 22 0.0830 8.3%
 3 France 17 0.0642 6.4%
 4 Spain 16 0.0604 6%
 5 Netherlands 8 0.0302 3%
 6 Norway 6 0.0226 2.3%
 7 Belgium 4 0.0151 1.5%
 8 Greece 4 0.0151 1.5%
 9 Ireland 4 0.0151 1.5%
10 Italy 4 0.0151 1.5%
11 Portugal 3 0.0113 1.1%
12 Russia 3 0.0113 1.1%
13 Switzerland 3 0.0113 1.1%
14 Austria 2 0.00755 0.8%
15 Finland 2 0.00755 0.8%
16 Iceland 2 0.00755 0.8%
17 Romania 2 0.00755 0.8%
18 Latvia 1 0.00377 0.4%
19 Poland 1 0.00377 0.4%
20 Sweden 1 0.00377 0.4%

#count of Asian countries
countries_asia %>%
 group_by(country) %>%
 summarise(n = n()) %>%
 mutate(freq = n / sum(n)) %>%
 mutate(Percentage=paste0(round(freq/sum(freq)*100,1),"%")) %>%
 arrange(desc(n))

# A tibble: 7 × 4
 country n freq Percentage
 <chr> <int> <dbl> <chr>
1 Turkey 4 0.308 30.8%
2 Japan 2 0.154 15.4%
3 Malaysia 2 0.154 15.4%
4 Singapore 2 0.154 15.4%
5 Indonesia 1 0.0769 7.7%
6 Israel 1 0.0769 7.7%
7 Pakistan 1 0.0769 7.7%

#count of African countries
#only South Africa
countries_africa %>%
 group_by(country) %>%
 summarise(n = n()) %>%
 mutate(freq = n / sum(n)) %>%
 mutate(Percentage=paste0(round(freq/sum(freq)*100,1),"%")) %>%
 arrange(desc(n))

# A tibble: 1 × 4
 country n freq Percentage
 <chr> <int> <dbl> <chr>
1 South Africa 4 1 100%

#count of North American countries
countries_north_america %>%
 group_by(country) %>%
 summarise(n = n()) %>%
 mutate(freq = n / sum(n)) %>%
 mutate(Percentage=paste0(round(freq/sum(freq)*100,1),"%")) %>%
 arrange(desc(n))

# A tibble: 5 × 4
 country n freq Percentage
 <chr> <int> <dbl> <chr>
1 USA 724 0.891 89.1%
2 Canada 79 0.0972 9.7%
3 Mexico 7 0.00861 0.9%
4 Guatemala 2 0.00246 0.2%
5 El Salvador 1 0.00123 0.1%

#count of South American countries
countries_south_america %>%
 group_by(country) %>%
 summarise(n = n()) %>%
 mutate(freq = n / sum(n)) %>%
 mutate(Percentage=paste0(round(freq/sum(freq)*100,1),"%")) %>%
 arrange(desc(n))

# A tibble: 5 × 4
 country n freq Percentage
 <chr> <int> <dbl> <chr>
1 Brazil 14 0.636 63.6%
2 Colombia 3 0.136 13.6%
3 Argentina 2 0.0909 9.1%
4 Chile 2 0.0909 9.1%
5 Venezuela 1 0.0455 4.5%

#count of Australasian countries
countries_australasia %>%
 group_by(country) %>%
 summarise(n = n()) %>%
 mutate(freq = n / sum(n)) %>%
 mutate(Percentage=paste0(round(freq/sum(freq)*100,1),"%")) %>%
 arrange(desc(n))

# A tibble: 2 × 4
 country n freq Percentage
 <chr> <int> <dbl> <chr>
1 Australia 24 0.706 70.6%
2 New Zealand 10 0.294 29.4%

#all country percentages
count_countries_map <- fetch_combined %>%
 group_by(country) %>%
 summarise(n = n()) %>%
 mutate(freq = n / sum(n)) %>%
 mutate(Percentage=paste0(round(freq/sum(freq)*100,1),"%")) %>%
 arrange(desc(n))

count_countries_map <- rename(count_countries_map, region = country)

## favourite object to fetch

### table

count_fave_obj <- fetch_combined %>%
 group_by(fave_object_group) %>%
 summarise(n = n()) %>%
 mutate(freq = n / sum(n)) %>%
 mutate(Percentage=paste0(round(freq/sum(freq)*100,1),"%")) %>%
 arrange(desc(n))
count_fave_obj

# A tibble: 14 × 4
 fave_object_group n freq Percentage
 <chr> <int> <dbl> <chr>
 1 Toys 443 0.384 38.4%
 2 Spherical objects 292 0.253 25.3%
 3 Cosmetics 110 0.0953 9.5%
 4 Miscellaneous 92 0.0797 8%
 5 Arts and Crafts 51 0.0442 4.4%
 6 Bottle parts 48 0.0416 4.2%
 7 Stationery 25 0.0217 2.2%
 8 Springs 22 0.0191 1.9%
 9 Twist ties 20 0.0173 1.7%
10 String/String-like objects 15 0.0130 1.3%
11 Clothing items 13 0.0113 1.1%
12 Consumables 9 0.00780 0.8%
13 Ring-shaped objects 7 0.00607 0.6%
14 Scraps 7 0.00607 0.6%

#barplot for favourite object
fave_object_barplot <- ggplot(data=count_fave_obj, aes(x=fave_object_group, y=n, fill = fave_object_group)) +
 geom_bar(stat="identity", color = "black", width = .8) +
 labs(x = "Favourite category of object to fetch\n", y = "\nFrequency") +
 scale_x_discrete(limits=c("Scraps", "Ring-shaped objects", "Consumables", "Clothing items", "String/String-like objects", "Twist ties", "Springs", "Stationery", "Bottle parts", "Arts and Crafts", "Miscellaneous", "Cosmetics", "Spherical objects", "Toys")) +
 geom_text(aes(label=Percentage), hjust=-.1, color="black", size=6.5) +
 scale_y_continuous(expand = c(0,0), limits=c(0,520)) +
 scale_fill_manual(values=c("#FEFE62", "#44AA99", "#DC3220", "#117733","#88CCEE","#D41159", "#DDCC77", "#6F5458", "#AA4499", "#994F00","#005AB5", "#332288", "#FFC107", "#E66100")) +
 coord_flip() +
 theme_classic() +
 theme(text = element_text(family = "Calibri")) +
 theme(axis.text.x = element_text(color = "black", size = 18)) +
 theme(axis.text.y = element_text(color = "black", size = 18)) +
 theme(axis.title.x = element_text(color= "black", face="bold", size = 20)) +
 theme(axis.title.y = element_text(color= "black", face="bold", size = 20)) +
 theme(legend.position="none") +
 theme(plot.caption = element_text(face = "italic", size = 14, hjust = -.45))

### barplot

#doesn't look good in R but looks good when knitted to Word
fave_object_barplot


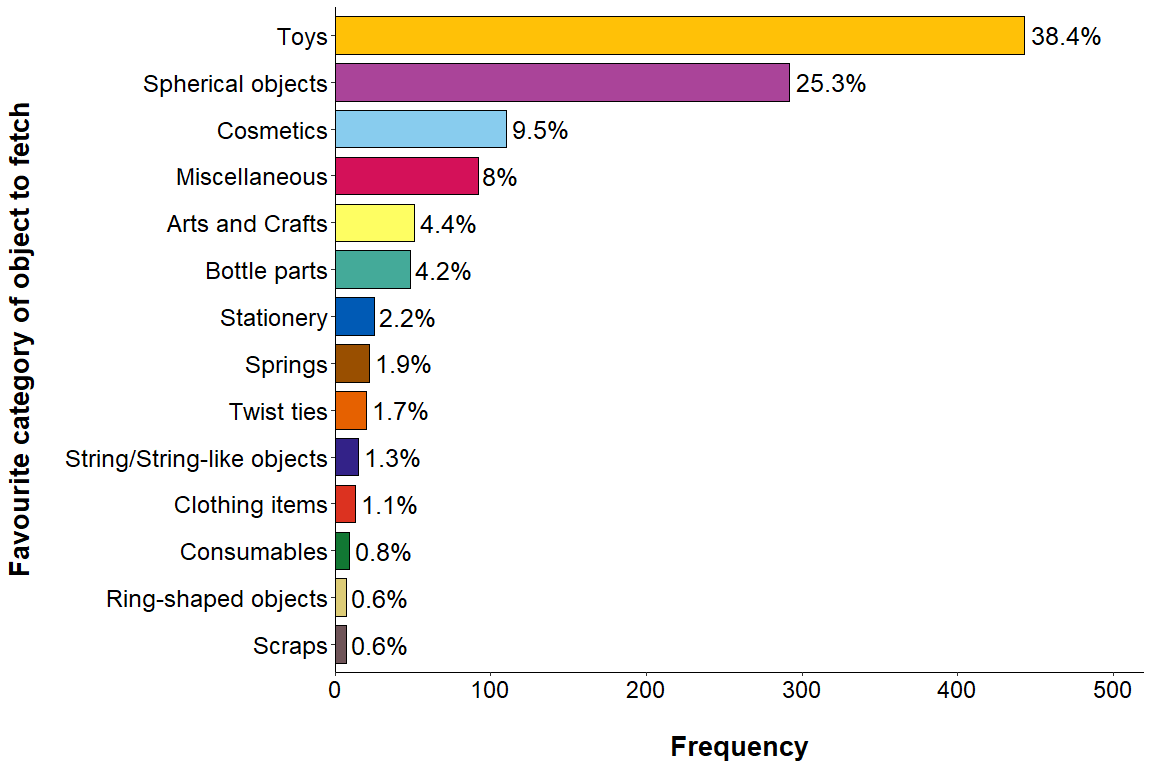


## cat age when first noticed fetching

#cat age when first noticed fetching (in months)
fetch_combined %>%
 summarise(mean = mean(fetch_age_months_total), sd = sd(fetch_age_months_total), min = min(fetch_age_months_total), max = max(fetch_age_months_total), median = median(fetch_age_months_total))

# A tibble: 1 × 5
 mean sd min max median
 <dbl> <dbl> <dbl> <dbl> <dbl>
1 15.9 23.5 0 204 7

quantile(fetch_combined$fetch_age_months_total)

0% 25% 50% 75% 100%
 0 5 7 16 204

- excluding those who put “0” and “NA” as their answer

#excluding cats who had '0' and 'NA' for this answer (12 cats)
cat_fetch_age_excl <- fetch_combined %>%
 filter(fetch_age_months_total != "0")

cat_fetch_age_excl %>%
 summarise(mean = mean(fetch_age_months_total), sd = sd(fetch_age_months_total), min = min(fetch_age_months_total), max = max(fetch_age_months_total), median = median(fetch_age_months_total))

# A tibble: 1 × 5
 mean sd min max median
 <dbl> <dbl> <dbl> <dbl> <dbl>
1 16.1 23.6 1 204 7

- excludes people who put “0” months for when they first noticed the fetching behaviour. So the cat may have already been fetching prior to adoption or started fetching as soon as they were adopted.

### histogram of cats ages

#12 cases of 0s
#histogram
p <- ggplot(data=fetch_combined, aes(x = fetch_age_years_total)) +
 stat_bin(breaks = c(0,1,2,3,4,5,6,7,8,9,10,17), fill = "#4a536b", color = "black") +
 stat_bin(breaks = c(0,1,2,3,4,5,6,7,8,9,10,11,12,13,14,15,16,17), geom='text', color='black', aes(label=..count..), vjust = -1.1) +
 scale_x_continuous(breaks = seq(0,17,2)) +
 scale_y_continuous(breaks = seq(0, 1000, 200)) +
 coord_cartesian(clip="off",ylim =c(0,1000)) +
 xlab("\nAge of cat when \nfetching first noticed (years)") + ylab("\nFrequency")+
 theme_bw()+
 ggtitle("c") +
 theme(plot.title = element_text(hjust = -.22, size = 20, face = "bold")) +
 theme(legend.position = "None") +
 theme(axis.text.x = element_text(color = "black", size = 14)) +
 theme(axis.text.y = element_text(color = "black", size = 14)) +
 theme(axis.title.x = element_text(color= "black", face="bold", size = 14)) +
 theme(axis.title.y = element_text(color= "black", face="bold", size = 14)) +
 theme(axis.title.y = element_text(margin = margin(t = 0, r = 20, b = 0, l = 0)))
p


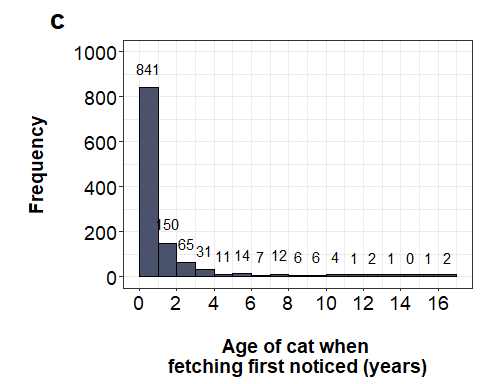


## cat life stage when first noticed fetching

- using these guidelines <https://journals.sagepub.com/doi/pdf/10.1177/1098612X21993657>

### table

#cats with "0" as their answer for their age are categorised as "Kittens"
fetch_combined %>%
 group_by(life_stage) %>%
 summarise(n = n()) %>%
 mutate(freq = n / sum(n)) %>%
 mutate(Percentage=paste0(round(freq/sum(freq)*100,1),"%")) %>%
 arrange(desc(n))

# A tibble: 4 × 4
 life_stage n freq Percentage
 <chr> <int> <dbl> <chr>
1 Kitten 701 0.607 60.7%
2 Young adult 415 0.360 36%
3 Mature adult 22 0.0191 1.9%
4 Senior 16 0.0139 1.4%

## duration of time cat had fetched for at time of survey

#use fetch_length_months_total variable
fetch_combined$fetch_length_months_total <- as.numeric(fetch_combined$fetch_length_months_total)
m_length_total <- round(mean(fetch_combined$fetch_length_months_total, na.rm = TRUE),2)
sd_length_total <- round(sd(fetch_combined$fetch_length_months_total, na.rm = TRUE),2)
range_length_total <- round(range(fetch_combined$fetch_length_months_total, na.rm = TRUE),2)

#excluding "0" cases
length_fetching_excl <- fetch_combined %>%
 filter(fetch_length_months_total != "0")

length_fetching_excl$fetch_length_months_total <- as.numeric(length_fetching_excl$fetch_length_months_total)
m_length_total_excl <- round(mean(length_fetching_excl$fetch_length_months_total, na.rm = TRUE),2)
sd_length_total_excl <- round(sd(length_fetching_excl$fetch_length_months_total, na.rm = TRUE),2)
range_length_total_excl <- round(range(length_fetching_excl$fetch_length_months_total, na.rm = TRUE),2)

### table

#duration of fetching
duration_of_fetching <- tibble::tibble(" " = c("mean", "SD", "min range", "max range"), duration_of_fetching_months = c(m_length_total,sd_length_total,range_length_total), duration_of_fetching_months_excl = c(m_length_total_excl,sd_length_total_excl,range_length_total_excl))

flextable(duration_of_fetching)

|  | duration_of_fetching_months | duration_of_fetching_months_excl |
| --- | --- | --- |
| mean | 51.53 | 52.12 |
| SD | 48.72 | 48.68 |
| min range | 0.00 | 1.00 |
| max range | 244.00 | 244.00 |

## initiated fetch sessions

### table

#count of who initiated fetch sessions on average
count_initiate_fetch <- fetch_combined %>%
 group_by(initiate_fetch) %>%
 summarise(n = n()) %>%
 mutate(freq = n / sum(n)) %>%
 mutate(Percentage=paste0(round(freq/sum(freq)*100,1),"%")) %>%
 arrange(desc(n))

count_initiate_fetch

# A tibble: 3 × 4
 initiate_fetch n freq Percentage
 <chr> <int> <dbl> <chr>
1 Cat 553 0.479 47.9%
2 About equal 352 0.305 30.5%
3 Myself 249 0.216 21.6%

#making the pie chart
#data wrangling
count_initiate_fetch <- count_initiate_fetch %>%
 select(-freq)

count_initiate_fetch <- rename(count_initiate_fetch, freq = n)

#pie chart
count_initiate_fetch_pie <- count_initiate_fetch %>%
 arrange(desc(initiate_fetch)) %>%
 mutate(labels_text = cumsum(freq) - freq/2)

#convert into factors in the correct order:

count_initiate_fetch_pie$Percentage <- factor(count_initiate_fetch_pie$Percentage, levels = unique(count_initiate_fetch_pie$Percentage))

count_initiate_fetch_pie$initiate_fetch <- factor(count_initiate_fetch_pie$initiate_fetch, levels = unique(count_initiate_fetch_pie$initiate_fetch))

pie <- count_initiate_fetch_pie %>%
 ggplot(aes(x="", y=freq, fill = initiate_fetch)) +
 geom_bar(width = 1, stat = "identity") +
 coord_polar(theta = "y") +
 geom_text(aes(label = Percentage),
 position = position_stack(vjust = 0.5), size = 10) +
 scale_fill_manual(
 values=c("#e2725b", "#3f826d", "#e1e6e1"),
 name = "", labels = c("Owner", "Cat", "About Equal")) +
 theme(text = element_text(family = "Calibri"))

pie_initiate <- pie + blank_theme +
labs(x = "", y = "", title = "a") +
 theme(plot.title = element_text(vjust = -8, hjust = 0, size = 40),
 legend.title = element_text(hjust = 0.5, face="bold", size = 26)) +
 theme(axis.text.x=element_blank()) +
 theme(legend.text=element_text(size=24))

### pie chart

pie_initiate


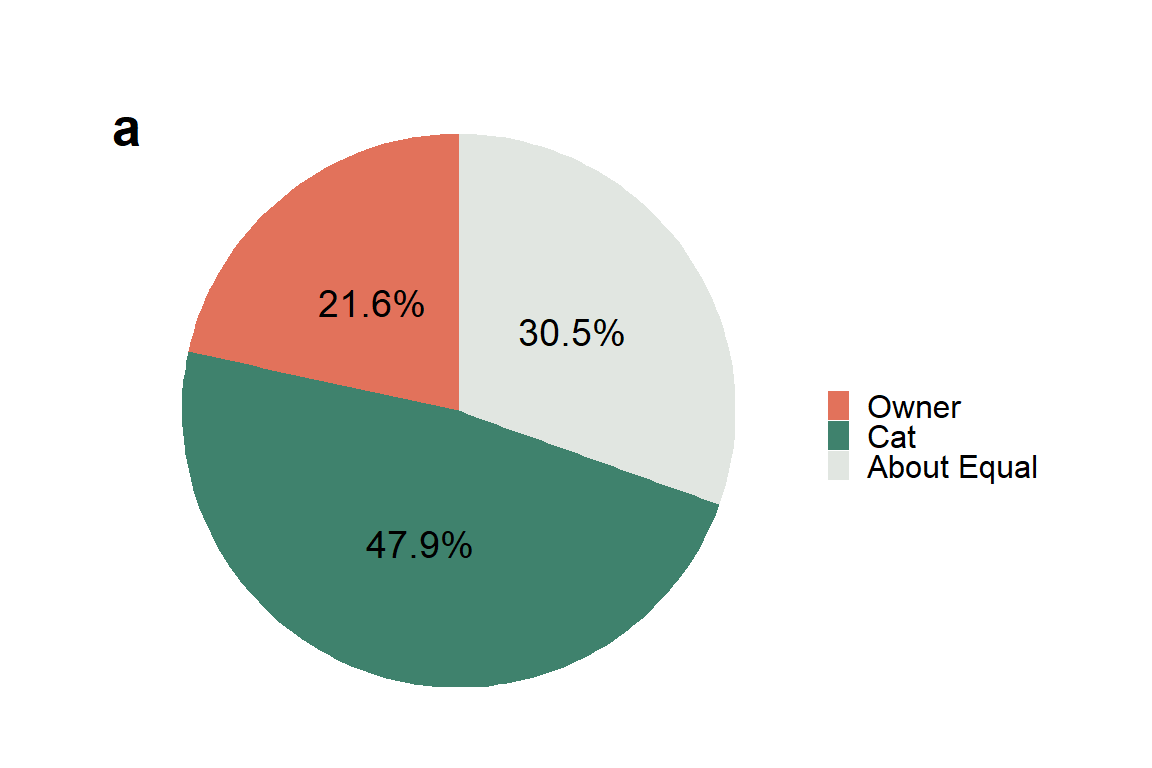


## ended fetch sessions

### table

#count of who ended fetch sessions on average
count_end_fetch <- fetch_combined %>%
 group_by(end_fetch) %>%
 summarise(n = n()) %>%
 mutate(freq = n / sum(n)) %>%
 mutate(Percentage=paste0(round(freq/sum(freq)*100,1),"%")) %>%
 arrange(desc(n))

count_end_fetch

# A tibble: 3 × 4
 end_fetch n freq Percentage
 <chr> <int> <dbl> <chr>
1 Cat 675 0.585 58.5%
2 Myself 248 0.215 21.5%
3 About equal 231 0.200 20%

#making the pie chart
#data wrangling
count_end_fetch <- count_end_fetch %>%
 select(-freq)

count_end_fetch <- rename(count_end_fetch, freq = n)

#pie chart
count_end_fetch_pie <- count_end_fetch %>%
 arrange(desc(end_fetch)) %>%
 mutate(labels_text = cumsum(freq) - freq/2)

#convert into factors in the correct order:

count_end_fetch_pie$Percentage <- factor(count_end_fetch_pie$Percentage, levels = unique(count_end_fetch_pie$Percentage))

count_end_fetch_pie$end_fetch <- factor(count_end_fetch_pie$end_fetch, levels = unique(count_end_fetch_pie$end_fetch))

pie <- count_end_fetch_pie %>%
 ggplot(aes(x="", y=freq, fill = end_fetch)) +
 geom_bar(width = 1, stat = "identity") +
 coord_polar(theta = "y") +
 geom_text(aes(label = Percentage),
 position = position_stack(vjust = 0.5), size = 10) +
 scale_fill_manual(
 values=c("#e2725b", "#3f826d", "#e1e6e1"),
 name = "", labels = c("Owner", "Cat", "About Equal")) +
 theme(text = element_text(family = "Calibri"))

pie_end <- pie + blank_theme +
 labs(x = "", y = "", title = "b") +
 theme(plot.title = element_text(vjust = -8, hjust = 0, size = 40),
 legend.title = element_text(hjust = 0.5, face="bold", size = 26)) +
 theme(axis.text.x=element_blank()) +
 theme(legend.text=element_text(size=24))

### pie chart

pie_end


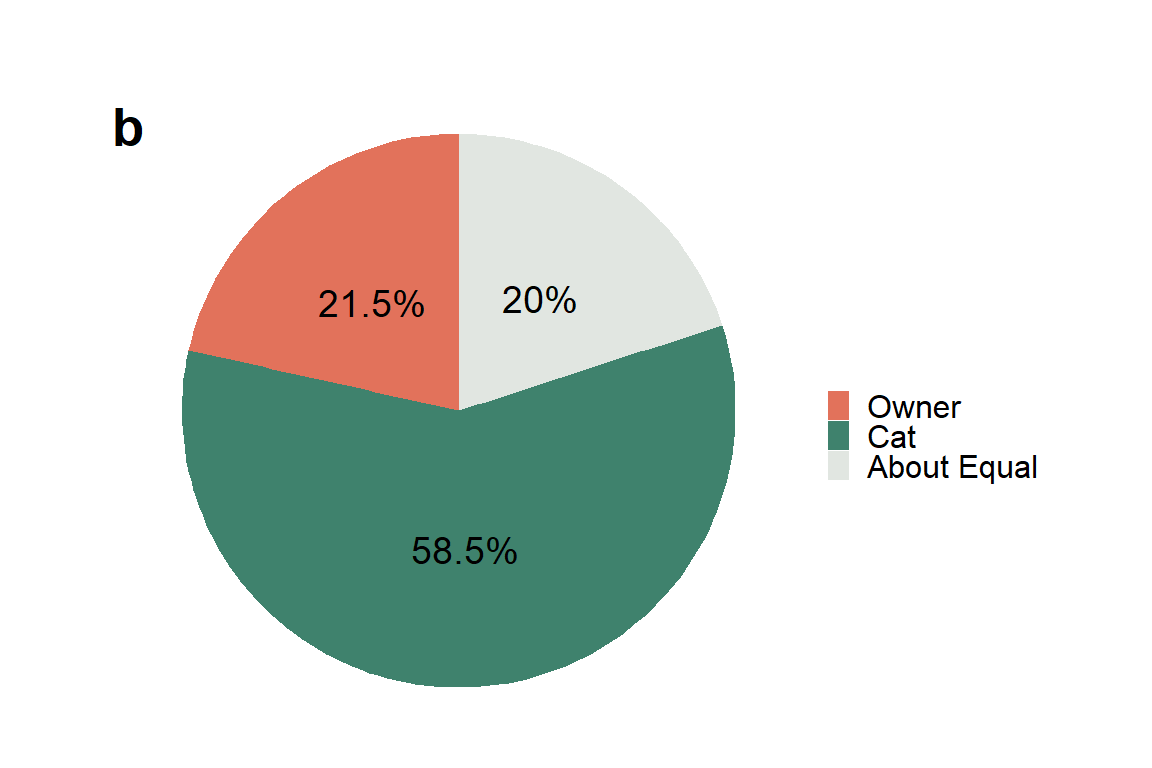


## no. times object retrieved in most recent fetching session

### raw counts

#count of the number of times an object was retrieved in the most recent fetching session
fetch_combined %>%
 group_by(times_object_retrieved_recent) %>%
 summarise(n = n()) %>%
 mutate(freq = n / sum(n)) %>%
 mutate(Percentage=paste0(round(freq/sum(freq)*100,1),"%")) %>%
 arrange(desc(n))

# A tibble: 24 × 4
 times_object_retrieved_recent n freq Percentage
 <dbl> <int> <dbl> <chr>
 1 5 199 0.172 17.2%
 2 3 187 0.162 16.2%
 3 4 151 0.131 13.1%
 4 10 141 0.122 12.2%
 5 6 103 0.0893 8.9%
 6 2 74 0.0641 6.4%
 7 8 59 0.0511 5.1%
 8 7 57 0.0494 4.9%
 9 15 53 0.0459 4.6%
10 20 35 0.0303 3%
# ℹ 14 more rows

### table of ranges

#ranges of recent fetch sessions
#relevelling "6-10" after "1-5" to be in order
fetch_combined$fetch_recent_range <- as.factor(fetch_combined$fetch_recent_range)

fetch_combined$fetch_recent_range <- fct_relevel(fetch_combined$fetch_recent_range, "6-10", after = 2)

fetch_combined %>%
 group_by(fetch_recent_range) %>%
 summarise(n = n()) %>%
 mutate(freq = n / sum(n)) %>%
 mutate(Percentage=paste0(round(freq/sum(freq)*100,1),"%")) %>%
 arrange(fetch_recent_range)

# A tibble: 8 × 4
 fetch_recent_range n freq Percentage
 <fct> <int> <dbl> <chr>
1 0 4 0.00347 0.3%
2 1-5 635 0.550 55%
3 6-10 371 0.321 32.1%
4 11-15 86 0.0745 7.5%
5 16-20 35 0.0303 3%
6 21-25 9 0.00780 0.8%
7 26-30 10 0.00867 0.9%
8 31+ 4 0.00347 0.3%

### histogram

p <- ggplot(data=fetch_combined, aes(x = times_object_retrieved_recent)) +
 stat_bin(breaks = c(0, 5, 10, 15, 20, 25, 30, 35, 40, 45, 50, 55, 60, 65, 70), fill = "#aed6dc", color = "black") +
 stat_bin(breaks = c(0, 5, 10, 15, 20, 25, 30, 35, 40, 45, 50, 55, 60, 65, 70), geom='text', color='black', aes(label=..count..), vjust = -1.1) +
 #scale_x_continuous(breaks = c(0, 5, 10, 15, 20, 25, 30, 35, 40, 45, 50, 55, 60, 65, 70)) +
 scale_x_continuous(breaks = seq(0,70,10)) +
 scale_y_continuous(breaks = seq(0, 700, 100)) +
 coord_cartesian(clip="off",ylim =c(0,700)) +
 xlab("\nNumber of retrievals in the \nmost recent fetching session") + ylab("\nFrequency")+
 theme_bw()+
 ggtitle("a") +
 theme(plot.title = element_text(hjust = -.22, size = 20, face = "bold")) +
 theme(legend.position = "None") +
 theme(axis.text.x = element_text(color = "black", size = 14)) +
 theme(axis.text.y = element_text(color = "black", size = 14)) +
 theme(axis.title.x = element_text(color= "black", face="bold", size = 14)) +
 theme(axis.title.y = element_text(color= "black", face="bold", size = 14)) +
 theme(axis.title.y = element_text(margin = margin(t = 0, r = 20, b = 0, l = 0)))
p


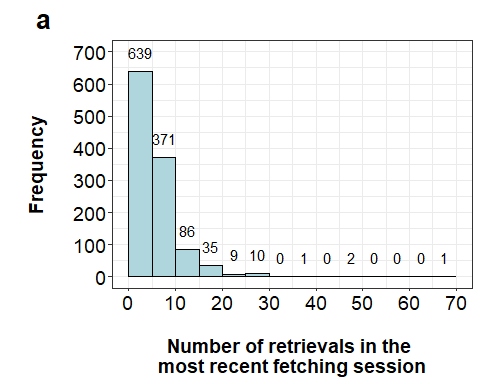


## no. monthly fetching sessions

### raw counts

#count of the number of fetching sessions per month
#NOTE: there were no responses between 61 - 70
fetch_combined$times_monthly_fetch_sessions <- as.numeric(fetch_combined$times_monthly_fetch_sessions)

fetch_combined %>%
 group_by(times_monthly_fetch_sessions) %>%
 summarise(n = n()) %>%
 mutate(freq = n / sum(n)) %>%
 mutate(Percentage=paste0(round(freq/sum(freq)*100,1),"%")) %>%
 arrange(desc(n))

# A tibble: 38 × 4
 times_monthly_fetch_sessions n freq Percentage
 <dbl> <int> <dbl> <chr>
 1 10 116 0.101 10.1%
 2 20 110 0.0953 9.5%
 3 30 96 0.0832 8.3%
 4 2 95 0.0823 8.2%
 5 4 95 0.0823 8.2%
 6 1 89 0.0771 7.7%
 7 15 81 0.0702 7%
 8 5 80 0.0693 6.9%
 9 3 76 0.0659 6.6%
10 8 61 0.0529 5.3%
# ℹ 28 more rows

- collapsing values over and including 61 (to make 61+ category)

#collapsing values over and including 61
over_60_range_01 <- fetch_combined %>%
 filter(fetch_combined$fetch_monthly_range == "71-80")
over_60_range_01 <- over_60_range_01 %>%
 mutate(fetch_monthly_range = "61+")

over_60_range_02 <- fetch_combined %>%
 filter(fetch_monthly_range == "81-90")
over_60_range_02 <- over_60_range_02 %>%
 mutate(fetch_monthly_range = "61+")

over_60_range_03 <- fetch_combined %>%
 filter(fetch_monthly_range == "91-100")
over_60_range_03 <- over_60_range_03 %>%
 mutate(fetch_monthly_range = "61+")

over_60_range_04 <- fetch_combined %>%
 filter(times_monthly_fetch_sessions == "120")
over_60_range_04 <- over_60_range_04 %>%
 mutate(fetch_monthly_range = "61+")

over_60_range_05 <- fetch_combined %>%
 filter(times_monthly_fetch_sessions == "180")
over_60_range_05 <- over_60_range_05 %>%
 mutate(fetch_monthly_range = "61+")

count_collapsed_fetching_sessions_monthly_range <- AppendMe(c("over_60_range_01","over_60_range_02","over_60_range_03","over_60_range_04","over_60_range_05"))

count_collapsed_fetching_sessions_monthly_range <- count_collapsed_fetching_sessions_monthly_range %>%
 select(-"source")

fetching_sessions_excl <- fetch_combined %>%
 filter(times_monthly_fetch_sessions != "75")
fetching_sessions_excl <- fetching_sessions_excl %>%
 filter(times_monthly_fetch_sessions != "80")
fetching_sessions_excl <- fetching_sessions_excl %>%
 filter(times_monthly_fetch_sessions != "90")
fetching_sessions_excl <- fetching_sessions_excl %>%
 filter(times_monthly_fetch_sessions != "100")
fetching_sessions_excl <- fetching_sessions_excl %>%
 filter(times_monthly_fetch_sessions != "120")
fetching_sessions_excl <- fetching_sessions_excl %>%
 filter(times_monthly_fetch_sessions != "180")

count_collapsed_fetching_sessions_monthly_range <- rbind(fetching_sessions_excl, count_collapsed_fetching_sessions_monthly_range)

### table of ranges

count_collapsed_fetching_sessions_monthly_range %>%
 group_by(fetch_monthly_range) %>%
 summarise(n = n()) %>%
 mutate(freq = n / sum(n)) %>%
 mutate(Percentage=paste0(round(freq/sum(freq)*100,1),"%")) %>%
 arrange(fetch_monthly_range)

# A tibble: 8 × 4
 fetch_monthly_range n freq Percentage
 <chr> <int> <dbl> <chr>
1 0-0.5 35 0.0303 3%
2 1-10 677 0.587 58.7%
3 11-20 237 0.205 20.5%
4 21-30 159 0.138 13.8%
5 31-40 14 0.0121 1.2%
6 41-50 7 0.00607 0.6%
7 51-60 13 0.0113 1.1%
8 61+ 12 0.0104 1%

### histogram

p <- ggplot(data=fetch_combined, aes(x = times_monthly_fetch_sessions)) +
 stat_bin(breaks = c(0, 10, 20, 30, 40, 50, 60, 70, 80, 90, 100, 110, 120, 130, 140, 150, 160, 170, 180), fill = "#ff9a8d", color = "black") +
 stat_bin(breaks = c(0, 10, 20, 30, 40, 50, 60, 70, 80, 90, 100, 110, 120, 130, 140, 150, 160, 170, 180), geom='text', color='black', aes(label=..count..), vjust = -1.1) +
 scale_x_continuous(breaks = seq(0,180,20)) +
 scale_y_continuous(breaks = seq(0, 800, 200)) +
 coord_cartesian(clip="off",ylim =c(0,800)) +
 xlab("\nNumber of \nmonthly fetching sessions") + ylab("\nFrequency")+
 theme_bw()+
 ggtitle("b") +
 theme(plot.title = element_text(hjust = -.22, size = 20, face = "bold")) +
 theme(legend.position = "None") +
 theme(axis.text.x = element_text(color = "black", size = 14)) +
 theme(axis.text.y = element_text(color = "black", size = 14)) +
 theme(axis.title.x = element_text(color= "black", face="bold", size = 14)) +
 theme(axis.title.y = element_text(color= "black", face="bold", size = 14)) +
 theme(axis.title.y = element_text(margin = margin(t = 0, r = 20, b = 0, l = 0)))
p


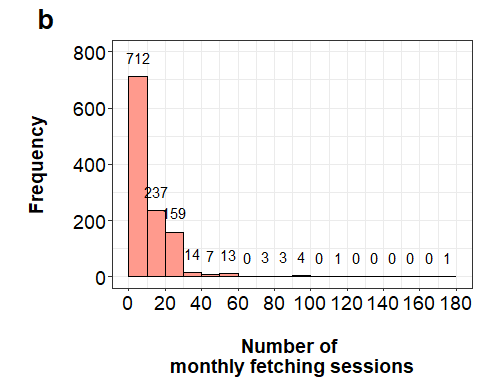


## multicat households

- excluding unclear responses:

** 51 responses that were contradictory (Yes to multicat household, but then answered that they only had one cat)

** 6 responses that answered “NA” to the numerical question

** 5 responses that just had “Yes” to both questions (no numerical response at all)

** 1 response that was contradictory “Yes” to multicat household but then answered with “No” when asked for the numerical response

** 1 response for kept isolated from other cat

** 1 response for being “2.5”

#51 responses excluded for "Yes" and "1". Cannot be "Yes" to a multicat household but only have 1 cat in total in the household
#the code below also excludes the 6 cats that have "Yes" to mutlticat household and then "NA" for total cats in household
#57 responses excluded here
total_cat_household_excl <- fetch_combined %>%
 filter(!(fetch_combined$multicat_household_time_fetch == "Yes" & fetch_combined$total_household_number_cats == "1"))

#51 cats that are contradictory
test_temp <- fetch_combined %>%
 filter(fetch_combined$multicat_household_time_fetch == "Yes" & fetch_combined$total_household_number_cats == "1")
#plus the 6 that are NA

#5 responses for both questions being answered as "Yes"
total_cat_household_excl <- total_cat_household_excl %>%
 filter(!(total_cat_household_excl$multicat_household_time_fetch == "Yes" & total_cat_household_excl$total_household_number_cats == "Yes"))

#1 response that was contradictory "Yes" then "No"
total_cat_household_excl <- total_cat_household_excl %>%
 filter(!(total_cat_household_excl$cat_name == "Odin" & total_cat_household_excl$total_household_number_cats == "No"))

#1 response for kept isolated from other cats
total_cat_household_excl <- total_cat_household_excl %>%
 filter(!(total_cat_household_excl$cat_name == "Pepper" & total_cat_household_excl$total_household_number_cats == "Yes but was kept isolated from them due to injury and had no interaction with them"))

#1 response for being "2.5"
total_cat_household_excl <- total_cat_household_excl %>%
 filter(!(total_cat_household_excl$cat_name == "Yoshimitsu"))

#### table of total cats in household

#total cats in household at time of fetching - grouped 7+
total_cat_household_excl %>%
 group_by(cats_household_condensed) %>%
 summarise(n = n()) %>%
 mutate(freq = n / sum(n)) %>%
 mutate(Percentage=paste0(round(freq/sum(freq)*100,1),"%")) %>%
 arrange(cats_household_condensed)

# A tibble: 7 × 4
 cats_household_condensed n freq Percentage
 <chr> <int> <dbl> <chr>
1 1 451 0.414 41.4%
2 2 383 0.352 35.2%
3 3 156 0.143 14.3%
4 4 46 0.0422 4.2%
5 5 26 0.0239 2.4%
6 6 12 0.0110 1.1%
7 7+ 15 0.0138 1.4%

#### table of other fetching cats in household

- still excluding the previous contradicting answers

#converting NA to "0s"
total_cat_household_excl$number_other_fetch_cats_household[is.na(total_cat_household_excl$number_other_fetch_cats_household)] <- 0

#single-cat households weren't shown this question in Qualtrics so is an automatic "NA" for them
total_cat_household_excl %>%
 group_by(number_other_fetch_cats_household) %>%
 summarise(n = n()) %>%
 mutate(freq = n / sum(n)) %>%
 mutate(Percentage=paste0(round(freq/sum(freq)*100,1),"%")) %>%
 arrange(number_other_fetch_cats_household)

# A tibble: 4 × 4
 number_other_fetch_cats_household n freq Percentage
 <dbl> <int> <dbl> <chr>
1 0 930 0.854 85.4%
2 1 134 0.123 12.3%
3 2 22 0.0202 2%
4 3 3 0.00275 0.3%

#excluding single-cat households
total_cat_household_excl %>%
 filter(total_household_number_cats != "1") %>%
 group_by(number_other_fetch_cats_household) %>%
 summarise(n = n()) %>%
 mutate(freq = n / sum(n)) %>%
 mutate(Percentage=paste0(round(freq/sum(freq)*100,1),"%")) %>%
 arrange(number_other_fetch_cats_household)

# A tibble: 4 × 4
 number_other_fetch_cats_household n freq Percentage
 <dbl> <int> <dbl> <chr>
1 0 479 0.751 75.1%
2 1 134 0.210 21%
3 2 22 0.0345 3.4%
4 3 3 0.00470 0.5%

# 2. PEOPLE/HOUSEHOLD DEMOGRAPHICS

## respondent gender

#count of person one gender of owner
fetch_combined %>%
 group_by(person_one_gender) %>%
 summarise(n = n()) %>%
 mutate(freq = n / sum(n)) %>%
 mutate(Percentage=paste0(round(freq/sum(freq)*100,1),"%")) %>%
 arrange(person_one_gender)

# A tibble: 4 × 4
 person_one_gender n freq Percentage
 <chr> <int> <dbl> <chr>
1 Female 748 0.648 64.8%
2 Male 266 0.231 23.1%
3 Non-binary 109 0.0945 9.4%
4 Prefer not to say 31 0.0269 2.7%

#excluding person one responses who did not disclose their gender
owner_gender_one_excl <- fetch_combined %>%
 dplyr::filter(person_one_gender != "Prefer not to say")

owner_gender_one_excl %>%
 group_by(person_one_gender) %>%
 summarise(n = n()) %>%
 mutate(freq = n / sum(n)) %>%
 mutate(Percentage=paste0(round(freq/sum(freq)*100,1),"%")) %>%
 arrange(person_one_gender)

# A tibble: 3 × 4
 person_one_gender n freq Percentage
 <chr> <int> <dbl> <chr>
1 Female 748 0.666 66.6%
2 Male 266 0.237 23.7%
3 Non-binary 109 0.0971 9.7%

## respondent age by gender

fetch_combined %>%
 filter(person_one_age != "NA") %>%
 group_by(person_one_gender) %>%
 summarise(mean = mean(person_one_age), sd = sd(person_one_age), min = min(person_one_age), max = max(person_one_age)) %>%
 arrange(person_one_gender)

# A tibble: 4 × 5
 person_one_gender mean sd min max
 <chr> <dbl> <dbl> <dbl> <dbl>
1 Female 34.7 11.4 6 73
2 Male 37.2 11.1 4 64
3 Non-binary 29.7 7.52 7 48
4 Prefer not to say 27.1 7.97 15 40

#respondent mean age and SD
mean(fetch_combined$person_one_age, na.rm = TRUE)

[1] 34.72884

sd(fetch_combined$person_one_age, na.rm = TRUE)

[1] 11.18438

range(fetch_combined$person_one_age, na.rm = TRUE)

[1] 4 73

#number of people who had a fetching cat when they were 'under' 18 years old
fetch_combined %>%
 filter(person_one_age < 18)

# A tibble: 40 × 75
 cat_name current_previous fetch_length_months_…¹ trained trained_description
 <chr> <chr> <dbl> <chr> <chr>
 1 "Kelso" Previous cat 3 Untrai… <NA>
 2 "Kougar" Previous cat 60 Trained Since he joined th…
 3 "Oliver" Previous cat 96 Untrai… <NA>
 4 "Ashes" Previous cat 24 Untrai… <NA>
 5 "Bailey" Previous cat 60 Trained We would toss an o…
 6 "Bob tai… Previous cat 84 Untrai… <NA>
 7 "Calixo … Previous cat 60 Untrai… <NA>
 8 "Casper" Previous cat 24 Untrai… <NA>
 9 "Echo" Previous cat 36 Untrai… <NA>
10 "Emily" Previous cat 60 Untrai… <NA>
# ℹ 30 more rows
# ℹ abbreviated name: ¹​fetch_length_months_total
# ℹ 70 more variables: untrained_description <chr>, object_one <chr>,
# object_two <chr>, object_three <chr>, object_four <chr>, object_five <chr>,
# object_six <chr>, object_seven <chr>, object_eight <chr>,
# object_nine <chr>, object_ten <chr>, object_extra <chr>,
# fave_object_group <chr>, object_preferred <chr>, …

#40 entries

## no. people in household at time of fetching behaviour

#number of people in the household at time of fetching behaviour
fetch_combined %>%
 group_by(ppl_count_household) %>%
 summarise(n = n()) %>%
 mutate(freq = n / sum(n)) %>%
 mutate(Percentage=paste0(round(freq/sum(freq)*100,1),"%")) %>%
 arrange(ppl_count_household)

# A tibble: 7 × 4
 ppl_count_household n freq Percentage
 <dbl> <int> <dbl> <chr>
1 1 220 0.191 19.1%
2 2 565 0.490 49%
3 3 212 0.184 18.4%
4 4 110 0.0953 9.5%
5 5 39 0.0338 3.4%
6 6 6 0.00520 0.5%
7 7 2 0.00173 0.2%

## average ages of households

- including “0” entries

#subsetting into households
one_person_household_mean <- fetch_combined %>%
 filter(ppl_count_household == "1")

two_person_household_mean <- fetch_combined %>%
 filter(ppl_count_household == "2")

three_person_household_mean <- fetch_combined %>%
 filter(ppl_count_household == "3")

four_person_household_mean <- fetch_combined %>%
 filter(ppl_count_household == "4")

five_person_household_mean <- fetch_combined %>%
 filter(ppl_count_household == "5")

six_person_household_mean <- fetch_combined %>%
 filter(ppl_count_household == "6")

seven_person_household_mean <- fetch_combined %>%
 filter(ppl_count_household == "7")

#generating one column of all of the raw ages per household
#two person households
two_person_household_mean <- two_person_household_mean %>%
 select(person_one_age, person_two_age)
two_person_household_mean <- two_person_household_mean %>%
 stack()

#three person households
three_person_household_mean <- three_person_household_mean %>%
 select(person_one_age, person_two_age, person_three_age)
three_person_household_mean <- three_person_household_mean %>%
 stack()

#four person households
four_person_household_mean <- four_person_household_mean %>%
 select(person_one_age, person_two_age, person_three_age, person_four_age)
four_person_household_mean <- four_person_household_mean %>%
 stack()

#five person households
five_person_household_mean <- five_person_household_mean %>%
 select(person_one_age, person_two_age, person_three_age, person_four_age, person_five_age)
five_person_household_mean <- five_person_household_mean %>%
 stack()

#six person households
six_person_household_mean <- six_person_household_mean %>%
 select(person_one_age, person_two_age, person_three_age, person_four_age, person_five_age, person_six_age)
six_person_household_mean <- six_person_household_mean %>%
 stack()

#seven person households
seven_person_household_mean <- seven_person_household_mean %>%
 select(person_one_age, person_two_age, person_three_age, person_four_age, person_five_age, person_six_age, person_seven_age)
seven_person_household_mean <- seven_person_household_mean %>%
 stack()

#one person household
one_person_household_mean <- one_person_household_mean %>%
 select(person_one_age) %>%
 filter(person_one_age != "NA") %>%
 summarise(mean = mean(person_one_age), sd = sd(person_one_age), min = min(person_one_age), max = max(person_one_age))

#two person household
two_person_household_mean <- two_person_household_mean %>%
 select(values) %>%
 filter(values != "NA") %>%
 summarise(mean = mean(values), sd = sd(values), min = min(values), max = max(values))

#three person household
three_person_household_mean <- three_person_household_mean %>%
 select(values) %>%
 filter(values != "NA") %>%
 summarise(mean = mean(values), sd = sd(values), min = min(values), max = max(values))

#four person household
four_person_household_mean <- four_person_household_mean %>%
 select(values) %>%
 filter(values != "NA") %>%
 summarise(mean = mean(values), sd = sd(values), min = min(values), max = max(values))

#five person household
five_person_household_mean <- five_person_household_mean %>%
 select(values) %>%
 filter(values != "NA") %>%
 summarise(mean = mean(values), sd = sd(values), min = min(values), max = max(values))

#six person household
six_person_household_mean <- six_person_household_mean %>%
 select(values) %>%
 filter(values != "NA") %>%
 summarise(mean = mean(values), sd = sd(values), min = min(values), max = max(values))

#seven person household
seven_person_household_mean <- seven_person_household_mean %>%
 select(values) %>%
 filter(values != "NA") %>%
 summarise(mean = mean(values), sd = sd(values), min = min(values), max = max(values))

#binding
household_means_sds <- AppendMe(c("one_person_household_mean","two_person_household_mean","three_person_household_mean","four_person_household_mean","five_person_household_mean","six_person_household_mean","seven_person_household_mean"))

household_means_sds <- household_means_sds %>%
 select(-source) %>%
 mutate(ppl_in_household = c(1,2,3,4,5,6,7)) %>%
 relocate(ppl_in_household, .before = mean)

### table of ages

#table
household_means_sds

ppl_in_household mean sd min max
1 1 34.80288 9.77795 20 73
2 2 35.58834 10.91388 0 72
3 3 36.25000 17.95132 0 88
4 4 31.62500 17.69177 0 86
5 5 33.54749 20.61476 1 87
6 6 24.77778 16.11585 2 64
7 7 27.00000 18.68360 10 73

## dogs present in household

** excluding 2 unclear responses for “Yes” and then “N/A” for the specific number of dogs in the household

#excluding dogs based on the above criteria
total_dogs_household_excl <- fetch_combined %>%
 filter(cat_name != "Wolfgang")
total_dogs_household_excl <- total_dogs_household_excl %>%
 filter(cat_name != "Cleo")
total_dogs_household_excl <- total_dogs_household_excl %>%
 filter(cat_name != "Muddlety")

### table

#dogs present in household
total_dogs_household_excl %>%
 group_by(dogs_household) %>%
 summarise(n = n()) %>%
 mutate(freq = n / sum(n)) %>%
 mutate(Percentage=paste0(round(freq/sum(freq)*100,1),"%"))

# A tibble: 2 × 4
 dogs_household n freq Percentage
 <chr> <int> <dbl> <chr>
1 No 989 0.859 85.9%
2 Yes 162 0.141 14.1%

## number of dogs in household

#number of dogs present in household
total_dogs_household_excl %>%
 group_by(dogs_household_condensed) %>%
 summarise(n = n()) %>%
 mutate(freq = n / sum(n)) %>%
 mutate(Percentage=paste0(round(freq/sum(freq)*100,1),"%"))

# A tibble: 4 × 4
 dogs_household_condensed n freq Percentage
 <chr> <int> <dbl> <chr>
1 1 103 0.0895 8.9%
2 2 47 0.0408 4.1%
3 3+ 12 0.0104 1%
4 <NA> 989 0.859 85.9%

## number of fetching dogs in household

#out of the dogs that were recognised to be fetching dogs
total_dogs_household_excl %>%
 filter(number_dogs_household_fetch != "NA") %>%
 group_by(number_dogs_household_fetch) %>%
 summarise(n = n()) %>%
 mutate(freq = n / sum(n)) %>%
 mutate(Percentage=paste0(round(freq/sum(freq)*100,1),"%"))

# A tibble: 4 × 4
 number_dogs_household_fetch n freq Percentage
 <dbl> <int> <dbl> <chr>
1 0 56 0.346 34.6%
2 1 81 0.5 50%
3 2 23 0.142 14.2%
4 3 2 0.0123 1.2%

## would cat have seen dog fetch prior to fetching themselves

#NOTE: this only includes dogs that were already known to fetch and excludes dogs that did not fetch
total_dogs_household_excl %>%
 filter(number_dogs_household_fetch != "0") %>%
 filter(cat_see_dog_fetch != "NA") %>%
 group_by(cat_see_dog_fetch) %>%
 summarise(n = n()) %>%
 mutate(freq = n / sum(n)) %>%
 mutate(Percentage=paste0(round(freq/sum(freq)*100,1),"%"))

# A tibble: 2 × 4
 cat_see_dog_fetch n freq Percentage
 <chr> <int> <dbl> <chr>
1 No 72 0.679 67.9%
2 Yes 34 0.321 32.1%

## 3. INFERENTIAL STATISTICS

### A. Frequency counts

#### Count of female to male cats

#has to be table format (not data.frame)
contingency_table_sex <- table(fetch_combined$cat_sex)

#make sure smallest expected frequency is higher than five
(Xsq <- chisq.test(contingency_table_sex))

Chi-squared test for given probabilities

data: contingency_table_sex
X-squared = 5.5459, df = 1, p-value = 0.01852

Xsq$observed # observed counts

Female Male
 537 617

Xsq$expected # expected counts under the null

Female Male
 577 577

Xsq$residuals # Pearson residuals

Female Male
-1.665222 1.665222

Xsq$stdres # standardized residuals

Female Male
-2.354979 2.354979

#### Count of initatiors

#initiators of fetch
#has to be table format (not data.frame)
contingency_table_initiators <- table(fetch_combined$initiate_fetch)

#make sure smallest expected frequency is higher than five
(Xsq <- chisq.test(contingency_table_initiators))

Chi-squared test for given probabilities

data: contingency_table_initiators
X-squared = 124.29, df = 2, p-value < 2.2e-16

Xsq$observed # observed counts

About equal Cat Myself
 352 553 249

Xsq$expected # expected counts under the null

About equal Cat Myself
 384.6667 384.6667 384.6667

Xsq$residuals # Pearson residuals

About equal Cat Myself
 -1.665569 8.582777 -6.917209

Xsq$stdres # standardized residuals

About equal Cat Myself
 -2.039897 10.511713 -8.471816

##### posthoc

#Chi-square subtables for posthoc
cat_owner_initiators <- fetch_combined %>%
 filter(initiate_fetch != "About equal")

cat_equal_initiators <- fetch_combined %>%
 filter(initiate_fetch != "Myself")

owner_equal_initiators <- fetch_combined %>%
 filter(initiate_fetch != "Cat")

#table format for chi-square
contingency_table_cat_owner_initiators <- table(cat_owner_initiators$initiate_fetch)

contingency_table_cat_equal_initiators <- table(cat_equal_initiators$initiate_fetch)

contingency_table_owner_equal_initiators <- table(owner_equal_initiators$initiate_fetch)

#Alpha needs to be interpreted as 0.05/3 = .0166
#Cat vs. Owner
(Xsq <- chisq.test(contingency_table_cat_owner_initiators))

Chi-squared test for given probabilities

data: contingency_table_cat_owner_initiators
X-squared = 115.23, df = 1, p-value < 2.2e-16

Xsq$observed # observed counts

Cat Myself
 553 249

Xsq$expected # expected counts under the null

Cat Myself
 401 401

Xsq$residuals # Pearson residuals

Cat Myself
 7.590518 -7.590518

Xsq$stdres # standardized residuals

Cat Myself
 10.73461 -10.73461

#Alpha needs to be interpreted as 0.05/3 = .0166
#Cat vs. About equal
(Xsq <- chisq.test(contingency_table_cat_equal_initiators))

Chi-squared test for given probabilities

data: contingency_table_cat_equal_initiators
X-squared = 44.642, df = 1, p-value = 2.366e-11

Xsq$observed # observed counts

About equal Cat
 352 553

Xsq$expected # expected counts under the null

About equal Cat
 452.5 452.5

Xsq$residuals # Pearson residuals

About equal Cat
 -4.72451 4.72451

Xsq$stdres # standardized residuals

About equal Cat
 -6.681466 6.681466

#Alpha needs to be interpreted as 0.05/3 = .0166
#Owner vs. About equal
(Xsq <- chisq.test(contingency_table_owner_equal_initiators))

Chi-squared test for given probabilities

data: contingency_table_owner_equal_initiators
X-squared = 17.652, df = 1, p-value = 2.652e-05

Xsq$observed # observed counts

About equal Myself
 352 249

Xsq$expected # expected counts under the null

About equal Myself
 300.5 300.5

Xsq$residuals # Pearson residuals

About equal Myself
 2.970879 -2.970879

Xsq$stdres # standardized residuals

About equal Myself
 4.201458 -4.201458

#### Count of enders

#ending fetch
#has to be table format (not data.frame)
contingency_table_enders <- table(fetch_combined$end_fetch)

#make sure smallest expected frequency is higher than five
(Xsq <- chisq.test(contingency_table_enders))

Chi-squared test for given probabilities

data: contingency_table_enders
X-squared = 329.08, df = 2, p-value < 2.2e-16

Xsq$observed # observed counts

About equal Cat Myself
 231 675 248

Xsq$expected # expected counts under the null

About equal Cat Myself
 384.6667 384.6667 384.6667

Xsq$residuals # Pearson residuals

About equal Cat Myself
 -7.834971 14.803166 -6.968195

Xsq$stdres # standardized residuals

About equal Cat Myself
 -9.595841 18.130102 -8.534262

##### posthoc

#Chi-square subtables for posthoc
cat_owner_enders <- fetch_combined %>%
 filter(end_fetch != "About equal")

cat_equal_enders <- fetch_combined %>%
 filter(end_fetch != "Myself")

owner_equal_enders <- fetch_combined %>%
 filter(end_fetch != "Cat")

#table format for chi-square
contingency_table_cat_owner_enders <- table(cat_owner_enders$end_fetch)

contingency_table_cat_equal_enders <- table(cat_equal_enders$end_fetch)

contingency_table_owner_equal_enders <- table(owner_equal_enders$end_fetch)

#Alpha needs to be interpreted as 0.05/3 = .0166
#Cat vs. Owner
(Xsq <- chisq.test(contingency_table_cat_owner_enders))

Chi-squared test for given probabilities

data: contingency_table_cat_owner_enders
X-squared = 197.54, df = 1, p-value < 2.2e-16

Xsq$observed # observed counts

Cat Myself
 675 248

Xsq$expected # expected counts under the null

Cat Myself
 461.5 461.5

Xsq$residuals # Pearson residuals

Cat Myself
 9.938298 -9.938298

Xsq$stdres # standardized residuals

Cat Myself
 14.05488 -14.05488

#Alpha needs to be interpreted as 0.05/3 = .0166
#Cat vs. About equal
(Xsq <- chisq.test(contingency_table_cat_equal_enders))

Chi-squared test for given probabilities

data: contingency_table_cat_equal_enders
X-squared = 217.59, df = 1, p-value < 2.2e-16

Xsq$observed # observed counts

About equal Cat
 231 675

Xsq$expected # expected counts under the null

About equal Cat
 453 453

Xsq$residuals # Pearson residuals

About equal Cat
 -10.43047 10.43047

Xsq$stdres # standardized residuals

About equal Cat
 -14.75091 14.75091

#Alpha needs to be interpreted as 0.05/3 = .0166
#Owner vs. About equal
(Xsq <- chisq.test(contingency_table_owner_equal_enders))

Chi-squared test for given probabilities

data: contingency_table_owner_equal_enders
X-squared = 0.60334, df = 1, p-value = 0.4373

Xsq$observed # observed counts

About equal Myself
 231 248

Xsq$expected # expected counts under the null

About equal Myself
 239.5 239.5

Xsq$residuals # Pearson residuals

About equal Myself
 -0.5492451 0.5492451

Xsq$stdres # standardized residuals

About equal Myself
 -0.7767498 0.7767498

### B. Cat sex x initiation/end

#### Cat sex x initiation of fetch

#has to be table format (not data.frame)
contingency_sex_initiation <- table(fetch_combined$cat_sex, fetch_combined$initiate_fetch)

#make sure smallest expected frequency is higher than five
(Xsq <- chisq.test(contingency_sex_initiation))

Pearson's Chi-squared test

data: contingency_sex_initiation
X-squared = 1.8163, df = 2, p-value = 0.4033

Xsq$observed # observed counts

About equal Cat Myself
 Female 164 266 107
 Male 188 287 142

Xsq$expected # expected counts under the null

About equal Cat Myself
 Female 163.799 257.3319 115.8692
 Male 188.201 295.6681 133.1308

Xsq$residuals # Pearson residuals

About equal Cat Myself
 Female 0.01570821 0.54035311 -0.82394482
 Male -0.01465451 -0.50410641 0.76867488

Xsq$stdres # standardized residuals

About equal Cat Myself
 Female 0.0257693 1.0240087 -1.2724393
 Male -0.0257693 -1.0240087 1.2724393

#### Cat sex x ending of fetch

#has to be table format (not data.frame)
contingency_sex_end <- table(fetch_combined$cat_sex, fetch_combined$end_fetch)

#make sure smallest expected frequency is higher than five
(Xsq <- chisq.test(contingency_sex_end))

Pearson's Chi-squared test

data: contingency_sex_end
X-squared = 1.9667, df = 2, p-value = 0.3741

Xsq$observed # observed counts

About equal Cat Myself
 Female 117 307 113
 Male 114 368 135

Xsq$expected # expected counts under the null

About equal Cat Myself
 Female 107.4931 314.1031 115.4038
 Male 123.5069 360.8969 132.5962

Xsq$residuals # Pearson residuals

About equal Cat Myself
 Female 0.9169595 -0.4007864 -0.2237642
 Male -0.8554502 0.3739018 0.2087542

Xsq$stdres # standardized residuals

About equal Cat Myself
 Female 1.4022084 -0.8507628 -0.3453740
 Male -1.4022084 0.8507628 0.3453740

### C. Recent object retrievals

#### No. retrievals recent session x initiators

- Following these websites: <https://www.scribbr.co.uk/stats/ordinal-data-meaning/> <http://www.sthda.com/english/wiki/kruskal-wallis-test-in-r> <https://www.datanovia.com/en/lessons/wilcoxon-test-in-r/>

#making appropriate dataset
fetch_sessions_recent <- fetch_combined %>%
 select(initiate_fetch, times_object_retrieved_recent)

#ensuring as a factor
fetch_sessions_recent$initiate_fetch<-as.factor(fetch_sessions_recent$initiate_fetch)

#descriptive stats
fetch_sessions_recent %>% select(initiate_fetch, times_object_retrieved_recent) %>% group_by(initiate_fetch) %>%
 summarise(n = n(),
 mean = mean(times_object_retrieved_recent, na.rm = TRUE),
 sd = sd(times_object_retrieved_recent, na.rm = TRUE),
 stderr = sd/sqrt(n),
 LCI = mean - qt(1 - (0.05 / 2), n - 1) * stderr,
 UCI = mean + qt(1 - (0.05 / 2), n - 1) * stderr,
 median = median(times_object_retrieved_recent, na.rm = TRUE),
 min = min(times_object_retrieved_recent, na.rm = TRUE),
 max = max(times_object_retrieved_recent, na.rm = TRUE),
 IQR = IQR(times_object_retrieved_recent, na.rm = TRUE),
 LCImed = MedianCI(times_object_retrieved_recent, na.rm=TRUE)[2],
 UCImed = MedianCI(times_object_retrieved_recent, na.rm=TRUE)[3])

# A tibble: 3 × 13
 initiate_fetch n mean sd stderr LCI UCI median min max IQR
 <fct> <int> <dbl> <dbl> <dbl> <dbl> <dbl> <dbl> <dbl> <dbl> <dbl>
1 About equal 352 7.17 5.79 0.308 6.57 7.78 6 1 68 6
2 Cat 553 7.17 5.94 0.253 6.67 7.67 5 0 50 6
3 Myself 249 5.86 4.31 0.273 5.33 6.40 5 0 30 4
# ℹ 2 more variables: LCImed <dbl>, UCImed <dbl>

#count of each range in a bar graph
g <-ggplot(data=fetch_sessions_recent, aes(initiate_fetch))
g + geom_bar()


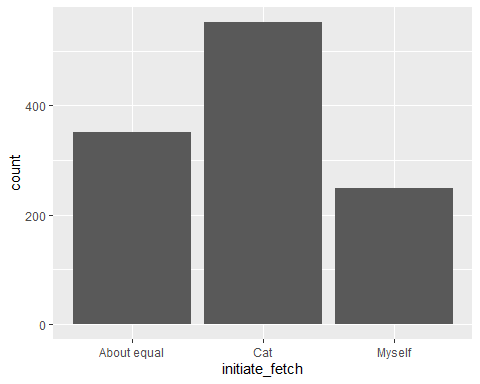


#outliers
#some outliers
ggplot(fetch_sessions_recent, aes(x = initiate_fetch, y = times_object_retrieved_recent, fill = initiate_fetch)) +
 stat_boxplot(geom ="errorbar", width = 0.5) +
 geom_boxplot(fill = "light blue") +
 stat_summary(fun.y=mean, geom="point", shape=10, size=3.5, color="black") +
 ggtitle("Boxplots") +
 theme_bw() + theme(legend.position="none")


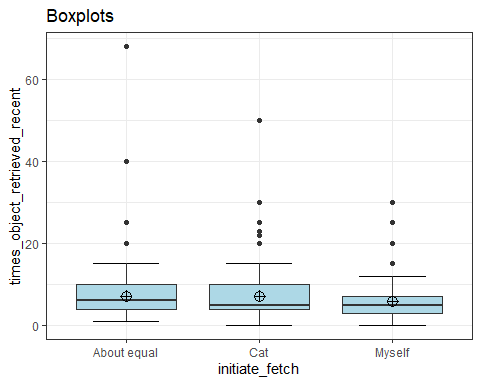


#identifies extreme outliers
#Values above Q3 + 3xIQR or below Q1 - 3xIQR are considered as extreme points (or extreme outliers)
fetch_sessions_recent %>%
 group_by(initiate_fetch) %>%
 identify_outliers(times_object_retrieved_recent) %>%
 filter(is.extreme == "TRUE")

# A tibble: 22 × 4
 initiate_fetch times_object_retrieved_recent is.outlier is.extreme
 <fct> <dbl> <lgl> <lgl>
 1 About equal 40 TRUE TRUE
 2 About equal 68 TRUE TRUE
 3 Cat 30 TRUE TRUE
 4 Cat 30 TRUE TRUE
 5 Cat 50 TRUE TRUE
 6 Cat 30 TRUE TRUE
 7 Cat 30 TRUE TRUE
 8 Cat 30 TRUE TRUE
 9 Cat 30 TRUE TRUE
10 Cat 50 TRUE TRUE
# ℹ 12 more rows

#normality checks
#all significant results so data is not normally distributed
fetch_sessions_recent %>%
 group_by(initiate_fetch) %>%
 summarise(`W Stat` = shapiro.test(times_object_retrieved_recent)$statistic,
 p.value = shapiro.test(times_object_retrieved_recent)$p.value)

# A tibble: 3 × 3
 initiate_fetch `W Stat` p.value
 <fct> <dbl> <dbl>
1 About equal 0.671 1.41e-25
2 Cat 0.742 1.99e-28
3 Myself 0.768 1.89e-18

#QQ plots
#non-normal distributions
ggplot(data = fetch_sessions_recent, mapping = aes(sample = times_object_retrieved_recent, color = initiate_fetch, fill = initiate_fetch)) +
 stat_qq_band(alpha=0.5, conf=0.95, qtype=1, bandType = "boot") +
 stat_qq_line(identity=TRUE) +
 stat_qq_point(col="black") +
 facet_wrap(~ initiate_fetch, scales = "free") +
 labs(x = "Theoretical Quantiles", y = "Sample Quantiles") + theme_bw()


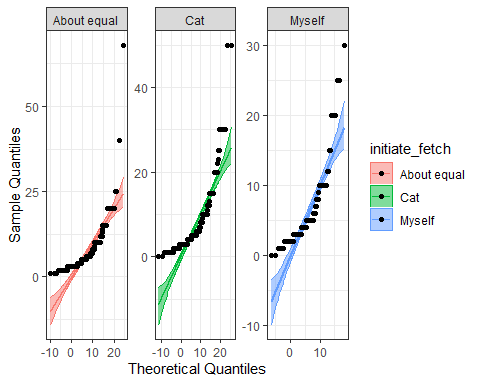


#so non-parametric tests are suitable

#Kruskal-Wallis test
#significant differences between groups
kruskal.test(times_object_retrieved_recent ~ initiate_fetch, data = fetch_sessions_recent)

Kruskal-Wallis rank sum test

data: times_object_retrieved_recent by initiate_fetch
Kruskal-Wallis chi-squared = 13.976, df = 2, p-value = 0.0009229

#Levene's test to test for equal variances
leveneTest(times_object_retrieved_recent ~ initiate_fetch, fetch_sessions_recent)

Levene's Test for Homogeneity of Variance (center = median)
 Df F value Pr(>F)
group 2 3.6907 0.02525 *
 1151
---
Signif. codes: 0 '***' 0.001 '**' 0.01 '*' 0.05 '.' 0.1 ' ' 1

#significant so there are not equal variances = bonferroni is good alternative to Tukey

#pairwise comparisons using Bonferroni correction
pairwise.wilcox.test(fetch_sessions_recent$times_object_retrieved_recent, fetch_sessions_recent$initiate_fetch,
 p.adjust.method = "bonferroni")

Pairwise comparisons using Wilcoxon rank sum test with continuity correction

data: fetch_sessions_recent$times_object_retrieved_recent and fetch_sessions_recent$initiate_fetch

 About equal Cat
Cat 0.99585 -
Myself 0.00062 0.00934

P value adjustment method: bonferroni

##### excluding extreme outliers

#data of only the extreme outliers
fetch_outliers_recent <- fetch_sessions_recent %>%
 group_by(initiate_fetch) %>%
 identify_outliers(times_object_retrieved_recent) %>%
 filter(is.extreme == "TRUE")
fetch_outliers_recent

# A tibble: 22 × 4
 initiate_fetch times_object_retrieved_recent is.outlier is.extreme
 <fct> <dbl> <lgl> <lgl>
 1 About equal 40 TRUE TRUE
 2 About equal 68 TRUE TRUE
 3 Cat 30 TRUE TRUE
 4 Cat 30 TRUE TRUE
 5 Cat 50 TRUE TRUE
 6 Cat 30 TRUE TRUE
 7 Cat 30 TRUE TRUE
 8 Cat 30 TRUE TRUE
 9 Cat 30 TRUE TRUE
10 Cat 50 TRUE TRUE
# ℹ 12 more rows

#selecting columns
fetch_outliers_recent <- fetch_outliers_recent %>%
 select(initiate_fetch, times_object_retrieved_recent)

#then removing duplicates to exclude the extreme outliers from the dataset
fetch_recent_outliers_excl <- anti_join(fetch_sessions_recent, fetch_outliers_recent)

#re-running same analysis to see if outliers affect significance
#ensuring as a factor
fetch_recent_outliers_excl$initiate_fetch<-as.factor(fetch_recent_outliers_excl$initiate_fetch)

#descriptive stats
fetch_recent_outliers_excl %>% select(initiate_fetch, times_object_retrieved_recent) %>% group_by(initiate_fetch) %>%
 summarise(n = n(),
 mean = mean(times_object_retrieved_recent, na.rm = TRUE),
 sd = sd(times_object_retrieved_recent, na.rm = TRUE),
 stderr = sd/sqrt(n),
 LCI = mean - qt(1 - (0.05 / 2), n - 1) * stderr,
 UCI = mean + qt(1 - (0.05 / 2), n - 1) * stderr,
 median = median(times_object_retrieved_recent, na.rm = TRUE),
 min = min(times_object_retrieved_recent, na.rm = TRUE),
 max = max(times_object_retrieved_recent, na.rm = TRUE),
 IQR = IQR(times_object_retrieved_recent, na.rm = TRUE),
 LCImed = MedianCI(times_object_retrieved_recent, na.rm=TRUE)[2],
 UCImed = MedianCI(times_object_retrieved_recent, na.rm=TRUE)[3])

# A tibble: 3 × 13
 initiate_fetch n mean sd stderr LCI UCI median min max IQR
 <fct> <int> <dbl> <dbl> <dbl> <dbl> <dbl> <dbl> <dbl> <dbl> <dbl>
1 About equal 350 6.91 4.46 0.239 6.44 7.37 6 1 25 5.75
2 Cat 542 6.63 4.50 0.193 6.25 7.01 5 0 25 6
3 Myself 240 5.25 2.90 0.187 4.88 5.62 5 0 15 4
# ℹ 2 more variables: LCImed <dbl>, UCImed <dbl>

#count of each range in a bar graph
g <-ggplot(data=fetch_recent_outliers_excl, aes(initiate_fetch))
g + geom_bar()


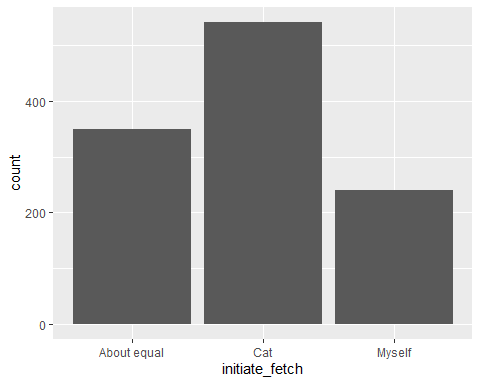


#outliers
#some outliers
ggplot(fetch_recent_outliers_excl, aes(x = initiate_fetch, y = times_object_retrieved_recent, fill = initiate_fetch)) +
 stat_boxplot(geom ="errorbar", width = 0.5) +
 geom_boxplot(fill = "light blue") +
 stat_summary(fun.y=mean, geom="point", shape=10, size=3.5, color="black") +
 ggtitle("Boxplots") +
 theme_bw() + theme(legend.position="none")


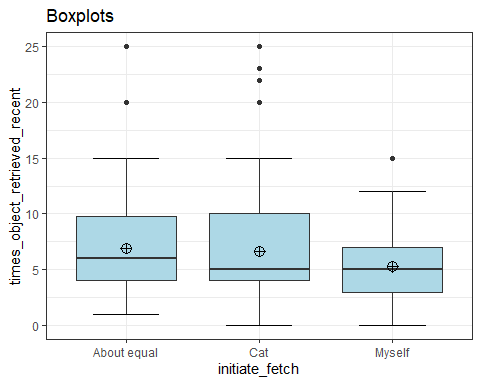


#identifies extreme outliers
#Values above Q3 + 3xIQR or below Q1 - 3xIQR are considered as extreme points (or extreme outliers)
#no extreme outliers anymore
fetch_recent_outliers_excl %>%
 group_by(initiate_fetch) %>%
 identify_outliers(times_object_retrieved_recent) %>%
 filter(is.extreme == "TRUE")

# A tibble: 0 × 4
# ℹ 4 variables: initiate_fetch <fct>, times_object_retrieved_recent <dbl>,
# is.outlier <lgl>, is.extreme <lgl>

#normality checks
#all significant results so data is not normally distributed
fetch_recent_outliers_excl %>%
 group_by(initiate_fetch) %>%
 summarise(`W Stat` = shapiro.test(times_object_retrieved_recent)$statistic,
 p.value = shapiro.test(times_object_retrieved_recent)$p.value)

# A tibble: 3 × 3
 initiate_fetch `W Stat` p.value
 <fct> <dbl> <dbl>
1 About equal 0.842 2.73e-18
2 Cat 0.853 4.25e-22
3 Myself 0.902 2.15e-11

#QQ plots
ggplot(data = fetch_recent_outliers_excl, mapping = aes(sample = times_object_retrieved_recent, color = initiate_fetch, fill = initiate_fetch)) +
 stat_qq_band(alpha=0.5, conf=0.95, qtype=1, bandType = "boot") +
 stat_qq_line(identity=TRUE) +
 stat_qq_point(col="black") +
 facet_wrap(~ initiate_fetch, scales = "free") +
 labs(x = "Theoretical Quantiles", y = "Sample Quantiles") + theme_bw()


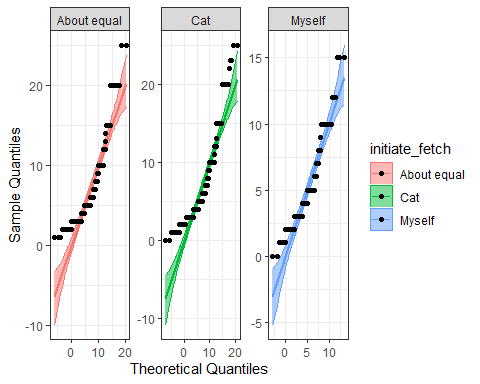


#so non-parametric tests are suitable

#Kruskal-Wallis test
#significant differences between groups
kruskal.test(times_object_retrieved_recent ~ initiate_fetch, data = fetch_recent_outliers_excl)

Kruskal-Wallis rank sum test

data: times_object_retrieved_recent by initiate_fetch
Kruskal-Wallis chi-squared = 19.914, df = 2, p-value = 4.739e-05

#Levene's test to test for equal variances
leveneTest(times_object_retrieved_recent ~ initiate_fetch, fetch_recent_outliers_excl)

Levene's Test for Homogeneity of Variance (center = median)
 Df F value Pr(>F)
group 2 9.3923 9.006e-05 ***
 1129
---
Signif. codes: 0 '***' 0.001 '**' 0.01 '*' 0.05 '.' 0.1 ' ' 1

#significant so there are not equal variances = bonferroni is good alternative to Tukey

#pairwise comparisons using Bonferroni correction
pairwise.wilcox.test(fetch_recent_outliers_excl$times_object_retrieved_recent, fetch_recent_outliers_excl$initiate_fetch,
 p.adjust.method = "bonferroni")

Pairwise comparisons using Wilcoxon rank sum test with continuity correction

data: fetch_recent_outliers_excl$times_object_retrieved_recent and fetch_recent_outliers_excl$initiate_fetch

 About equal Cat
Cat 0.5229 -
Myself 2.5e-05 0.0018

P value adjustment method: bonferroni

#### No. retrievals recent session x enders

#making appropriate dataset
fetch_sessions_recent_end <- fetch_combined %>%
 select(end_fetch, times_object_retrieved_recent)

#ensuring as a factor
fetch_sessions_recent_end$end_fetch<-as.factor(fetch_sessions_recent_end$end_fetch)

#descriptive stats
fetch_sessions_recent_end %>%
 group_by(end_fetch) %>%
 summarise(n = n(),
 mean = mean(times_object_retrieved_recent, na.rm = TRUE),
 sd = sd(times_object_retrieved_recent, na.rm = TRUE),
 stderr = sd/sqrt(n),
 LCI = mean - qt(1 - (0.05 / 2), n - 1) * stderr,
 UCI = mean + qt(1 - (0.05 / 2), n - 1) * stderr,
 median = median(times_object_retrieved_recent, na.rm = TRUE),
 min = min(times_object_retrieved_recent, na.rm = TRUE),
 max = max(times_object_retrieved_recent, na.rm = TRUE),
 IQR = IQR(times_object_retrieved_recent, na.rm = TRUE),
 LCImed = MedianCI(times_object_retrieved_recent, na.rm=TRUE)[2],
 UCImed = MedianCI(times_object_retrieved_recent, na.rm=TRUE)[3])

# A tibble: 3 × 13
 end_fetch n mean sd stderr LCI UCI median min max IQR LCImed
 <fct> <int> <dbl> <dbl> <dbl> <dbl> <dbl> <dbl> <dbl> <dbl> <dbl> <dbl>
1 About eq… 231 7.68 6.63 0.436 6.82 8.53 6 1 68 6 5
2 Cat 675 5.75 4.44 0.171 5.41 6.08 5 0 50 4 4
3 Myself 248 9.27 6.47 0.411 8.46 10.1 8 0 50 5 7
# ℹ 1 more variable: UCImed <dbl>

#count of each range in a bar graph
g <-ggplot(data=fetch_sessions_recent_end, aes(end_fetch))
g + geom_bar()


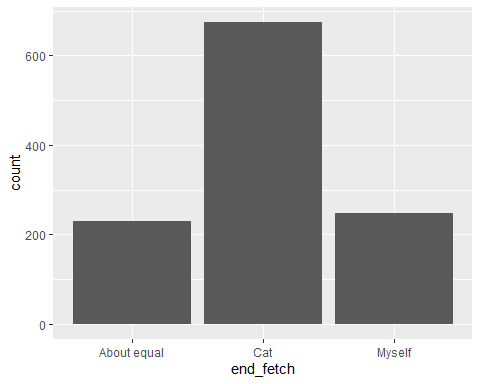


#outliers
#some outliers
ggplot(fetch_sessions_recent_end, aes(x = end_fetch, y = times_object_retrieved_recent, fill = end_fetch)) +
 stat_boxplot(geom ="errorbar", width = 0.5) +
 geom_boxplot(fill = "light blue") +
 stat_summary(fun.y=mean, geom="point", shape=10, size=3.5, color="black") +
 ggtitle("Boxplots") +
 theme_bw() + theme(legend.position="none")


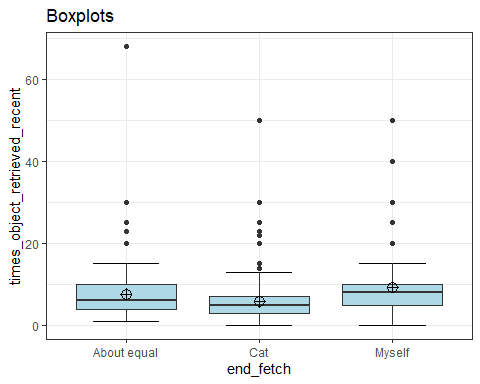


#identifies extreme outliers
#Values above Q3 + 3xIQR or below Q1 - 3xIQR are considered as extreme points (or extreme outliers)
fetch_sessions_recent_end %>%
 group_by(end_fetch) %>%
 identify_outliers(times_object_retrieved_recent) %>%
 filter(is.extreme == "TRUE")

# A tibble: 29 × 4
 end_fetch times_object_retrieved_recent is.outlier is.extreme
 <fct> <dbl> <lgl> <lgl>
 1 About equal 68 TRUE TRUE
 2 About equal 30 TRUE TRUE
 3 About equal 30 TRUE TRUE
 4 About equal 30 TRUE TRUE
 5 Cat 20 TRUE TRUE
 6 Cat 20 TRUE TRUE
 7 Cat 30 TRUE TRUE
 8 Cat 20 TRUE TRUE
 9 Cat 25 TRUE TRUE
10 Cat 30 TRUE TRUE
# ℹ 19 more rows

#normality checks
#all significant results so data is not normally distributed
fetch_sessions_recent_end %>%
 group_by(end_fetch) %>%
 summarise(`W Stat` = shapiro.test(times_object_retrieved_recent)$statistic,
 p.value = shapiro.test(times_object_retrieved_recent)$p.value)

# A tibble: 3 × 3
 end_fetch `W Stat` p.value
 <fct> <dbl> <dbl>
1 About equal 0.668 4.86e-21
2 Cat 0.718 3.58e-32
3 Myself 0.803 5.16e-17

#QQ plots
#non-normal distributions
ggplot(data = fetch_sessions_recent_end, mapping = aes(sample = times_object_retrieved_recent, color = end_fetch, fill = end_fetch)) +
 stat_qq_band(alpha=0.5, conf=0.95, qtype=1, bandType = "boot") +
 stat_qq_line(identity=TRUE) +
 stat_qq_point(col="black") +
 facet_wrap(~ end_fetch, scales = "free") +
 labs(x = "Theoretical Quantiles", y = "Sample Quantiles") + theme_bw()


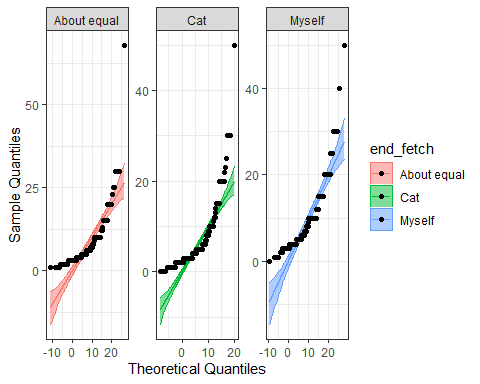


#so non-parametric tests are suitable

#Kruskal-Wallis test
#significant differences between groups
kruskal.test(times_object_retrieved_recent ~ end_fetch, data = fetch_sessions_recent_end)

Kruskal-Wallis rank sum test

data: times_object_retrieved_recent by end_fetch
Kruskal-Wallis chi-squared = 110.55, df = 2, p-value < 2.2e-16

#Levene's test to test for equal variances
leveneTest(times_object_retrieved_recent ~ end_fetch, fetch_sessions_recent_end)

Levene's Test for Homogeneity of Variance (center = median)
 Df F value Pr(>F)
group 2 16.576 7.999e-08 ***
 1151
---
Signif. codes: 0 '***' 0.001 '**' 0.01 '*' 0.05 '.' 0.1 ' ' 1

#significant so there are not equal variances = bonferroni is good alternative to Tukey

#pairwise comparisons using Bonferroni correction
pairwise.wilcox.test(fetch_sessions_recent_end$times_object_retrieved_recent, fetch_sessions_recent_end$end_fetch,
 p.adjust.method = "bonferroni")

Pairwise comparisons using Wilcoxon rank sum test with continuity correction

data: fetch_sessions_recent_end$times_object_retrieved_recent and fetch_sessions_recent_end$end_fetch

 About equal Cat
Cat 4e-07 -
Myself 0.00023 < 2e-16

P value adjustment method: bonferroni

##### excluding extreme outliers

#data of only the extreme outliers
fetch_outliers_recent_end <- fetch_sessions_recent_end %>%
 group_by(end_fetch) %>%
 identify_outliers(times_object_retrieved_recent) %>%
 filter(is.extreme == "TRUE")
fetch_outliers_recent_end

# A tibble: 29 × 4
 end_fetch times_object_retrieved_recent is.outlier is.extreme
 <fct> <dbl> <lgl> <lgl>
 1 About equal 68 TRUE TRUE
 2 About equal 30 TRUE TRUE
 3 About equal 30 TRUE TRUE
 4 About equal 30 TRUE TRUE
 5 Cat 20 TRUE TRUE
 6 Cat 20 TRUE TRUE
 7 Cat 30 TRUE TRUE
 8 Cat 20 TRUE TRUE
 9 Cat 25 TRUE TRUE
10 Cat 30 TRUE TRUE
# ℹ 19 more rows

#selecting columns
fetch_outliers_recent_end <- fetch_outliers_recent_end %>%
 select(end_fetch, times_object_retrieved_recent)

#then removing duplicates to exclude the extreme outliers from the dataset
fetch_recent_outliers_excl_end <- anti_join(fetch_sessions_recent_end, fetch_outliers_recent_end)

#re-running same analysis to see if outliers affect significance
#ensuring as a factor
fetch_recent_outliers_excl_end$end_fetch<-as.factor(fetch_recent_outliers_excl_end$end_fetch)

#descriptive stats
fetch_recent_outliers_excl_end %>% select(end_fetch, times_object_retrieved_recent) %>% group_by(end_fetch) %>%
 summarise(n = n(),
 mean = mean(times_object_retrieved_recent, na.rm = TRUE),
 sd = sd(times_object_retrieved_recent, na.rm = TRUE),
 stderr = sd/sqrt(n),
 LCI = mean - qt(1 - (0.05 / 2), n - 1) * stderr,
 UCI = mean + qt(1 - (0.05 / 2), n - 1) * stderr,
 median = median(times_object_retrieved_recent, na.rm = TRUE),
 min = min(times_object_retrieved_recent, na.rm = TRUE),
 max = max(times_object_retrieved_recent, na.rm = TRUE),
 IQR = IQR(times_object_retrieved_recent, na.rm = TRUE),
 LCImed = MedianCI(times_object_retrieved_recent, na.rm=TRUE)[2],
 UCImed = MedianCI(times_object_retrieved_recent, na.rm=TRUE)[3])

# A tibble: 3 × 13
 end_fetch n mean sd stderr LCI UCI median min max IQR LCImed
 <fct> <int> <dbl> <dbl> <dbl> <dbl> <dbl> <dbl> <dbl> <dbl> <dbl> <dbl>
1 About eq… 227 7.11 4.66 0.309 6.51 7.72 6 1 25 6 5
2 Cat 656 5.23 3.03 0.118 4.99 5.46 5 0 15 3 4
3 Myself 242 8.63 4.95 0.318 8.00 9.26 8 0 25 5 7
# ℹ 1 more variable: UCImed <dbl>

#count of each range in a bar graph
g <-ggplot(data=fetch_recent_outliers_excl_end, aes(end_fetch))
g + geom_bar()


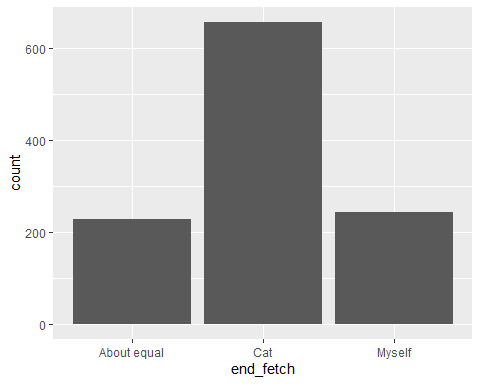


#outliers
#some outliers
ggplot(fetch_recent_outliers_excl_end, aes(x = end_fetch, y = times_object_retrieved_recent, fill = end_fetch)) +
 stat_boxplot(geom ="errorbar", width = 0.5) +
 geom_boxplot(fill = "light blue") +
 stat_summary(fun.y=mean, geom="point", shape=10, size=3.5, color="black") +
 ggtitle("Boxplots") +
 theme_bw() + theme(legend.position="none")


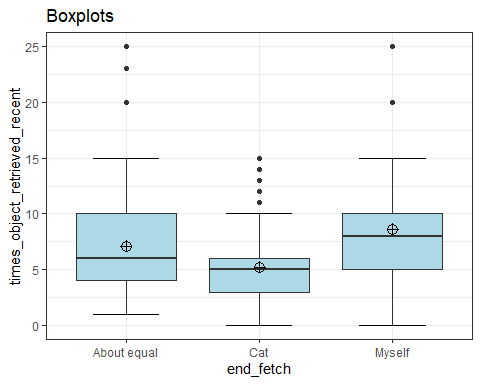


#identifies extreme outliers
#Values above Q3 + 3xIQR or below Q1 - 3xIQR are considered as extreme points (or extreme outliers)
#no extreme outliers anymore
fetch_recent_outliers_excl_end %>%
 group_by(end_fetch) %>%
 identify_outliers(times_object_retrieved_recent) %>%
 filter(is.extreme == "TRUE")

# A tibble: 0 × 4
# ℹ 4 variables: end_fetch <fct>, times_object_retrieved_recent <dbl>,
# is.outlier <lgl>, is.extreme <lgl>

#normality checks
#all significant results so data is not normally distributed
fetch_recent_outliers_excl_end %>%
 group_by(end_fetch) %>%
 summarise(`W Stat` = shapiro.test(times_object_retrieved_recent)$statistic,
 p.value = shapiro.test(times_object_retrieved_recent)$p.value)

# A tibble: 3 × 3
 end_fetch `W Stat` p.value
 <fct> <dbl> <dbl>
1 About equal 0.852 5.62e-14
2 Cat 0.872 1.00e-22
3 Myself 0.900 1.27e-11

#QQ plots
ggplot(data = fetch_recent_outliers_excl_end, mapping = aes(sample = times_object_retrieved_recent, color = end_fetch, fill = end_fetch)) +
 stat_qq_band(alpha=0.5, conf=0.95, qtype=1, bandType = "boot") +
 stat_qq_line(identity=TRUE) +
 stat_qq_point(col="black") +
 facet_wrap(~ end_fetch, scales = "free") +
 labs(x = "Theoretical Quantiles", y = "Sample Quantiles") + theme_bw()


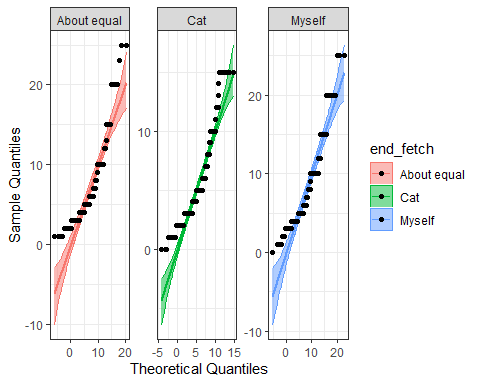


#so non-parametric tests are suitable

#Kruskal-Wallis test
#significant differences between groups
kruskal.test(times_object_retrieved_recent ~ end_fetch, data = fetch_recent_outliers_excl_end)

Kruskal-Wallis rank sum test

data: times_object_retrieved_recent by end_fetch
Kruskal-Wallis chi-squared = 121.69, df = 2, p-value < 2.2e-16

#Levene's test to test for equal variances
leveneTest(times_object_retrieved_recent ~ end_fetch, fetch_recent_outliers_excl_end)

Levene's Test for Homogeneity of Variance (center = median)
 Df F value Pr(>F)
group 2 38.763 < 2.2e-16 ***
 1122
---
Signif. codes: 0 '***' 0.001 '**' 0.01 '*' 0.05 '.' 0.1 ' ' 1

#significant so there are not equal variances = bonferroni is good alternative to Tukey

#pairwise comparisons using Bonferroni correction
pairwise.wilcox.test(fetch_recent_outliers_excl_end$times_object_retrieved_recent, fetch_recent_outliers_excl_end$end_fetch,
 p.adjust.method = "bonferroni")

Pairwise comparisons using Wilcoxon rank sum test with continuity correction

data: fetch_recent_outliers_excl_end$times_object_retrieved_recent and fetch_recent_outliers_excl_end$end_fetch

 About equal Cat
Cat 3.8e-08 -
Myself 0.00023 < 2e-16

P value adjustment method: bonferroni

#### No. retrievals x cat sex

#making appropriate dataset
fetch_sessions_recent_sex <- fetch_combined %>%
 select(cat_sex, times_object_retrieved_recent)

#ensuring as a factor
fetch_sessions_recent_sex$cat_sex<-as.factor(fetch_sessions_recent_sex$cat_sex)

#descriptive stats
fetch_sessions_recent_sex %>% select(cat_sex, times_object_retrieved_recent) %>% group_by(cat_sex) %>%
 summarise(n = n(),
 mean = mean(times_object_retrieved_recent, na.rm = TRUE),
 sd = sd(times_object_retrieved_recent, na.rm = TRUE),
 stderr = sd/sqrt(n),
 LCI = mean - qt(1 - (0.05 / 2), n - 1) * stderr,
 UCI = mean + qt(1 - (0.05 / 2), n - 1) * stderr,
 median = median(times_object_retrieved_recent, na.rm = TRUE),
 min = min(times_object_retrieved_recent, na.rm = TRUE),
 max = max(times_object_retrieved_recent, na.rm = TRUE),
 IQR = IQR(times_object_retrieved_recent, na.rm = TRUE),
 LCImed = MedianCI(times_object_retrieved_recent, na.rm=TRUE)[2],
 UCImed = MedianCI(times_object_retrieved_recent, na.rm=TRUE)[3])

# A tibble: 2 × 13
 cat_sex n mean sd stderr LCI UCI median min max IQR LCImed
 <fct> <int> <dbl> <dbl> <dbl> <dbl> <dbl> <dbl> <dbl> <dbl> <dbl> <dbl>
1 Female 537 6.81 6.09 0.263 6.30 7.33 5 0 68 5 5
2 Male 617 6.95 5.14 0.207 6.55 7.36 5 0 50 6 5
# ℹ 1 more variable: UCImed <dbl>

#count of each range in a bar graph in order
p <-ggplot(data=fetch_sessions_recent_sex, aes(cat_sex))
p + geom_bar()


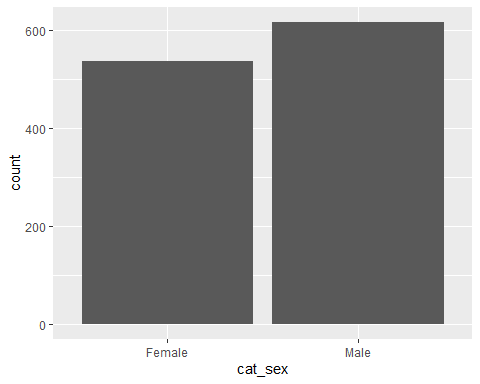


#outliers
#some outliers
ggplot(fetch_sessions_recent_sex, aes(x = cat_sex, y = times_object_retrieved_recent, fill = cat_sex)) +
 stat_boxplot(geom ="errorbar", width = 0.5) +
 geom_boxplot(fill = "light blue") +
 stat_summary(fun.y=mean, geom="point", shape=10, size=3.5, color="black") +
 ggtitle("Boxplots") +
 theme_bw() + theme(legend.position="none")


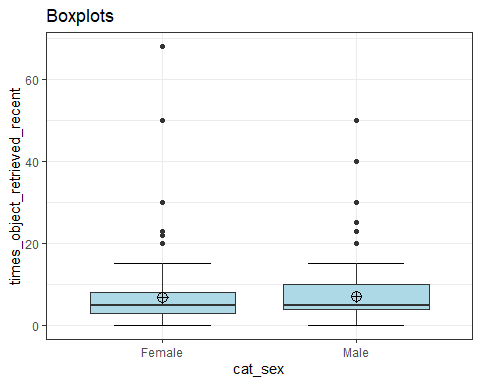


#identifies extreme outliers
#Values above Q3 + 3xIQR or below Q1 - 3xIQR are considered as extreme points (or extreme outliers)
fetch_sessions_recent_sex %>%
 group_by(cat_sex) %>%
 identify_outliers(times_object_retrieved_recent) %>%
 relocate(is.extreme) %>%
 filter(is.extreme == "TRUE")

# A tibble: 14 × 4
 is.extreme cat_sex times_object_retrieved_recent is.outlier
 <lgl> <fct> <dbl> <lgl>
 1 TRUE Female 30 TRUE
 2 TRUE Female 50 TRUE
 3 TRUE Female 68 TRUE
 4 TRUE Female 30 TRUE
 5 TRUE Female 30 TRUE
 6 TRUE Female 30 TRUE
 7 TRUE Female 30 TRUE
 8 TRUE Female 30 TRUE
 9 TRUE Female 30 TRUE
10 TRUE Female 30 TRUE
11 TRUE Female 30 TRUE
12 TRUE Male 30 TRUE
13 TRUE Male 40 TRUE
14 TRUE Male 50 TRUE

#normality checks
#all significant so data is not normally distributed
fetch_sessions_recent_sex %>%
 group_by(cat_sex) %>%
 summarise(`W Stat` = shapiro.test(times_object_retrieved_recent)$statistic,
 p.value = shapiro.test(times_object_retrieved_recent)$p.value)

# A tibble: 2 × 3
 cat_sex `W Stat` p.value
 <fct> <dbl> <dbl>
1 Female 0.664 3.50e-31
2 Male 0.785 1.01e-27

#QQ plots
#non-normal distributions
ggplot(data = fetch_sessions_recent_sex, mapping = aes(sample = times_object_retrieved_recent, color = cat_sex, fill = cat_sex)) +
 stat_qq_band(alpha=0.5, conf=0.95, qtype=1, bandType = "boot") +
 stat_qq_line(identity=TRUE) +
 stat_qq_point(col="black") +
 facet_wrap(~ cat_sex, scales = "free") +
 labs(x = "Theoretical Quantiles", y = "Sample Quantiles") + theme_bw()


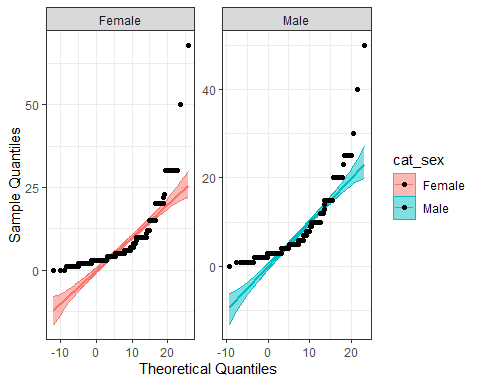


#so non-parametric tests are suitable

#non-significant differences between groups
mw <- wilcox.test(times_object_retrieved_recent ~ cat_sex, data=fetch_sessions_recent_sex, na.rm=TRUE, paired=FALSE, exact=FALSE, conf.int=TRUE)
print(mw)

Wilcoxon rank sum test with continuity correction

data: times_object_retrieved_recent by cat_sex
W = 156213, p-value = 0.09177
alternative hypothesis: true location shift is not equal to 0
95 percent confidence interval:
 -9.999190e-01 3.435625e-05
sample estimates:
difference in location
 -4.456412e-05

#z score of the converted W statistic
qnorm(mw$p.value/2)

[1] -1.686131

##### excluding extreme outliers

#data of only the extreme outliers
fetch_outliers_recent <- fetch_sessions_recent_sex %>%
 group_by(cat_sex) %>%
 identify_outliers(times_object_retrieved_recent) %>%
 filter(is.extreme == "TRUE")
fetch_outliers_recent

# A tibble: 14 × 4
 cat_sex times_object_retrieved_recent is.outlier is.extreme
 <fct> <dbl> <lgl> <lgl>
 1 Female 30 TRUE TRUE
 2 Female 50 TRUE TRUE
 3 Female 68 TRUE TRUE
 4 Female 30 TRUE TRUE
 5 Female 30 TRUE TRUE
 6 Female 30 TRUE TRUE
 7 Female 30 TRUE TRUE
 8 Female 30 TRUE TRUE
 9 Female 30 TRUE TRUE
10 Female 30 TRUE TRUE
11 Female 30 TRUE TRUE
12 Male 30 TRUE TRUE
13 Male 40 TRUE TRUE
14 Male 50 TRUE TRUE

#selecting columns
fetch_outliers_recent <- fetch_outliers_recent %>%
 select(cat_sex, times_object_retrieved_recent)

#then removing duplicates to exclude the extreme outliers from the dataset
fetch_recent_outliers_excl <- anti_join(fetch_sessions_recent_sex, fetch_outliers_recent)

#re-running analysis
#ensuring as a factor
fetch_recent_outliers_excl$cat_sex<-as.factor(fetch_recent_outliers_excl$cat_sex)

#descriptive stats
fetch_recent_outliers_excl %>% select(cat_sex, times_object_retrieved_recent) %>% group_by(cat_sex) %>%
 summarise(n = n(),
 mean = mean(times_object_retrieved_recent, na.rm = TRUE),
 sd = sd(times_object_retrieved_recent, na.rm = TRUE),
 stderr = sd/sqrt(n),
 LCI = mean - qt(1 - (0.05 / 2), n - 1) * stderr,
 UCI = mean + qt(1 - (0.05 / 2), n - 1) * stderr,
 median = median(times_object_retrieved_recent, na.rm = TRUE),
 min = min(times_object_retrieved_recent, na.rm = TRUE),
 max = max(times_object_retrieved_recent, na.rm = TRUE),
 IQR = IQR(times_object_retrieved_recent, na.rm = TRUE),
 LCImed = MedianCI(times_object_retrieved_recent, na.rm=TRUE)[2],
 UCImed = MedianCI(times_object_retrieved_recent, na.rm=TRUE)[3])

# A tibble: 2 × 13
 cat_sex n mean sd stderr LCI UCI median min max IQR LCImed
 <fct> <int> <dbl> <dbl> <dbl> <dbl> <dbl> <dbl> <dbl> <dbl> <dbl> <dbl>
1 Female 526 6.22 4.19 0.183 5.86 6.58 5 0 23 5 5
2 Male 614 6.79 4.57 0.184 6.43 7.16 5 0 25 5.75 5
# ℹ 1 more variable: UCImed <dbl>

#count of each range in a bar graph in order
p <-ggplot(data=fetch_recent_outliers_excl, aes(cat_sex))
p + geom_bar()


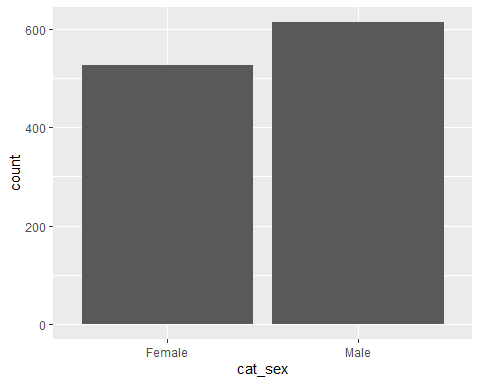


#outliers
#some outliers
ggplot(fetch_recent_outliers_excl, aes(x = cat_sex, y = times_object_retrieved_recent, fill = cat_sex)) +
 stat_boxplot(geom ="errorbar", width = 0.5) +
 geom_boxplot(fill = "light blue") +
 stat_summary(fun.y=mean, geom="point", shape=10, size=3.5, color="black") +
 ggtitle("Boxplots") +
 theme_bw() + theme(legend.position="none")


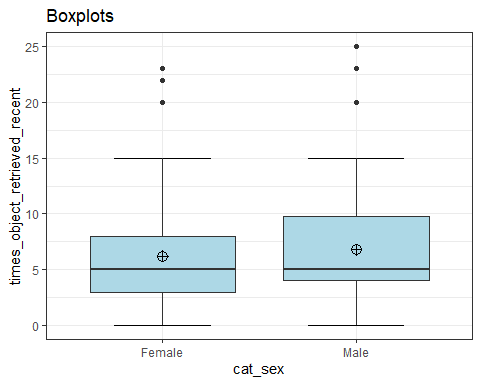


#identifies extreme outliers
#Values above Q3 + 3xIQR or below Q1 - 3xIQR are considered as extreme points (or extreme outliers)
#no extreme outliers
fetch_recent_outliers_excl %>%
 group_by(cat_sex) %>%
 identify_outliers(times_object_retrieved_recent) %>%
 relocate(is.extreme) %>%
 filter(is.extreme == "TRUE")

# A tibble: 0 × 4
# ℹ 4 variables: is.extreme <lgl>, cat_sex <fct>,
# times_object_retrieved_recent <dbl>, is.outlier <lgl>

#normality checks
#all significant so data is not normally distributed
fetch_recent_outliers_excl %>%
 group_by(cat_sex) %>%
 summarise(`W Stat` = shapiro.test(times_object_retrieved_recent)$statistic,
 p.value = shapiro.test(times_object_retrieved_recent)$p.value)

# A tibble: 2 × 3
 cat_sex `W Stat` p.value
 <fct> <dbl> <dbl>
1 Female 0.829 2.68e-23
2 Male 0.848 7.62e-24

#QQ plots
#non-normal distributions
ggplot(data = fetch_recent_outliers_excl, mapping = aes(sample = times_object_retrieved_recent, color = cat_sex, fill = cat_sex)) +
 stat_qq_band(alpha=0.5, conf=0.95, qtype=1, bandType = "boot") +
 stat_qq_line(identity=TRUE) +
 stat_qq_point(col="black") +
 facet_wrap(~ cat_sex, scales = "free") +
 labs(x = "Theoretical Quantiles", y = "Sample Quantiles") + theme_bw()


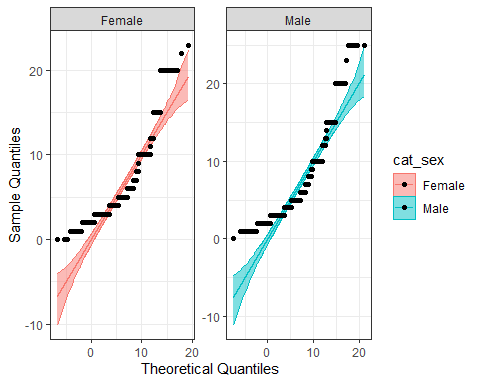


#so non-parametric tests are suitable

#significant differences between groups
mw <- wilcox.test(times_object_retrieved_recent ~ cat_sex, data=fetch_recent_outliers_excl, na.rm=TRUE, paired=FALSE, exact=FALSE, conf.int=TRUE)
print(mw)

Wilcoxon rank sum test with continuity correction

data: times_object_retrieved_recent by cat_sex
W = 149449, p-value = 0.02865
alternative hypothesis: true location shift is not equal to 0
95 percent confidence interval:
 -9.999587e-01 -7.196151e-06
sample estimates:
difference in location
 -6.178306e-05

#Hodges-Lehmann estimate
mw$estimate

difference in location
 -6.178306e-05

### D. Monthly object retrievals

#### No. monthly sessions x initiators

#making appropriate dataset
fetch_sessions_monthly <- fetch_combined %>%
 select(initiate_fetch, times_monthly_fetch_sessions)

#ensuring as a factor
fetch_sessions_monthly$initiate_fetch<-as.factor(fetch_sessions_monthly$initiate_fetch)

#descriptive stats
fetch_sessions_monthly %>% select(initiate_fetch, times_monthly_fetch_sessions) %>% group_by(initiate_fetch) %>%
 summarise(n = n(),
 mean = mean(times_monthly_fetch_sessions, na.rm = TRUE),
 sd = sd(times_monthly_fetch_sessions, na.rm = TRUE),
 stderr = sd/sqrt(n),
 LCI = mean - qt(1 - (0.05 / 2), n - 1) * stderr,
 UCI = mean + qt(1 - (0.05 / 2), n - 1) * stderr,
 median = median(times_monthly_fetch_sessions, na.rm = TRUE),
 min = min(times_monthly_fetch_sessions, na.rm = TRUE),
 max = max(times_monthly_fetch_sessions, na.rm = TRUE),
 IQR = IQR(times_monthly_fetch_sessions, na.rm = TRUE),
 LCImed = MedianCI(times_monthly_fetch_sessions, na.rm=TRUE)[2],
 UCImed = MedianCI(times_monthly_fetch_sessions, na.rm=TRUE)[3])

# A tibble: 3 × 13
 initiate_fetch n mean sd stderr LCI UCI median min max IQR
 <fct> <int> <dbl> <dbl> <dbl> <dbl> <dbl> <dbl> <dbl> <dbl> <dbl>
1 About equal 352 12.7 13.2 0.705 11.3 14.1 10 0 100 16
2 Cat 553 14.7 16.7 0.710 13.3 16.1 10 0 180 16
3 Myself 249 8.30 9.52 0.603 7.11 9.49 4 0 60 8
# ℹ 2 more variables: LCImed <dbl>, UCImed <dbl>

#count of each range in a bar graph in order
p <-ggplot(data=fetch_sessions_monthly, aes(initiate_fetch))
p + geom_bar()


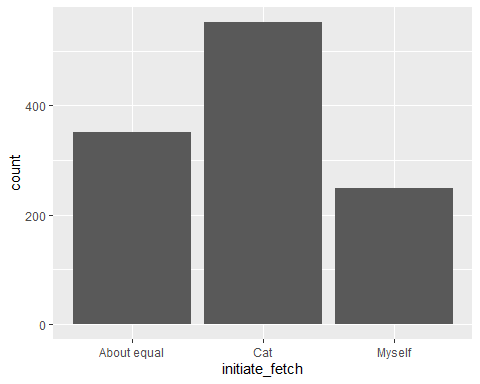


#outliers
#some outliers
ggplot(fetch_sessions_monthly, aes(x = initiate_fetch, y = times_monthly_fetch_sessions, fill = initiate_fetch)) +
 stat_boxplot(geom ="errorbar", width = 0.5) +
 geom_boxplot(fill = "light blue") +
 stat_summary(fun.y=mean, geom="point", shape=10, size=3.5, color="black") +
 ggtitle("Boxplots") +
 theme_bw() + theme(legend.position="none")


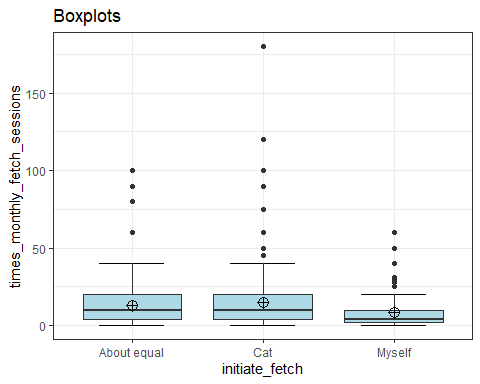


#identifies extreme outliers
#Values above Q3 + 3xIQR or below Q1 - 3xIQR are considered as extreme points (or extreme outliers)
fetch_sessions_monthly %>%
 group_by(initiate_fetch) %>%
 identify_outliers(times_monthly_fetch_sessions) %>%
 relocate(is.extreme) %>%
 filter(is.extreme == "TRUE")

# A tibble: 16 × 4
 is.extreme initiate_fetch times_monthly_fetch_sessions is.outlier
 <lgl> <fct> <dbl> <lgl>
 1 TRUE About equal 80 TRUE
 2 TRUE About equal 90 TRUE
 3 TRUE About equal 90 TRUE
 4 TRUE About equal 100 TRUE
 5 TRUE Cat 100 TRUE
 6 TRUE Cat 75 TRUE
 7 TRUE Cat 120 TRUE
 8 TRUE Cat 90 TRUE
 9 TRUE Cat 180 TRUE
10 TRUE Cat 100 TRUE
11 TRUE Cat 75 TRUE
12 TRUE Cat 100 TRUE
13 TRUE Myself 40 TRUE
14 TRUE Myself 60 TRUE
15 TRUE Myself 50 TRUE
16 TRUE Myself 40 TRUE

#normality checks
#all significant so data is not normally distributed
fetch_sessions_monthly %>%
 group_by(initiate_fetch) %>%
 summarise(`W Stat` = shapiro.test(times_monthly_fetch_sessions)$statistic,
 p.value = shapiro.test(times_monthly_fetch_sessions)$p.value)

# A tibble: 3 × 3
 initiate_fetch `W Stat` p.value
 <fct> <dbl> <dbl>
1 About equal 0.732 2.00e-23
2 Cat 0.692 1.54e-30
3 Myself 0.775 3.42e-18

#QQ plots
#non-normal distributions
ggplot(data = fetch_sessions_monthly, mapping = aes(sample = times_monthly_fetch_sessions, color = initiate_fetch, fill = initiate_fetch)) +
 stat_qq_band(alpha=0.5, conf=0.95, qtype=1, bandType = "boot") +
 stat_qq_line(identity=TRUE) +
 stat_qq_point(col="black") +
 facet_wrap(~ initiate_fetch, scales = "free") +
 labs(x = "Theoretical Quantiles", y = "Sample Quantiles") + theme_bw()


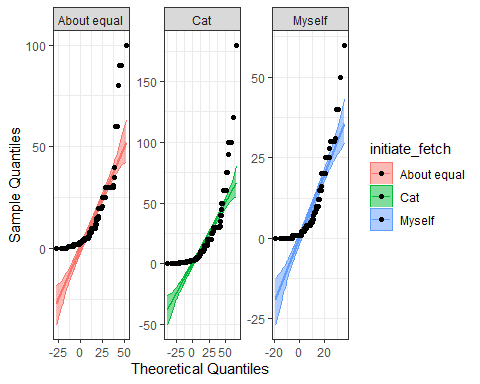


#so non-parametric tests are suitable

#Kruskal-Wallis test
#significant differences between groups
kruskal.test(times_monthly_fetch_sessions ~ initiate_fetch, data = fetch_sessions_monthly)

Kruskal-Wallis rank sum test

data: times_monthly_fetch_sessions by initiate_fetch
Kruskal-Wallis chi-squared = 52.947, df = 2, p-value = 3.182e-12

#Levene's test to test for equal variances
leveneTest(times_monthly_fetch_sessions ~ initiate_fetch, fetch_sessions_monthly)

Levene's Test for Homogeneity of Variance (center = median)
 Df F value Pr(>F)
group 2 9.8821 5.555e-05 ***
 1151
---
Signif. codes: 0 '***' 0.001 '**' 0.01 '*' 0.05 '.' 0.1 ' ' 1

#significant so there are not equal variances = bonferroni is good alternative to Tukey

#pairwise comparisons using Bonferroni correction
pairwise.wilcox.test(fetch_sessions_monthly$times_monthly_fetch_sessions, fetch_sessions_monthly$initiate_fetch,
 p.adjust.method = "bonferroni")

Pairwise comparisons using Wilcoxon rank sum test with continuity correction

data: fetch_sessions_monthly$times_monthly_fetch_sessions and fetch_sessions_monthly$initiate_fetch

 About equal Cat
Cat 0.66 -
Myself 1.2e-08 9.4e-12

P value adjustment method: bonferroni

##### excluding extreme outliers

#data of only the extreme outliers
fetch_outliers_monthly <- fetch_sessions_monthly %>%
 group_by(initiate_fetch) %>%
 identify_outliers(times_monthly_fetch_sessions) %>%
 filter(is.extreme == "TRUE")
fetch_outliers_monthly

# A tibble: 16 × 4
 initiate_fetch times_monthly_fetch_sessions is.outlier is.extreme
 <fct> <dbl> <lgl> <lgl>
 1 About equal 80 TRUE TRUE
 2 About equal 90 TRUE TRUE
 3 About equal 90 TRUE TRUE
 4 About equal 100 TRUE TRUE
 5 Cat 100 TRUE TRUE
 6 Cat 75 TRUE TRUE
 7 Cat 120 TRUE TRUE
 8 Cat 90 TRUE TRUE
 9 Cat 180 TRUE TRUE
10 Cat 100 TRUE TRUE
11 Cat 75 TRUE TRUE
12 Cat 100 TRUE TRUE
13 Myself 40 TRUE TRUE
14 Myself 60 TRUE TRUE
15 Myself 50 TRUE TRUE
16 Myself 40 TRUE TRUE

#selecting columns
fetch_outliers_monthly <- fetch_outliers_monthly %>%
 select(initiate_fetch, times_monthly_fetch_sessions)

#then removing duplicates to exclude the extreme outliers from the dataset
fetch_recent_outliers_excl <- anti_join(fetch_sessions_monthly, fetch_outliers_monthly)

#re-running same analysis to see if outliers affect significance
#ensuring as a factor
fetch_recent_outliers_excl$initiate_fetch<-as.factor(fetch_recent_outliers_excl$initiate_fetch)

#descriptive stats
fetch_recent_outliers_excl %>% select(initiate_fetch, times_monthly_fetch_sessions) %>% group_by(initiate_fetch) %>%
 summarise(n = n(),
 mean = mean(times_monthly_fetch_sessions, na.rm = TRUE),
 sd = sd(times_monthly_fetch_sessions, na.rm = TRUE),
 stderr = sd/sqrt(n),
 LCI = mean - qt(1 - (0.05 / 2), n - 1) * stderr,
 UCI = mean + qt(1 - (0.05 / 2), n - 1) * stderr,
 median = median(times_monthly_fetch_sessions, na.rm = TRUE),
 min = min(times_monthly_fetch_sessions, na.rm = TRUE),
 max = max(times_monthly_fetch_sessions, na.rm = TRUE),
 IQR = IQR(times_monthly_fetch_sessions, na.rm = TRUE),
 LCImed = MedianCI(times_monthly_fetch_sessions, na.rm=TRUE)[2],
 UCImed = MedianCI(times_monthly_fetch_sessions, na.rm=TRUE)[3])

# A tibble: 3 × 13
 initiate_fetch n mean sd stderr LCI UCI median min max IQR
 <fct> <int> <dbl> <dbl> <dbl> <dbl> <dbl> <dbl> <dbl> <dbl> <dbl>
1 About equal 348 11.8 10.3 0.554 10.7 12.9 9.5 0 60 16
2 Cat 545 13.4 12.1 0.518 12.3 14.4 10 0 60 16
3 Myself 245 7.66 8.09 0.517 6.64 8.68 4 0 31 8
# ℹ 2 more variables: LCImed <dbl>, UCImed <dbl>

#count of each range in a bar graph
g <-ggplot(data=fetch_recent_outliers_excl, aes(initiate_fetch))
g + geom_bar()


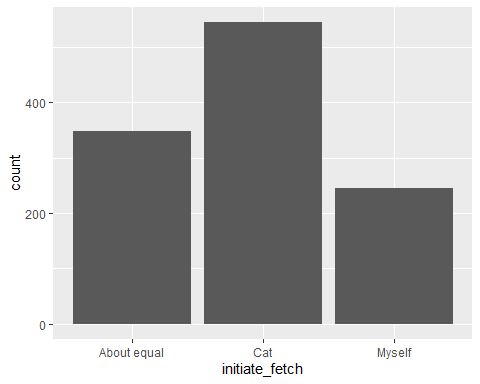


#outliers
#some outliers
ggplot(fetch_recent_outliers_excl, aes(x = initiate_fetch, y = times_monthly_fetch_sessions, fill = initiate_fetch)) +
 stat_boxplot(geom ="errorbar", width = 0.5) +
 geom_boxplot(fill = "light blue") +
 stat_summary(fun.y=mean, geom="point", shape=10, size=3.5, color="black") +
 ggtitle("Boxplots") +
 theme_bw() + theme(legend.position="none")


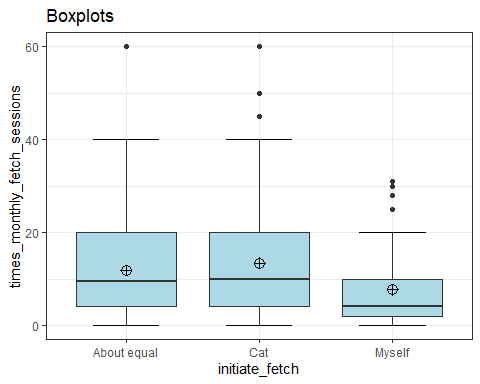


#identifies extreme outliers
#Values above Q3 + 3xIQR or below Q1 - 3xIQR are considered as extreme points (or extreme outliers)
#no extreme outliers anymore
fetch_recent_outliers_excl %>%
 group_by(initiate_fetch) %>%
 identify_outliers(times_monthly_fetch_sessions) %>%
 filter(is.extreme == "TRUE")

# A tibble: 0 × 4
# ℹ 4 variables: initiate_fetch <fct>, times_monthly_fetch_sessions <dbl>,
# is.outlier <lgl>, is.extreme <lgl>

#normality checks
#all significant results so data is not normally distributed
fetch_recent_outliers_excl %>%
 group_by(initiate_fetch) %>%
 summarise(`W Stat` = shapiro.test(times_monthly_fetch_sessions)$statistic,
 p.value = shapiro.test(times_monthly_fetch_sessions)$p.value)

# A tibble: 3 × 3
 initiate_fetch `W Stat` p.value
 <fct> <dbl> <dbl>
1 About equal 0.851 1.10e-17
2 Cat 0.853 4.12e-22
3 Myself 0.810 1.36e-16

#QQ plots
ggplot(data = fetch_recent_outliers_excl, mapping = aes(sample = times_monthly_fetch_sessions, color = initiate_fetch, fill = initiate_fetch)) +
 stat_qq_band(alpha=0.5, conf=0.95, qtype=1, bandType = "boot") +
 stat_qq_line(identity=TRUE) +
 stat_qq_point(col="black") +
 facet_wrap(~ initiate_fetch, scales = "free") +
 labs(x = "Theoretical Quantiles", y = "Sample Quantiles") + theme_bw()


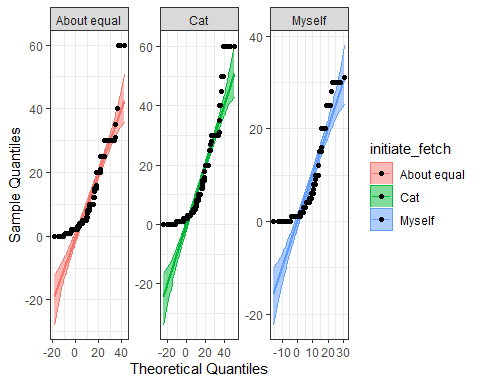


#so non-parametric tests are suitable

#Kruskal-Wallis test
#significant differences between groups
kruskal.test(times_monthly_fetch_sessions ~ initiate_fetch, data = fetch_recent_outliers_excl)

Kruskal-Wallis rank sum test

data: times_monthly_fetch_sessions by initiate_fetch
Kruskal-Wallis chi-squared = 55.882, df = 2, p-value = 7.333e-13

#Levene's test to test for equal variances
leveneTest(times_monthly_fetch_sessions ~ initiate_fetch, fetch_recent_outliers_excl)

Levene's Test for Homogeneity of Variance (center = median)
 Df F value Pr(>F)
group 2 15.51 2.262e-07 ***
 1135
---
Signif. codes: 0 '***' 0.001 '**' 0.01 '*' 0.05 '.' 0.1 ' ' 1

#significant so there are not equal variances = bonferroni is good alternative to Tukey

#pairwise comparisons using Bonferroni correction
pairwise.wilcox.test(fetch_recent_outliers_excl$times_monthly_fetch_sessions, fetch_recent_outliers_excl$initiate_fetch,
 p.adjust.method = "bonferroni")

Pairwise comparisons using Wilcoxon rank sum test with continuity correction

data: fetch_recent_outliers_excl$times_monthly_fetch_sessions and fetch_recent_outliers_excl$initiate_fetch

 About equal Cat
Cat 0.73 -
Myself 3.3e-09 2.6e-12

P value adjustment method: bonferroni

#### No. monthly sessions x ended

#making appropriate dataset
fetch_sessions_monthly_end <- fetch_combined %>%
 select(end_fetch, times_monthly_fetch_sessions)

#ensuring as a factor
fetch_sessions_monthly_end$end_fetch<-as.factor(fetch_sessions_monthly_end$end_fetch)

#descriptive stats
fetch_sessions_monthly_end %>% select(end_fetch, times_monthly_fetch_sessions) %>% group_by(end_fetch) %>%
 summarise(n = n(),
 mean = mean(times_monthly_fetch_sessions, na.rm = TRUE),
 sd = sd(times_monthly_fetch_sessions, na.rm = TRUE),
 stderr = sd/sqrt(n),
 LCI = mean - qt(1 - (0.05 / 2), n - 1) * stderr,
 UCI = mean + qt(1 - (0.05 / 2), n - 1) * stderr,
 median = median(times_monthly_fetch_sessions, na.rm = TRUE),
 min = min(times_monthly_fetch_sessions, na.rm = TRUE),
 max = max(times_monthly_fetch_sessions, na.rm = TRUE),
 IQR = IQR(times_monthly_fetch_sessions, na.rm = TRUE),
 LCImed = MedianCI(times_monthly_fetch_sessions, na.rm=TRUE)[2],
 UCImed = MedianCI(times_monthly_fetch_sessions, na.rm=TRUE)[3])

# A tibble: 3 × 13
 end_fetch n mean sd stderr LCI UCI median min max IQR LCImed
 <fct> <int> <dbl> <dbl> <dbl> <dbl> <dbl> <dbl> <dbl> <dbl> <dbl> <dbl>
1 About eq… 231 15.6 14.6 0.964 13.7 17.5 12 0 100 20 10
2 Cat 675 10.4 11.9 0.460 9.49 11.3 6 0 120 12.5 5
3 Myself 248 16.3 19.1 1.21 13.9 18.7 10 0 180 15 10
# ℹ 1 more variable: UCImed <dbl>

#count of each range in a bar graph in order
p <-ggplot(data=fetch_sessions_monthly_end, aes(end_fetch))
p + geom_bar()


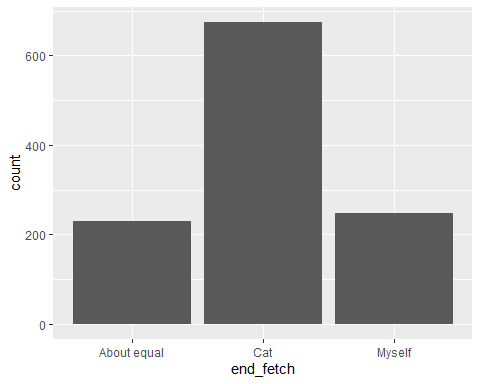


#outliers
#some outliers
ggplot(fetch_sessions_monthly_end, aes(x = end_fetch, y = times_monthly_fetch_sessions, fill = end_fetch)) +
 stat_boxplot(geom ="errorbar", width = 0.5) +
 geom_boxplot(fill = "light blue") +
 stat_summary(fun.y=mean, geom="point", shape=10, size=3.5, color="black") +
 ggtitle("Boxplots") +
 theme_bw() + theme(legend.position="none")


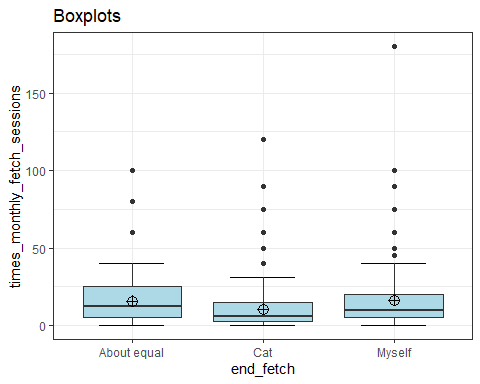


#identifies extreme outliers
#Values above Q3 + 3xIQR or below Q1 - 3xIQR are considered as extreme points (or extreme outliers)
fetch_sessions_monthly_end %>%
 group_by(end_fetch) %>%
 identify_outliers(times_monthly_fetch_sessions) %>%
 relocate(is.extreme) %>%
 filter(is.extreme == "TRUE")

# A tibble: 16 × 4
 is.extreme end_fetch times_monthly_fetch_sessions is.outlier
 <lgl> <fct> <dbl> <lgl>
 1 TRUE About equal 100 TRUE
 2 TRUE About equal 100 TRUE
 3 TRUE Cat 60 TRUE
 4 TRUE Cat 60 TRUE
 5 TRUE Cat 120 TRUE
 6 TRUE Cat 60 TRUE
 7 TRUE Cat 60 TRUE
 8 TRUE Cat 90 TRUE
 9 TRUE Cat 75 TRUE
10 TRUE Cat 60 TRUE
11 TRUE Myself 100 TRUE
12 TRUE Myself 75 TRUE
13 TRUE Myself 90 TRUE
14 TRUE Myself 90 TRUE
15 TRUE Myself 180 TRUE
16 TRUE Myself 100 TRUE

#normality checks
#all significant so data is not normally distributed
fetch_sessions_monthly_end %>%
 group_by(end_fetch) %>%
 summarise(`W Stat` = shapiro.test(times_monthly_fetch_sessions)$statistic,
 p.value = shapiro.test(times_monthly_fetch_sessions)$p.value)

# A tibble: 3 × 3
 end_fetch `W Stat` p.value
 <fct> <dbl> <dbl>
1 About equal 0.772 1.28e-17
2 Cat 0.733 1.69e-31
3 Myself 0.648 2.50e-22

#QQ plots
#non-normal distributions
ggplot(data = fetch_sessions_monthly_end, mapping = aes(sample = times_monthly_fetch_sessions, color = end_fetch, fill = end_fetch)) +
 stat_qq_band(alpha=0.5, conf=0.95, qtype=1, bandType = "boot") +
 stat_qq_line(identity=TRUE) +
 stat_qq_point(col="black") +
 facet_wrap(~ end_fetch, scales = "free") +
 labs(x = "Theoretical Quantiles", y = "Sample Quantiles") + theme_bw()


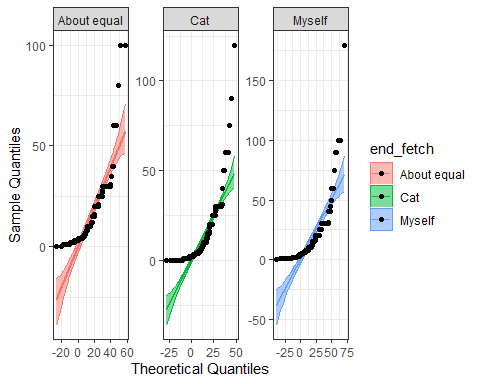


#so non-parametric tests are suitable

#Kruskal-Wallis test
#significant differences between groups
kruskal.test(times_monthly_fetch_sessions ~ end_fetch, data = fetch_sessions_monthly_end)

Kruskal-Wallis rank sum test

data: times_monthly_fetch_sessions by end_fetch
Kruskal-Wallis chi-squared = 62.972, df = 2, p-value = 2.117e-14

#Levene's test to test for equal variances
leveneTest(times_monthly_fetch_sessions ~ end_fetch, fetch_sessions_monthly_end)

Levene's Test for Homogeneity of Variance (center = median)
 Df F value Pr(>F)
group 2 8.0601 0.0003341 ***
 1151
---
Signif. codes: 0 '***' 0.001 '**' 0.01 '*' 0.05 '.' 0.1 ' ' 1

#significant so there are not equal variances = bonferroni is good alternative to Tukey

#pairwise comparisons using Bonferroni correction
pairwise.wilcox.test(fetch_sessions_monthly_end$times_monthly_fetch_sessions, fetch_sessions_monthly_end$end_fetch,
 p.adjust.method = "bonferroni")

Pairwise comparisons using Wilcoxon rank sum test with continuity correction

data: fetch_sessions_monthly_end$times_monthly_fetch_sessions and fetch_sessions_monthly_end$end_fetch

 About equal Cat
Cat 3.0e-10 -
Myself 1 2.6e-09

P value adjustment method: bonferroni

##### excluding extreme outliers

#data of only the extreme outliers
fetch_outliers_monthly_end <- fetch_sessions_monthly_end %>%
 group_by(end_fetch) %>%
 identify_outliers(times_monthly_fetch_sessions) %>%
 filter(is.extreme == "TRUE")
fetch_outliers_monthly_end

# A tibble: 16 × 4
 end_fetch times_monthly_fetch_sessions is.outlier is.extreme
 <fct> <dbl> <lgl> <lgl>
 1 About equal 100 TRUE TRUE
 2 About equal 100 TRUE TRUE
 3 Cat 60 TRUE TRUE
 4 Cat 60 TRUE TRUE
 5 Cat 120 TRUE TRUE
 6 Cat 60 TRUE TRUE
 7 Cat 60 TRUE TRUE
 8 Cat 90 TRUE TRUE
 9 Cat 75 TRUE TRUE
10 Cat 60 TRUE TRUE
11 Myself 100 TRUE TRUE
12 Myself 75 TRUE TRUE
13 Myself 90 TRUE TRUE
14 Myself 90 TRUE TRUE
15 Myself 180 TRUE TRUE
16 Myself 100 TRUE TRUE

#selecting columns
fetch_outliers_monthly_end <- fetch_outliers_monthly_end %>%
 select(end_fetch, times_monthly_fetch_sessions)

#then removing duplicates to exclude the extreme outliers from the dataset
fetch_recent_outliers_excl_end <- anti_join(fetch_sessions_monthly_end, fetch_outliers_monthly_end)

#re-running same analysis to see if outliers affect significance
#ensuring as a factor
fetch_recent_outliers_excl_end$end_fetch<-as.factor(fetch_recent_outliers_excl_end$end_fetch)

#descriptive stats
fetch_recent_outliers_excl_end %>% select(end_fetch, times_monthly_fetch_sessions) %>% group_by(end_fetch) %>%
 summarise(n = n(),
 mean = mean(times_monthly_fetch_sessions, na.rm = TRUE),
 sd = sd(times_monthly_fetch_sessions, na.rm = TRUE),
 stderr = sd/sqrt(n),
 LCI = mean - qt(1 - (0.05 / 2), n - 1) * stderr,
 UCI = mean + qt(1 - (0.05 / 2), n - 1) * stderr,
 median = median(times_monthly_fetch_sessions, na.rm = TRUE),
 min = min(times_monthly_fetch_sessions, na.rm = TRUE),
 max = max(times_monthly_fetch_sessions, na.rm = TRUE),
 IQR = IQR(times_monthly_fetch_sessions, na.rm = TRUE),
 LCImed = MedianCI(times_monthly_fetch_sessions, na.rm=TRUE)[2],
 UCImed = MedianCI(times_monthly_fetch_sessions, na.rm=TRUE)[3])

# A tibble: 3 × 13
 end_fetch n mean sd stderr LCI UCI median min max IQR LCImed
 <fct> <int> <dbl> <dbl> <dbl> <dbl> <dbl> <dbl> <dbl> <dbl> <dbl> <dbl>
1 About eq… 229 14.8 12.4 0.818 13.2 16.5 10 0 80 16 10
2 Cat 667 9.64 9.57 0.371 8.91 10.4 5 0 50 13 5
3 Myself 242 14.1 11.9 0.765 12.6 15.6 10 0 60 15 10
# ℹ 1 more variable: UCImed <dbl>

#count of each range in a bar graph
g <-ggplot(data=fetch_recent_outliers_excl_end, aes(end_fetch))
g + geom_bar()


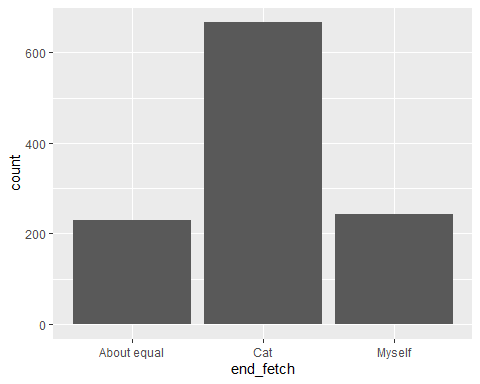


#outliers
#some outliers
ggplot(fetch_recent_outliers_excl_end, aes(x = end_fetch, y = times_monthly_fetch_sessions, fill = end_fetch)) +
 stat_boxplot(geom ="errorbar", width = 0.5) +
 geom_boxplot(fill = "light blue") +
 stat_summary(fun.y=mean, geom="point", shape=10, size=3.5, color="black") +
 ggtitle("Boxplots") +
 theme_bw() + theme(legend.position="none")


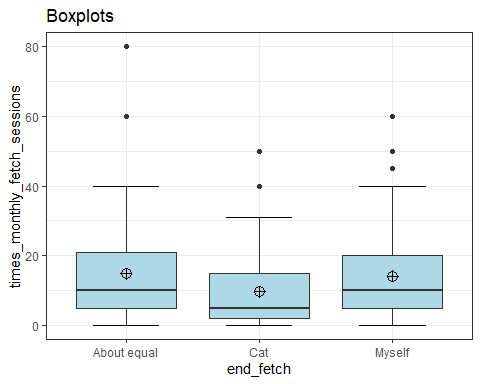


#identifies extreme outliers
#Values above Q3 + 3xIQR or below Q1 - 3xIQR are considered as extreme points (or extreme outliers)
#1 extreme outlier further identified
fetch_recent_outliers_excl_end %>%
 group_by(end_fetch) %>%
 identify_outliers(times_monthly_fetch_sessions) %>%
 filter(is.extreme == "TRUE")

# A tibble: 1 × 4
 end_fetch times_monthly_fetch_sessions is.outlier is.extreme
 <fct> <dbl> <lgl> <lgl>
1 About equal 80 TRUE TRUE

#excluding 1 further extreme outlier
fetch_recent_outliers_excl_end <- fetch_recent_outliers_excl_end %>%
 filter(!(end_fetch == "About equal" & times_monthly_fetch_sessions == "80"))

#checking for extreme outliers again
#all good!
fetch_recent_outliers_excl_end %>%
 group_by(end_fetch) %>%
 identify_outliers(times_monthly_fetch_sessions) %>%
 filter(is.extreme == "TRUE")

# A tibble: 0 × 4
# ℹ 4 variables: end_fetch <fct>, times_monthly_fetch_sessions <dbl>,
# is.outlier <lgl>, is.extreme <lgl>

#descriptive stats again with extreme outlier removed
#descriptive stats
fetch_recent_outliers_excl_end %>% select(end_fetch, times_monthly_fetch_sessions) %>% group_by(end_fetch) %>%
 summarise(n = n(),
 mean = mean(times_monthly_fetch_sessions, na.rm = TRUE),
 sd = sd(times_monthly_fetch_sessions, na.rm = TRUE),
 stderr = sd/sqrt(n),
 LCI = mean - qt(1 - (0.05 / 2), n - 1) * stderr,
 UCI = mean + qt(1 - (0.05 / 2), n - 1) * stderr,
 median = median(times_monthly_fetch_sessions, na.rm = TRUE),
 min = min(times_monthly_fetch_sessions, na.rm = TRUE),
 max = max(times_monthly_fetch_sessions, na.rm = TRUE),
 IQR = IQR(times_monthly_fetch_sessions, na.rm = TRUE),
 LCImed = MedianCI(times_monthly_fetch_sessions, na.rm=TRUE)[2],
 UCImed = MedianCI(times_monthly_fetch_sessions, na.rm=TRUE)[3])

# A tibble: 3 × 13
 end_fetch n mean sd stderr LCI UCI median min max IQR LCImed
 <fct> <int> <dbl> <dbl> <dbl> <dbl> <dbl> <dbl> <dbl> <dbl> <dbl> <dbl>
1 About eq… 228 14.6 11.6 0.770 13.0 16.1 10 0 60 15.2 10
2 Cat 667 9.64 9.57 0.371 8.91 10.4 5 0 50 13 5
3 Myself 242 14.1 11.9 0.765 12.6 15.6 10 0 60 15 10
# ℹ 1 more variable: UCImed <dbl>

#normality checks
#all significant results so data is not normally distributed
fetch_recent_outliers_excl_end %>%
 group_by(end_fetch) %>%
 summarise(`W Stat` = shapiro.test(times_monthly_fetch_sessions)$statistic,
 p.value = shapiro.test(times_monthly_fetch_sessions)$p.value)

# A tibble: 3 × 3
 end_fetch `W Stat` p.value
 <fct> <dbl> <dbl>
1 About equal 0.878 1.49e-12
2 Cat 0.832 7.14e-26
3 Myself 0.866 9.99e-14

#QQ plots
ggplot(data = fetch_recent_outliers_excl_end, mapping = aes(sample = times_monthly_fetch_sessions, color = end_fetch, fill = end_fetch)) +
 stat_qq_band(alpha=0.5, conf=0.95, qtype=1, bandType = "boot") +
 stat_qq_line(identity=TRUE) +
 stat_qq_point(col="black") +
 facet_wrap(~ end_fetch, scales = "free") +
 labs(x = "Theoretical Quantiles", y = "Sample Quantiles") + theme_bw()


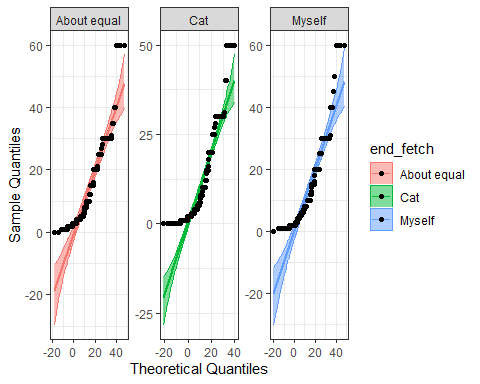


#so non-parametric tests are suitable

#Kruskal-Wallis test
#significant differences between groups
kruskal.test(times_monthly_fetch_sessions ~ end_fetch, data = fetch_recent_outliers_excl_end)

Kruskal-Wallis rank sum test

data: times_monthly_fetch_sessions by end_fetch
Kruskal-Wallis chi-squared = 62.687, df = 2, p-value = 2.442e-14

#Levene's test to test for equal variances
leveneTest(times_monthly_fetch_sessions ~ end_fetch, fetch_recent_outliers_excl_end)

Levene's Test for Homogeneity of Variance (center = median)
 Df F value Pr(>F)
group 2 8.0976 0.0003222 ***
 1134
---
Signif. codes: 0 '***' 0.001 '**' 0.01 '*' 0.05 '.' 0.1 ' ' 1

#significant so there are not equal variances = bonferroni is good alternative to Tukey

#pairwise comparisons using Bonferroni correction
pairwise.wilcox.test(fetch_recent_outliers_excl_end$times_monthly_fetch_sessions, fetch_recent_outliers_excl_end$end_fetch,
 p.adjust.method = "bonferroni")

Pairwise comparisons using Wilcoxon rank sum test with continuity correction

data: fetch_recent_outliers_excl_end$times_monthly_fetch_sessions and fetch_recent_outliers_excl_end$end_fetch

 About equal Cat
Cat 1.7e-10 -
Myself 1 6.3e-09

P value adjustment method: bonferroni

#### No. monthly sessions x cat sex

#making appropriate dataset
fetch_sessions_monthly_sex <- fetch_combined %>%
 select(cat_sex, times_monthly_fetch_sessions)

#ensuring as a factor
fetch_sessions_monthly_sex$cat_sex<-as.factor(fetch_sessions_monthly_sex$cat_sex)

#descriptive stats
fetch_sessions_monthly_sex %>% select(cat_sex, times_monthly_fetch_sessions) %>% group_by(cat_sex) %>%
 summarise(n = n(),
 mean = mean(times_monthly_fetch_sessions, na.rm = TRUE),
 sd = sd(times_monthly_fetch_sessions, na.rm = TRUE),
 stderr = sd/sqrt(n),
 LCI = mean - qt(1 - (0.05 / 2), n - 1) * stderr,
 UCI = mean + qt(1 - (0.05 / 2), n - 1) * stderr,
 median = median(times_monthly_fetch_sessions, na.rm = TRUE),
 min = min(times_monthly_fetch_sessions, na.rm = TRUE),
 max = max(times_monthly_fetch_sessions, na.rm = TRUE),
 IQR = IQR(times_monthly_fetch_sessions, na.rm = TRUE),
 LCImed = MedianCI(times_monthly_fetch_sessions, na.rm=TRUE)[2],
 UCImed = MedianCI(times_monthly_fetch_sessions, na.rm=TRUE)[3])

# A tibble: 2 × 13
 cat_sex n mean sd stderr LCI UCI median min max IQR LCImed
 <fct> <int> <dbl> <dbl> <dbl> <dbl> <dbl> <dbl> <dbl> <dbl> <dbl> <dbl>
1 Female 537 12.7 15.5 0.670 11.4 14.0 8 0 180 17 6
2 Male 617 12.7 13.7 0.551 11.6 13.8 8 0 100 17 8
# ℹ 1 more variable: UCImed <dbl>

#count of each range in a bar graph in order
p <-ggplot(data=fetch_sessions_monthly_sex, aes(cat_sex))
p + geom_bar()


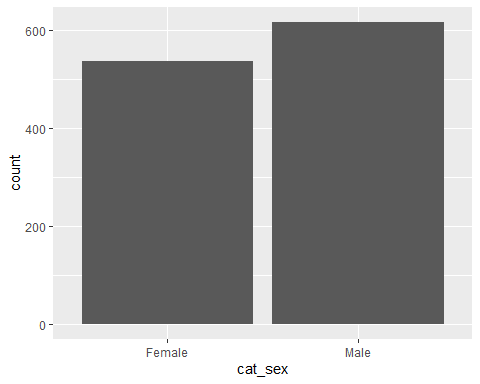


#outliers
#some outliers
ggplot(fetch_sessions_monthly_sex, aes(x = cat_sex, y = times_monthly_fetch_sessions, fill = cat_sex)) +
 stat_boxplot(geom ="errorbar", width = 0.5) +
 geom_boxplot(fill = "light blue") +
 stat_summary(fun.y=mean, geom="point", shape=10, size=3.5, color="black") +
 ggtitle("Boxplots") +
 theme_bw() + theme(legend.position="none")


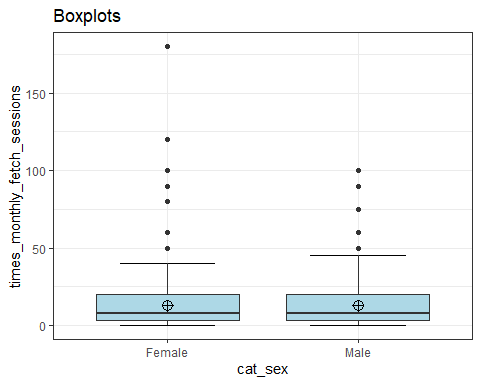


#identifies extreme outliers
#Values above Q3 + 3xIQR or below Q1 - 3xIQR are considered as extreme points (or extreme outliers)
fetch_sessions_monthly_sex %>%
 group_by(cat_sex) %>%
 identify_outliers(times_monthly_fetch_sessions) %>%
 relocate(is.extreme) %>%
 filter(is.extreme == "TRUE")

# A tibble: 12 × 4
 is.extreme cat_sex times_monthly_fetch_sessions is.outlier
 <lgl> <fct> <dbl> <lgl>
 1 TRUE Female 80 TRUE
 2 TRUE Female 120 TRUE
 3 TRUE Female 90 TRUE
 4 TRUE Female 180 TRUE
 5 TRUE Female 100 TRUE
 6 TRUE Female 100 TRUE
 7 TRUE Male 100 TRUE
 8 TRUE Male 75 TRUE
 9 TRUE Male 90 TRUE
10 TRUE Male 90 TRUE
11 TRUE Male 100 TRUE
12 TRUE Male 75 TRUE

#normality checks
#all significant so data is not normally distributed
fetch_sessions_monthly_sex %>%
 group_by(cat_sex) %>%
 summarise(`W Stat` = shapiro.test(times_monthly_fetch_sessions)$statistic,
 p.value = shapiro.test(times_monthly_fetch_sessions)$p.value)

# A tibble: 2 × 3
 cat_sex `W Stat` p.value
 <fct> <dbl> <dbl>
1 Female 0.648 9.39e-32
2 Male 0.756 3.38e-29

#QQ plots
#non-normal distributions
ggplot(data = fetch_sessions_monthly_sex, mapping = aes(sample = times_monthly_fetch_sessions, color = cat_sex, fill = cat_sex)) +
 stat_qq_band(alpha=0.5, conf=0.95, qtype=1, bandType = "boot") +
 stat_qq_line(identity=TRUE) +
 stat_qq_point(col="black") +
 facet_wrap(~ cat_sex, scales = "free") +
 labs(x = "Theoretical Quantiles", y = "Sample Quantiles") + theme_bw()


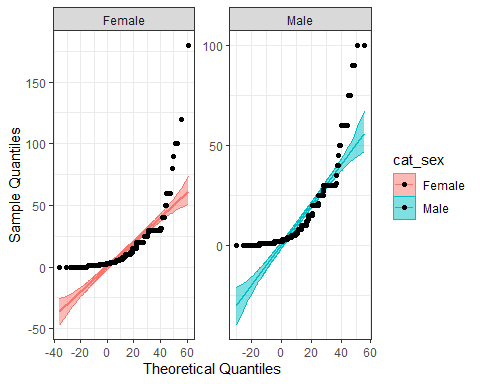


#so non-parametric tests are suitable

#Mann-Whitney test
#non-significant differences between groups
mw <- wilcox.test(times_monthly_fetch_sessions ~ cat_sex, data=fetch_sessions_monthly_sex, na.rm=TRUE, paired=FALSE, exact=FALSE, conf.int=TRUE)
print(mw)

Wilcoxon rank sum test with continuity correction

data: times_monthly_fetch_sessions by cat_sex
W = 162892, p-value = 0.6226
alternative hypothesis: true location shift is not equal to 0
95 percent confidence interval:
 -0.9999454272 0.0000754299
sample estimates:
difference in location
 -3.811416e-05

#z score of the converted W statistic
qnorm(mw$p.value/2)

[1] -0.4921931

##### excluding extreme outliers

#data of only the extreme outliers
fetch_outliers_monthly <- fetch_sessions_monthly_sex %>%
 group_by(cat_sex) %>%
 identify_outliers(times_monthly_fetch_sessions) %>%
 filter(is.extreme == "TRUE")
fetch_outliers_monthly

# A tibble: 12 × 4
 cat_sex times_monthly_fetch_sessions is.outlier is.extreme
 <fct> <dbl> <lgl> <lgl>
 1 Female 80 TRUE TRUE
 2 Female 120 TRUE TRUE
 3 Female 90 TRUE TRUE
 4 Female 180 TRUE TRUE
 5 Female 100 TRUE TRUE
 6 Female 100 TRUE TRUE
 7 Male 100 TRUE TRUE
 8 Male 75 TRUE TRUE
 9 Male 90 TRUE TRUE
10 Male 90 TRUE TRUE
11 Male 100 TRUE TRUE
12 Male 75 TRUE TRUE

#selecting columns
fetch_outliers_monthly <- fetch_outliers_monthly %>%
 select(cat_sex, times_monthly_fetch_sessions)

#then removing duplicates to exclude the extreme outliers from the dataset
fetch_monthly_outliers_excl <- anti_join(fetch_sessions_monthly_sex, fetch_outliers_monthly)

#ensuring as a factor
fetch_monthly_outliers_excl$cat_sex<-as.factor(fetch_monthly_outliers_excl$cat_sex)

#descriptive stats
fetch_monthly_outliers_excl %>% select(cat_sex, times_monthly_fetch_sessions) %>% group_by(cat_sex) %>%
 summarise(n = n(),
 mean = mean(times_monthly_fetch_sessions, na.rm = TRUE),
 sd = sd(times_monthly_fetch_sessions, na.rm = TRUE),
 stderr = sd/sqrt(n),
 LCI = mean - qt(1 - (0.05 / 2), n - 1) * stderr,
 UCI = mean + qt(1 - (0.05 / 2), n - 1) * stderr,
 median = median(times_monthly_fetch_sessions, na.rm = TRUE),
 min = min(times_monthly_fetch_sessions, na.rm = TRUE),
 max = max(times_monthly_fetch_sessions, na.rm = TRUE),
 IQR = IQR(times_monthly_fetch_sessions, na.rm = TRUE),
 LCImed = MedianCI(times_monthly_fetch_sessions, na.rm=TRUE)[2],
 UCImed = MedianCI(times_monthly_fetch_sessions, na.rm=TRUE)[3])

# A tibble: 2 × 13
 cat_sex n mean sd stderr LCI UCI median min max IQR LCImed
 <fct> <int> <dbl> <dbl> <dbl> <dbl> <dbl> <dbl> <dbl> <dbl> <dbl> <dbl>
1 Female 531 11.6 10.9 0.475 10.6 12.5 8 0 60 17 6
2 Male 611 12.0 11.5 0.464 11.1 12.9 8 0 60 17 8
# ℹ 1 more variable: UCImed <dbl>

#count of each range in a bar graph in order
p <-ggplot(data=fetch_monthly_outliers_excl, aes(cat_sex))
p + geom_bar()


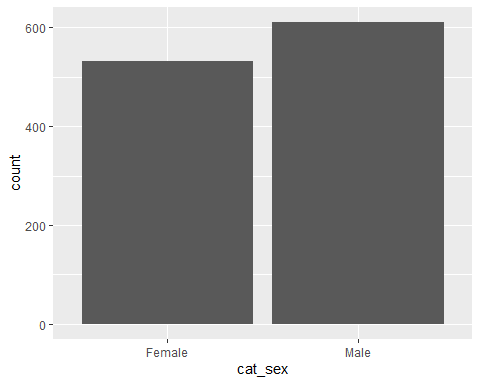


#outliers
#some outliers
ggplot(fetch_monthly_outliers_excl, aes(x = cat_sex, y = times_monthly_fetch_sessions, fill = cat_sex)) +
 stat_boxplot(geom ="errorbar", width = 0.5) +
 geom_boxplot(fill = "light blue") +
 stat_summary(fun.y=mean, geom="point", shape=10, size=3.5, color="black") +
 ggtitle("Boxplots") +
 theme_bw() + theme(legend.position="none")


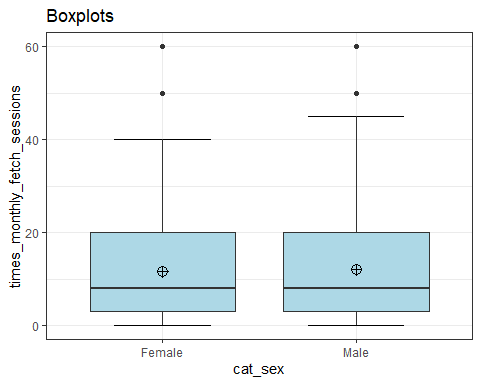


#identifies extreme outliers
#Values above Q3 + 3xIQR or below Q1 - 3xIQR are considered as extreme points (or extreme outliers)
#no extreme outliers
fetch_monthly_outliers_excl %>%
 group_by(cat_sex) %>%
 identify_outliers(times_monthly_fetch_sessions) %>%
 relocate(is.extreme) %>%
 filter(is.extreme == "TRUE")

# A tibble: 0 × 4
# ℹ 4 variables: is.extreme <lgl>, cat_sex <fct>,
# times_monthly_fetch_sessions <dbl>, is.outlier <lgl>

#normality checks
#all significant so data is not normally distributed
fetch_monthly_outliers_excl %>%
 group_by(cat_sex) %>%
 summarise(`W Stat` = shapiro.test(times_monthly_fetch_sessions)$statistic,
 p.value = shapiro.test(times_monthly_fetch_sessions)$p.value)

# A tibble: 2 × 3
 cat_sex `W Stat` p.value
 <fct> <dbl> <dbl>
1 Female 0.845 2.23e-22
2 Male 0.833 9.40e-25

#QQ plots
#non-normal distributions
ggplot(data = fetch_monthly_outliers_excl, mapping = aes(sample = times_monthly_fetch_sessions, color = cat_sex, fill = cat_sex)) +
 stat_qq_band(alpha=0.5, conf=0.95, qtype=1, bandType = "boot") +
 stat_qq_line(identity=TRUE) +
 stat_qq_point(col="black") +
 facet_wrap(~ cat_sex, scales = "free") +
 labs(x = "Theoretical Quantiles", y = "Sample Quantiles") + theme_bw()


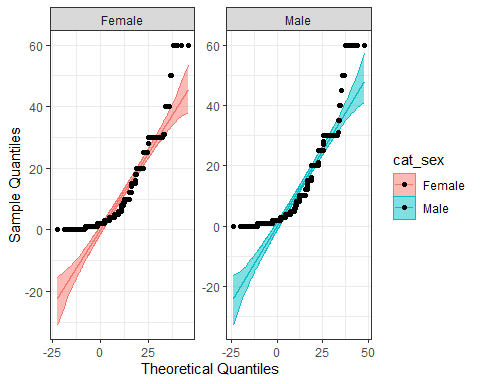


#so non-parametric tests are suitable

#Mann-Whitney test
#non-significant differences between groups
mw <- wilcox.test(times_monthly_fetch_sessions ~ cat_sex, data=fetch_monthly_outliers_excl, na.rm=TRUE, paired=FALSE, exact=FALSE, conf.int=TRUE)
print(mw)

Wilcoxon rank sum test with continuity correction

data: times_monthly_fetch_sessions by cat_sex
W = 159199, p-value = 0.5858
alternative hypothesis: true location shift is not equal to 0
95 percent confidence interval:
 -9.999622e-01 1.274996e-05
sample estimates:
difference in location
 -4.169705e-05
