# Supplementary material for: Fetching felines: a survey of cat owners on the diversity of cat (Felis catus) fetching behaviour
Source: Sci Rep. 2023 Dec 14;13:20456. doi: 10.1038/s41598-023-47409-w (PMC10721921; doi:10.1038/s41598-023-47409-w)

Thematic map version 1

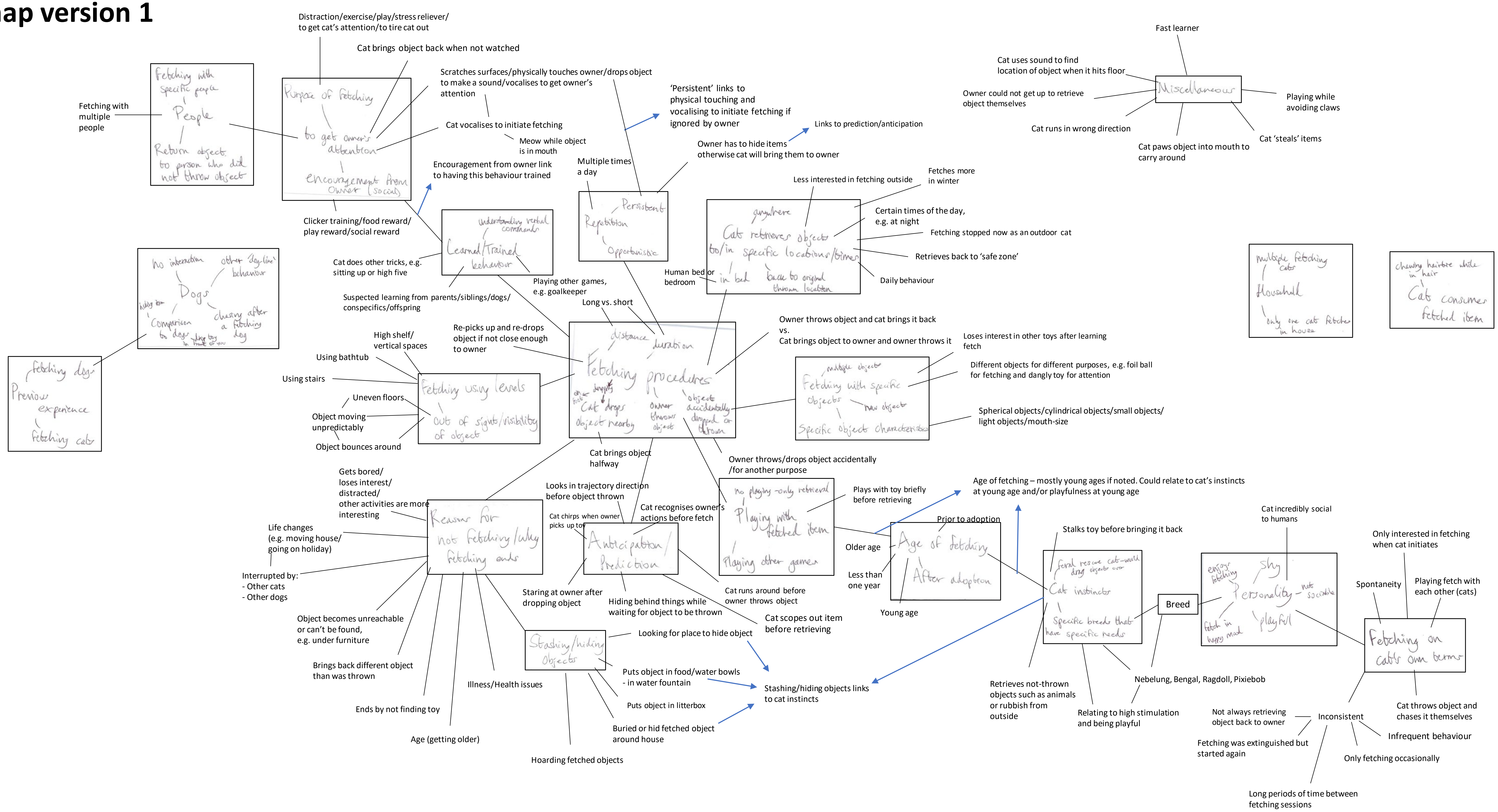

Thematic map version 2

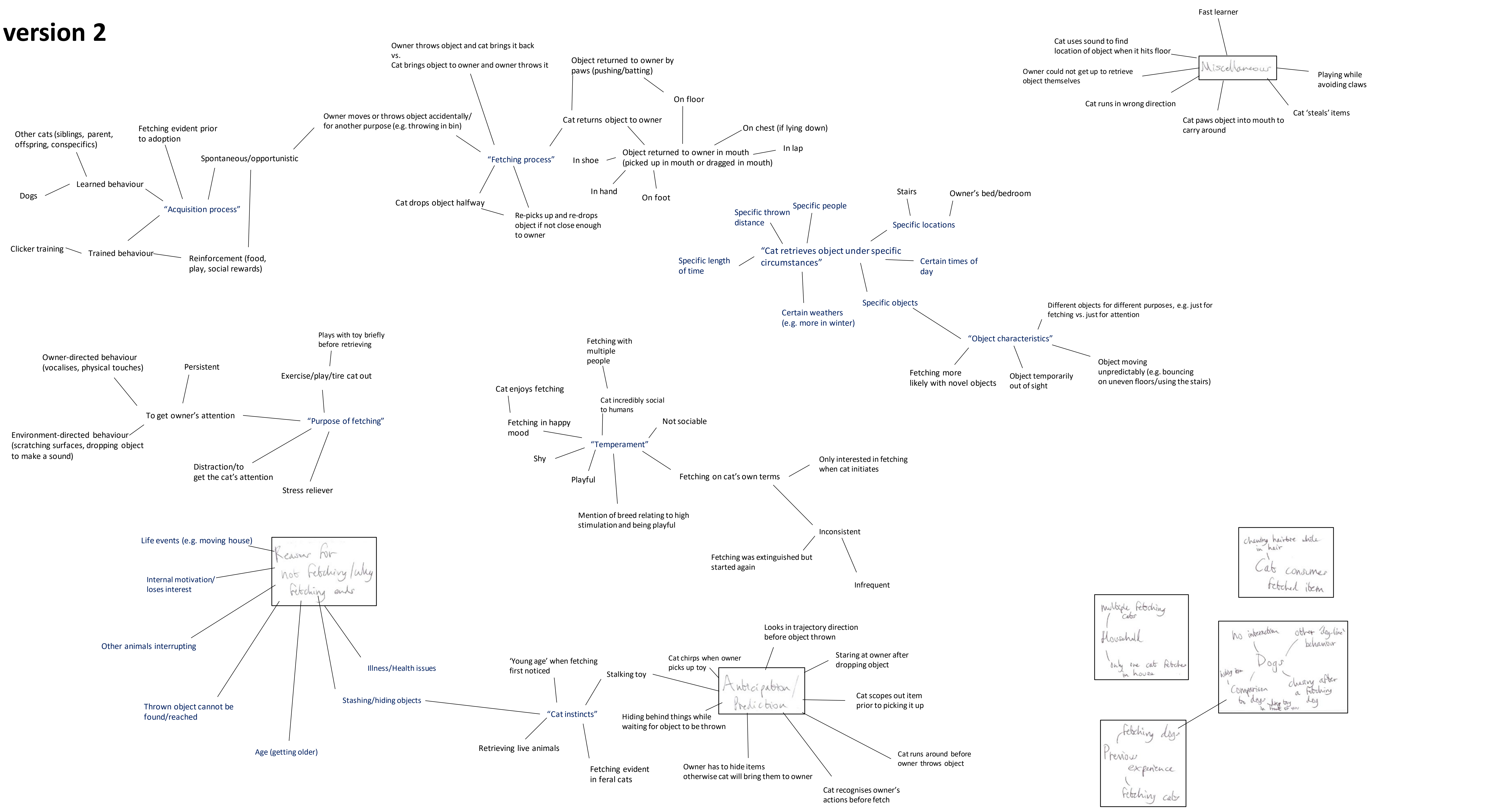

Thematic map version 3

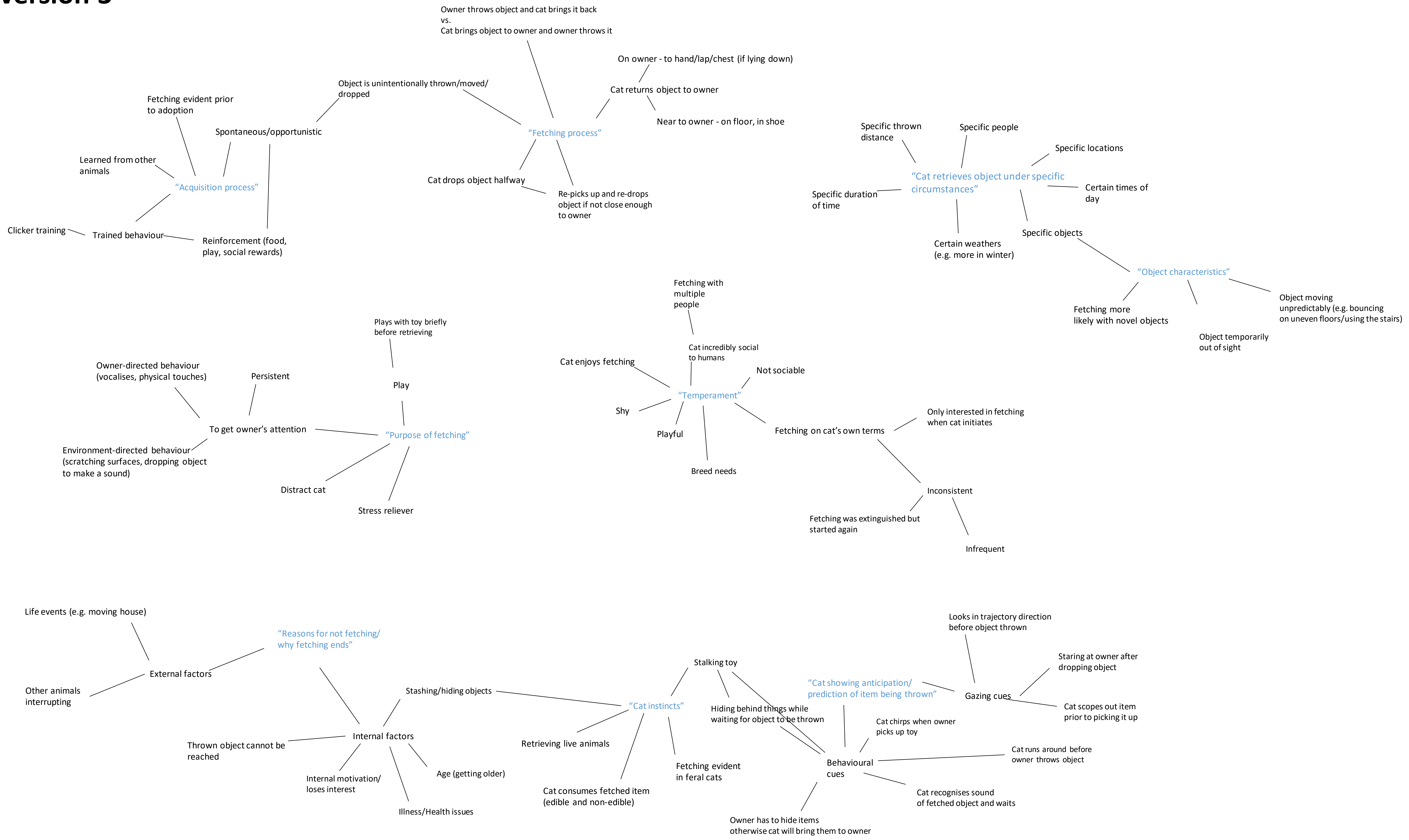

Thematic map version 4

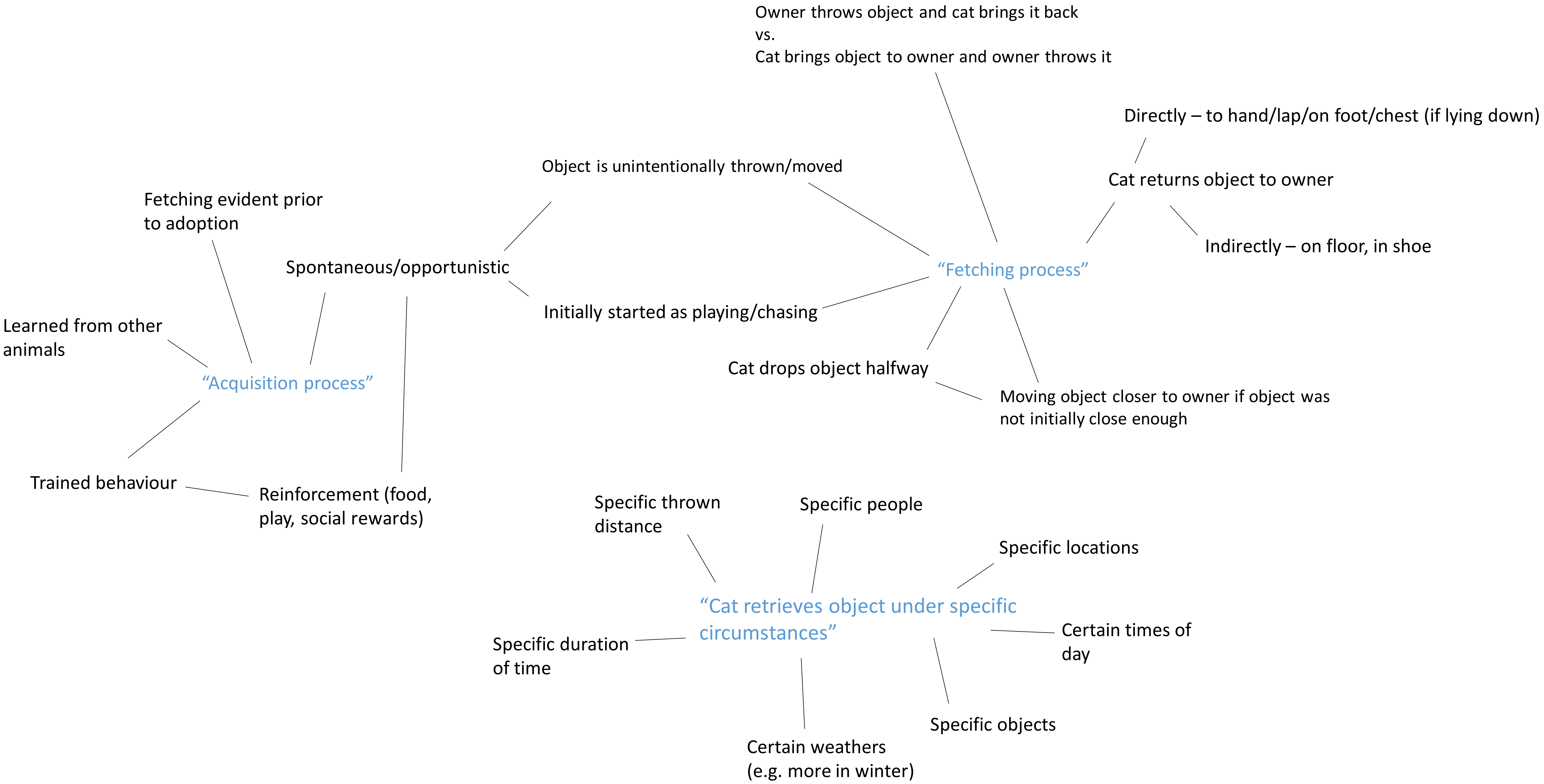

Thematic map version 5
- Final version

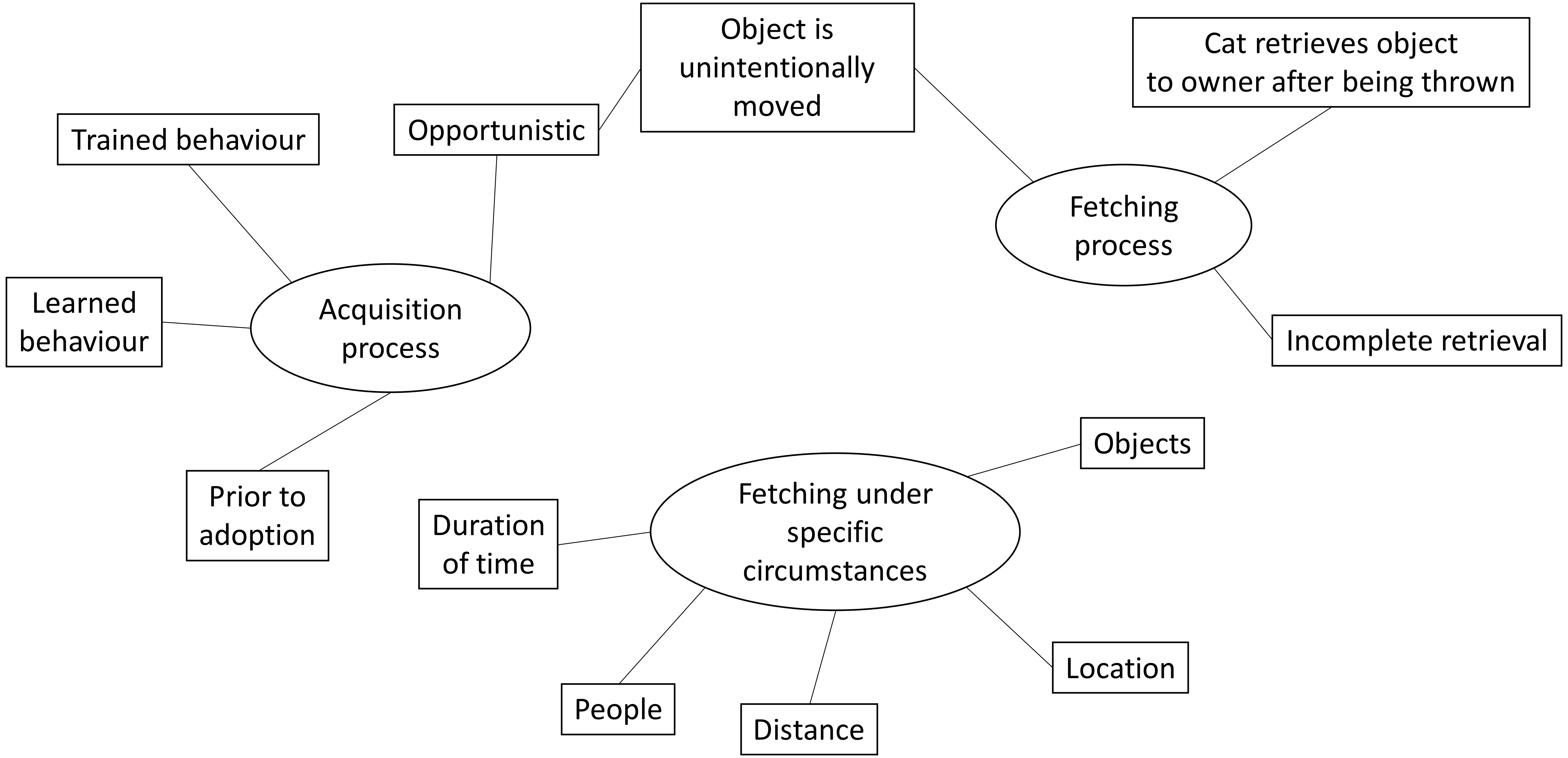

Supplement: Supplementary file 4 — Supplementary Information 4. [file 41598_2023_47409_MOESM4_ESM.pdf]
