# Supplementary material for: Fetching felines: a survey of cat owners on the diversity of cat (Felis catus) fetching behaviour
Source: Sci Rep. 2023 Dec 14;13:20456. doi: 10.1038/s41598-023-47409-w (PMC10721921; doi:10.1038/s41598-023-47409-w)
Supplement: Supplementary file 5 — Supplementary Information 5. [file 41598_2023_47409_MOESM5_ESM.docx]

| **Supplementary Table S5. Frequency of purebred cats (*N* = 160)** | |
| --- | --- |
| Siamese | 36 (22.50%) |
| Bengal | 16 (10.00%) |
| Ragdoll | 12 (7.50%) |
| Maine Coon | 11 (6.90%) |
| Nebelung | 11 (6.90%) |
| Bombay | 8 (5.00%) |
| Oriental | 8 (5.00%) |
| Russian Blue | 8 (5.00%) |
| Siberian | 8 (5.00%) |
| Burmese | 5 (3.10%) |
| Persian | 5 (3.10%) |
| Balinese | 4 (2.50%) |
| Devon Rex | 4 (2.50%) |
| Norwegian Forest Cat | 4 (2.50%) |
| Cornish Rex | 3 (1.90%) |
| Manx | 3 (1.90%) |
| Korat | 2 (1.20%) |
| Pixie Bob | 2 (1.20%) |
| Abyssinian | 1 (0.60%) |
| Angora | 1 (0.60%) |
| LaPerm | 1 (0.60%) |
| Ocicat | 1 (0.60%) |
| Ragamuffin | 1 (0.60%) |
| Savannah | 1 (0.60%) |
| Scottish Fold | 1 (0.60%) |
| Somali | 1 (0.60%) |
| Sphynx | 1 (0.60%) |
| Turkish Van | 1 (0.60%) |
