# Supplementary material for: Fetching felines: a survey of cat owners on the diversity of cat (Felis catus) fetching behaviour
Source: Sci Rep. 2023 Dec 14;13:20456. doi: 10.1038/s41598-023-47409-w (PMC10721921; doi:10.1038/s41598-023-47409-w)
Supplement: Supplementary file 7 — Supplementary Information 7. [file 41598_2023_47409_MOESM7_ESM.docx]

S7: Quotations from respondents that conceptualise the three main themes from the thematic analysis.

**Fetching process:**

Object is unintentionally moved: “When I was making dinner one evening while she was still a kitten, I dropped some macaroni; she scrambled for a piece and brought it back to me. I [praised] her enthusiastically and a night or two later dropped one to see if she'd do the same. It quickly became her favorite game.”

Cat retrieves object to owner: “Normally I’d throw toys and he would go chase after them, but would eventually lose interest and I’d need to throw another toy. However, that night, he picked up his ball and walked back in my direction, and dropped the ball at about the halfway point between me and where I originally threw it (~6 feet total distance, first dropped it ~3 feet away from me). I threw the same ball again and he repeated the behavior again, and I continued to do it and he kept bringing the ball back closer and closer to me until he eventually began dropping it right in front of me.”

“We dropped the little plastic tamper-evident ring from the top of a1 gallon plastic milk jug. She chased it around for a while, and started bringing it to us. We would throw it, and she would chase it down and bring it back”

“Julian would run to me with a cat toy carried in his mouth, and drop it at my feet, then look at my face with an excited anticipation for me to play with him. I threw the toy and he would bring it back”

“He would bring me a cat toy or something he liked, and then I would throw it to try and excite him with it, but he would bring it back. Eventually I would just play fetch with him because it seemed we both understood it was a game”

Incomplete retrieval: “She would probably fetch more if she didn't forget she had to bring me the toy to my feet instead of half way. She will eventually drop the toy further and further away from me for some reason even though she still expects me to throw it for her”

**Acquisition process:**

Prior to adoption: “Her former owner first noticed it, but told me that Tiny would bring back balls of paper on her own. The first night I brought her home I tried tossing a ball of paper and she brought it right back!”

Trained behaviour: “She would always chase the baubles but wouldn't bring them back, she would sometimes pick them up in her mouth and take them somewhere better to bat around before she'd meows at you to throw it again so I started to try catching her when she had the bauble in her mouth and calling her to come. If she came with the bauble she got the click and the treat if not she didn't, just got lots of love for coming when called. Treats were not required as motivation for very long once she understood.”

Learned behaviour: “Dexter seems to have learned fetch from watching his sister, Willow. He only recently showed an interest himself, about a year ago. He would get involved in her fetch sessions and take the object, return it to us”

“Dizzy would bring a toy (a mouse, a bundle of feathers, etc.) to me, drop it at my feet, and then attack the toy -- and my ankles. Not being terribly fond of having my ankles bitten ferociously, I would toss the toy a few feet away and encourage Dizzy to play attack it somewhere else. She'd chase it and almost inevitably bring it back, attack again, and so on. Eventually I realized she was chirping and making eye contact as she brought the toy back and (briefly) sitting patiently after she dropped it, and that her attacking my ankles was her way of asking me to get on with throwing the toy. Once I caught on -- that is to say, once she trained me -- we got into a good routine where I'd throw it, she'd chase it and bring it back and then prepare to pounce while I play-feinted and eventually threw it. Rinse, repeat.”

**Fetching under specific circumstances:**

Objects: “She's very particular about what she'll fetch, however. She'll smack anything around, but if you want to play fetch it HAS to be this one type of cosmetic swab. (It's like a Q-tip, but much thinner. Used for eyelash extensions. Tulip is interested in regular Q-tips, but she won't fetch them).”

Location: “She only likes playing in one of two places - the best place is in our bedroom, we throw the ball out of the door down the hall and into the bathroom. There’s a step she can leap over which she likes doing - the second place is from the bottom of the stairs going up into the bathroom or vice versa. If we are trying to tire her out, we both play with her throwing it up and down the stairs”

People: “Proust would fetch dogstyle squeaky mice (which were cat sized) hair ties & cloth mice, but only when Proust & I live together alone - he seems to only do this with me, as we’ve been together more than 6 years after he & I found one another, both strays in the world. When we’ve shared domiciled with human men, Proust will not fetch, & it seems as if he’s less likely to do it when he & I are alone in a shared living environment, as well.”

Duration of time: “She does not have a long attention span for fetch and gets bored easily”

“When she is after I throw the toy she will normally bring it back to me 2-3 times before she stops fetching and just sits next to the toy waiting for me to get up and walk over to throw it again”

Distance: “at the height of his interest in fetching, there seemed to be no limit to how many times he would fetch, as long as we threw the ball far enough away that he considered it 'thrown' (if we threw it and it bounced back to near to us, we had to throw it again for him to fetch it back)”
